# LCN2 promotes focal adhesion formation and invasion by stimulating c-Src activation

**DOI:** 10.1242/jcs.263663

**Published:** 2025-06-05

**Authors:** Bhagya Shree Choudhary, Nazia Chaudhary, Bushra K. Khan, Aditi Vijan, Dibita Mandal, Leena Pilankar, Shubham Gawand, Prerana Uttankar, Megha Sharma, Anusha Shivashankar, Rinki Doloi, Neha Joshi, Manjula Das, Sorab N. Dalal

**Affiliations:** ^1^ Cell and Tumour Biology, Advanced Centre for Treatment Research and Education in Cancer (ACTREC), Tata Memorial Centre, Kharghar Node, Navi Mumbai 410210, India; ^2^ Homi Bhabha National Institute, Training School Complex, Anushakti Nagar, Mumbai 400085, India; ^3^ Molecular Immunology, Mazumdar Shaw Medical Foundation, 8th floor, Mazumdar Shaw Medical Centre, Narayana Health City, Bommasandra, Bangalore 560099, India; ^4^ Center for Precision Diagnostic and Therapeutic Research, India (CPDTRI), B-202, First Floor, Block 1, KSSIDC Complex, Electronic City Phase 1, Bengaluru 560100, Karnataka, India

**Keywords:** LCN2, Focal adhesions, c-Src, Invasion, Actin

## Abstract

Previous work has demonstrated that lipocalin2 (LCN2) expression promotes invasion and migration in multiple tumor types. The mechanisms by which LCN2 promotes invasion and migration remain unclear. Previous work from our laboratory demonstrated that LCN2 promotes actin filament formation by inhibiting actin glutathionylation. In this study, we demonstrate that, in addition to inhibiting actin glutathionylation, LCN2 stimulates invasion by promoting the formation of focal adhesions, which is independent of the ability of LCN2 to bind iron (Fe^3+^). We showed that LCN2 promotes focal adhesion formation by promoting the activation of c-Src (also known as SRC) by stimulating expression of the transcription factor ETS1. ETS1, in turn, upregulates expression of the protein phosphatase PTP1B, resulting in the auto-activation of c-Src and increased paxillin phosphorylation, leading to focal adhesion formation. These results demonstrate that LCN2 has iron-dependent and -independent functions in promoting invasion and highlight the multiple mechanisms by which LCN2 promotes invasion, suggesting that c-Src inhibitors could be used to treat invasive colorectal cancer.

## Teaser

**Summary:** Expression of LCN2 is elevated in invasive colorectal cancer. We demonstrate that LCN2 promotes invasion by stimulating the formation of focal adhesions by promoting c-Src activation.

## INTRODUCTION

An increase in invasion and migration is observed in late-stage tumor cells, often leading to metastatic disease (reviewed in Chaffer et al., 2016; Lambert et al., 2017; Valastyan and Weinberg, 2011). The increase in invasion and migration depends on actin polymerization and the formation of focal adhesions (reviewed in Albiges-Rizo et al., 2009; Blanchoin et al., 2014; Case and Waterman, 2015). Although various factors stimulate focal adhesion formation (Albiges-Rizo et al., 2009; Case and Waterman, 2015), many of these processes are conserved in normal cells, making them inappropriate targets for therapeutic intervention. Hence, it is essential to study the processes regulating invasion and migration in tumor cells to identify therapeutic strategies for the treatment of invasive late-stage tumors.

In response to extracellular stimuli, actin filaments drive the formation of cellular protrusions called lamellipodia and filopodia (Le Clainche and Carlier, 2008). The leading edge of the cell interacts with the extracellular matrix (ECM) by forming focal adhesions that are connected to the intracellular lamellipodial actin network (Critchley, 2009). Focal adhesions assemble contractile actomyosin cables to generate traction force (Ciobanasu et al., 2012). The traction force generated by the contraction of stress fibers leads to the disassembly of focal adhesions and retracts the trailing edge (Ridley et al., 2003). Interaction of cells with the surrounding ECM stimulates integrin clustering, which promotes autophosphorylation of focal adhesion kinase (FAK; also known as PTK2) at Y397 to relieve auto-inhibition (Schoenwaelder and Burridge, 1999). This phosphorylation creates a docking site for activated c-Src (also known as SRC), which in turn phosphorylates FAK at Y576/577, resulting in the formation of an active FAK–c-Src complex (Calalb et al., 1995; Lietha et al., 2007). The active FAK–c-Src complex phosphorylates paxillin at Y118, resulting in focal adhesion formation (Parsons et al., 2008). c-Src is inactivated by phosphorylation at Y527, leading to inhibition of kinase activity. Y527 is dephosphorylated by the protein phosphatase PTP1B (encoded by *PTPN1*) (Zhu et al., 2007), leading to autophosphorylation at Y416 and activation of c-Src (Xu et al., 1999).

Lipocalin2 (LCN2), also known as neutrophil gelatinase-associated lipocalin (NGAL), is a secreted glycoprotein (Chakraborty et al., 2012; Schmidt-Ott et al., 2006) and is required to maintain the integrity of the gastrointestinal mucosa (Playford et al., 2006). LCN2 forms a complex with bacterial and human siderophores (reviewed in Chakraborty et al., 2012), thereby inhibiting bacterial growth and regulating iron (the ratio of Fe^2+^ to Fe^3+^) homeostasis in mammalian cells. LCN2 bound to iron is imported into cells by complex formation with the LCN2 receptors, such as SLC22A17, MCR4, LRP2 (megalin) and others (Chakraborty et al., 2012; Devireddy et al., 2005; Schroder et al., 2023). The import of iron into cells by LCN2 is required for LCN2 functions in maintaining kidney homeostasis (Mori et al., 2005; Yang et al., 2002) and regulating apoptotic cell death (Devireddy et al., 2005, 2001). LCN2 binds to and protects the matrix metalloprotease MMP9 from auto-degradation, with a concomitant increase in MMP9 activity (Yan et al., 2001). This is consistent with the observation that LCN2 can promote invasion and angiogenesis and is associated with metastasis in multiple tumor types (Chung et al., 2016; Guo et al., 2016; Lee et al., 2006; Oren et al., 2016; Tong et al., 2008).

Previous work from the laboratory demonstrated that loss of the desmosomal plaque protein plakophilin3 (PKP3), led to an increase in migration, invasion and tumor formation (Basu et al., 2018; Kundu et al., 2008). These phenotypes were dependent on the increased expression of LCN2 in the PKP3-knockdown clones (Basu et al., 2018). Further, additional data from the laboratory demonstrated that LCN2 could promote tumor progression and therapy resistance in colorectal cancer and promote invasion in colorectal cancer cell lines (Chaudhary et al., 2021; Choudhary et al., 2023). The increase in invasion was due to the ability of LCN2 to inhibit the glutathionylation of actin by regulating the levels of intracellular iron and reactive oxygen species (ROS). However, neutralizing ROS and chelating iron did not completely restore invasion in cells with low LCN2 expression, suggesting that LCN2 can stimulate invasion via other mechanisms.

The results from this study suggest that, in addition to inhibiting actin glutathionylation, LCN2 expression leads to increased focal adhesion formation. The increase in focal adhesion formation is not dependent on the ability of LCN2 to bind iron but is dependent on the LCN2-mediated activation of the tyrosine kinase c-Src, suggesting that inhibiting c-Src function in invasive colorectal cancer could be a potential therapeutic mechanism in tumors with high levels of LCN2.

## RESULTS

**Fig. 1.**
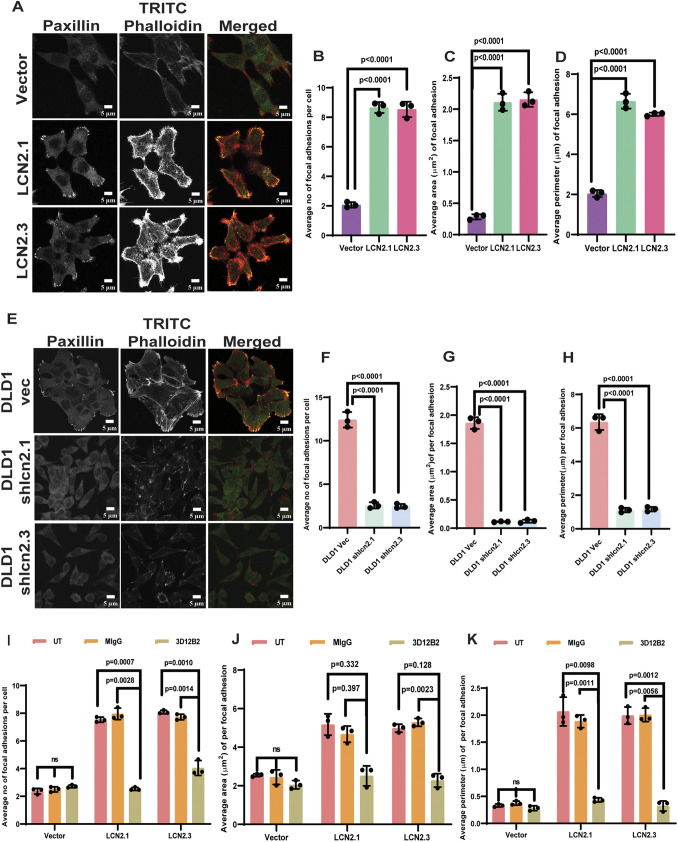
**LCN2 stimulates focal adhesion formation.** (A–H) The HCT116-derived vector control cells (vector) and LCN2-overexpressing lines (LCN2.1 and LCN2.3), and the DLD1-derived vector control (DLD1 vec) and LCN2-knockdown lines (DLD1 shlcn2.1 and DLD1shlcn2.3), were stained with antibodies against paxillin (green) or TRITC–phalloidin (red) and imaged by confocal microscopy. Representative images are shown (A,E). The number (B,F), area (C,G) and perimeter (D,H) of focal adhesions were quantitated in three independent experiments, and the mean±s.d. were plotted. (I–K) The indicated cell lines were untreated (UT) or treated with a non-specific mouse IgG (MIgG) or the anti-LCN2 antibody (3D12B2) and stained with antibodies against paxillin and TRITC–phalloidin. The number (I), area (J) and perimeter (K) of focal adhesions were determined in three independent experiments, and the mean±s.d. were plotted. Where indicated, *P*-values were determined using a paired two-tailed Student's *t*-test. Scale bars: 5 µm.

Previous results from the laboratory demonstrated that LCN2 expression can promote invasion by inhibiting the glutathionylation of actin, thus promoting actin polymerization (Choudhary et al., 2023). Because the process of invasion requires the formation of focal adhesions, we wished to determine whether LCN2 promoted focal adhesion formation. We tested this in cells with low levels of LCN2 expression (LCN2-low; HCT116), cells with LCN2 overexpression and cells with high levels of LCN2 expression (LCN2-high; DLD1), in which we inhibited LCN2 expression using vector-driven RNA interference (RNAi) (Fig. S1A,B; Choudhary et al., 2023). These cells were stained with antibodies against paxillin to identify focal adhesions and counterstained with fluorescent phalloidin. As shown in Fig. 1A,B, the LCN2-overexpressing cells showed increased numbers of paxillin foci compared to the vector control cells. The area and perimeter of the paxillin foci were increased in the LCN2-high cells compared to the vector control cells (Fig. 1C,D). In contrast, inhibiting LCN2 expression resulted in a decrease in the number, area and perimeter of paxillin foci (Fig. 1E–H). Similarly, inhibiting LCN2 function with the anti-LCN2 monoclonal antibody, 3D12B2 (Chaudhary et al., 2021; Choudhary et al., 2023), inhibited the formation of paxillin foci (Fig. S1C), and resulted in a decrease in the number, area and perimeter of paxillin foci in LCN2-overexpressing cells compared to untreated cells and cells treated with a non-specific mouse IgG (MIgG) (Fig. 1I–K). Similarly, inhibiting LCN2 expression transiently in DLD1 cells (Fig. S1D) led to a decrease in paxillin foci formation (Fig. S1E,F). These results suggested that LCN2 can promote focal adhesion formation.

**Fig. 2.**
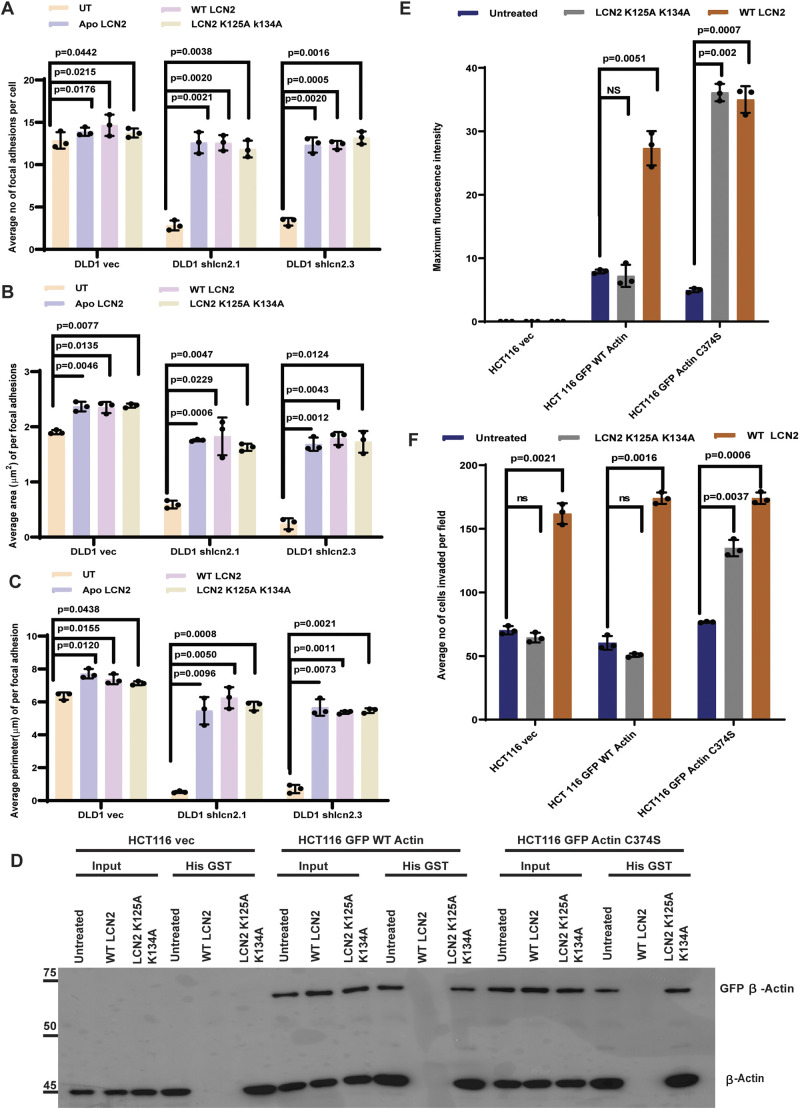
**The ability of LCN2 to bind iron (Fe^3+^) is not required for focal adhesion formation.** (A–C) The DLD1-derived LCN2-knockdown lines or the vector control cells were incubated with either vehicle control (UT), wild-type (WT) LCN2, Apo-LCN2 (not bound to iron) or the iron-binding-defective LCN2 mutant. Focal adhesion number (A), area (B) and perimeter (C) were determined in three independent experiments, and the mean±s.d. were plotted. (D–F) Cells transfected with either the vector control, GFP-WT actin or GFP-actin C374S were incubated with WT or mutant LCN2 produced in bacteria. Protein extracts were prepared and incubated with His–GST or His–LCN2, and the reactions were resolved by SDS-PAGE followed by western blotting with antibodies against actin (D). Cells were fixed and stained with TRITC–phalloidin, and the levels of filamentous actin were quantitated in three independent experiments. The mean±s.d. were plotted (E). Matrigel invasion assays were performed on the treated cells, and the number of invading cells was determined in three independent experiments. The mean±s.d. were plotted (F). Note that the presence of the C374S mutant results in an increase in filamentous actin formation and invasion in cells treated with iron-binding-defective LCN2. Where indicated, *P*-values were determined using a paired two-tailed Student's *t*-test. ns, not significant.

The ability of LCN2 to bind to iron and regulate intracellular iron levels was required for invasion stimulated by LCN2 (Chaudhary et al., 2021; Choudhary et al., 2023). To determine whether the ability of LCN2 to form a complex with iron was required for focal adhesion formation, the DLD1-derived vector control and LCN2-knockdown cells were either untreated or treated with recombinant wild-type (WT) LCN2, Apo-LCN2 (not bound to iron) or an iron-binding-defective mutant of LCN2 (K125AK134A; Bao et al., 2010), and focal adhesion formation was determined. In contrast to in the untreated cells, treatment with WT LCN2, Apo-LCN2 and the K125AK134A mutant restored focal adhesion formation in the LCN2-knockdown clones (Fig. 2A–C; Fig. S2). As our previous data suggested that the ability of LCN2 to bind iron was required to prevent the glutathionylation of actin, resulting in increased actin polymerization and invasion, we hypothesized that an actin mutant that could not be glutathionylated would promote invasion in cells treated with the iron-binding-defective mutant of LCN2. Previous work has demonstrated that the major site of glutathionylation in actin is a cysteine residue at position 374 (C374) (Wang et al., 2001). We altered this residue to serine (C374S) and tested the ability of this mutant to restore invasion in HCT116 cells treated with WT LCN2 or the K125AK134A mutant of LCN2. As a first step, we attempted to determine the level of glutathionylation in the C374S mutant. HCT116 cells were transfected with the vector control, GFP-WT actin or GFP-actin C374S, and the extracts were incubated with the purified His-tagged proteins, before reactions were purified on nickel nitrilotriacetic acid beads. Both endogenous actin and the GFP-tagged WT and C374S actin were present at equivalent levels in these assays (Fig. S3A–C). HCT116 cells transfected with the vector control showed the presence of endogenous glutathionylated actin in the His–GST pulldown in untreated cells and cells incubated with K125AK134A. In contrast, incubation with recombinant WT LCN2 resulted in a decrease in the levels of glutathionylated actin for both the endogenous actin and the exogenously expressed actin (Fig. 2D; Fig. S3D). The C374S mutant was expressed at levels similar to WT actin (Fig. 2D; Fig. S3A,C); however, no significant decrease in glutathionylation in this mutant was observed in GST-pulldown assays (Fig. 2D; Fig. S3D). This could be because actin has multiple cysteine residues that can be modified by ROS (reviewed in Wilson et al., 2016). All recombinant proteins were present at the same levels in the individual pulldowns (Fig. S3F). However, the actin C374S mutant could form actin filaments in cells treated with the iron-binding-defective LCN2 mutant, in contrast to WT actin, as demonstrated by phalloidin staining (Fig. 2E; Fig. S3G), as previously reported (Stournaras et al., 1990; Wang et al., 2001). In invasion assays, HCT116 cells transfected with WT actin showed a significant increase in invasion upon treatment with WT LCN2 but not the mutant LCN2. In contrast, cells transfected with the C374S mutant showed increased invasion in cells treated with WT and mutant LCN2 (Fig. 2F; Fig. S3H), suggesting that an actin mutant that is not glutathionylated at C374 can promote invasion in cells treated with the iron-binding-defective mutant of LCN2.

**Fig. 3.**
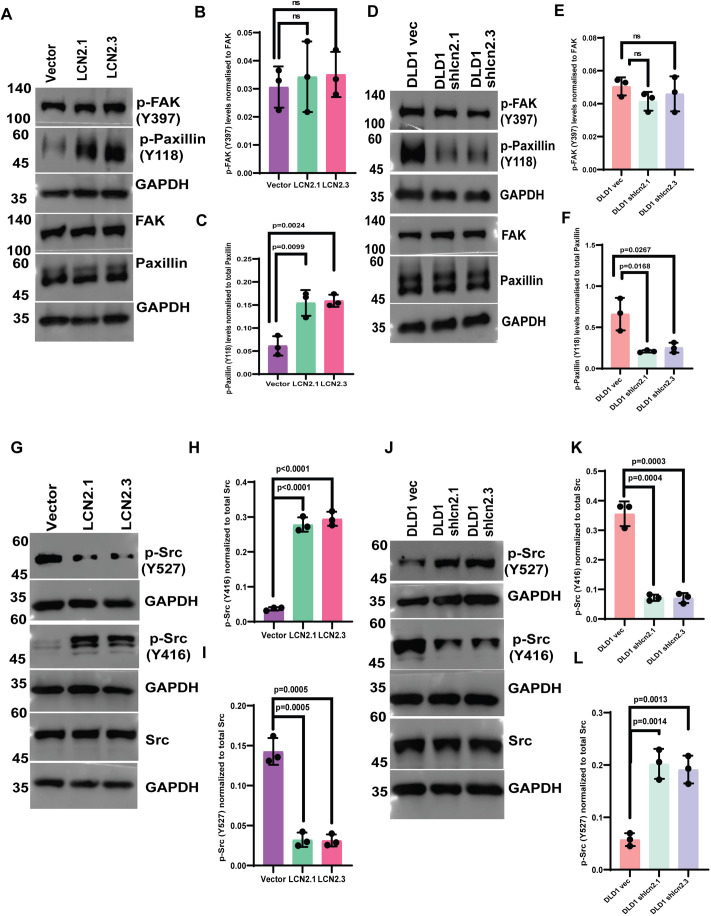
**LCN2 expression stimulates c-Src activation, leading to paxillin phosphorylation.** (A–L) Protein extracts prepared from the HCT116-derived vector control (vector) and LCN2-overexpressing lines (LCN2.1 and LCN2.3), and the DLD1-derived vector control (DLD1 vec) and LCN2-knockdown lines (DLD1 shlcn2.1 and DLD1 shlcn2.3), were resolved by SDS-PAGE followed by western blotting with the indicated antibodies. The intensity of the bands was determined using a Bio-Rad ChemiDoc, and the mean±s.d. of three independent experiments are plotted. Note that the levels of phosphorylated (p)-FAK(Y397) are unaltered in cells with either elevated or low levels of LCN2 (A,B,D,E), whereas the levels of p-paxillin (Y118) are elevated in LCN2-high cells and low in LCN2-knockdown cells (A,C,D,F). The levels of the activated c-Src (p-Src Y416) are elevated and lowered in LCN2-overexpressing and -knockdown clones, respectively (G,I,J,L), and the levels of inactive c-Src (p-Src Y527) are lowered and elevated in LCN2-overexpressing and -knockdown clones, respectively (G,H,J,K). Note that total levels of paxillin, FAK and c-Src remain unchanged. Western blots for GAPDH served as loading controls for normalization. Where indicated, *P*-values were determined using a paired two-tailed Student's *t*-test. ns, not significant.

**Fig. 4.**
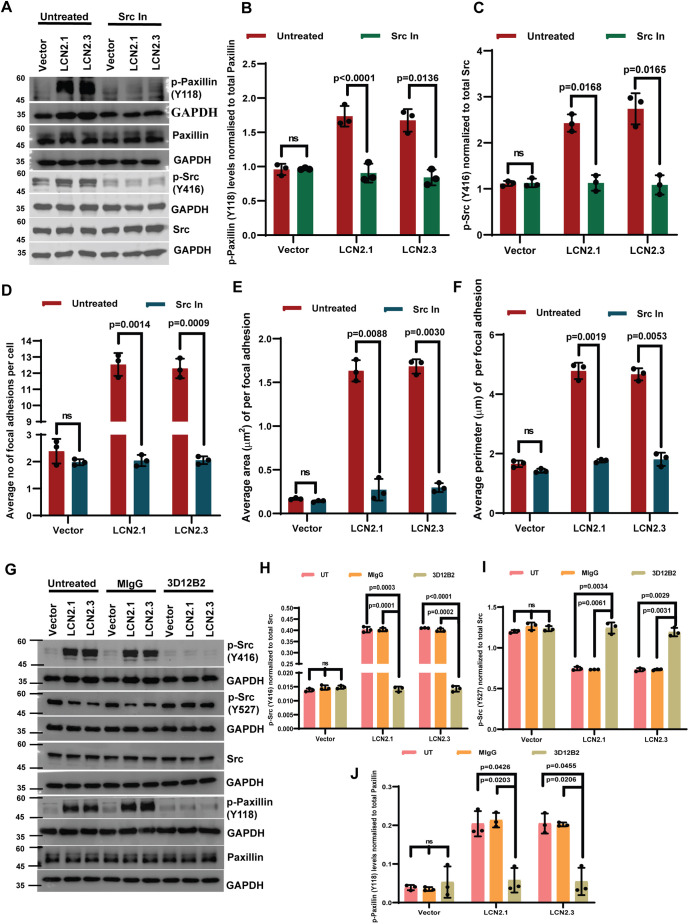
**LCN2-mediated activation of c-Src leads to focal adhesion formation.** (A–C) Protein extracts prepared from the HCT116-derived vector control (vector) and LCN2-overexpressing lines (LCN2.1 and LCN2.3) that were untreated or treated with the c-Src inhibitor were resolved by SDS-PAGE followed by western blotting with the indicated antibodies. The intensity of the bands was determined using a Bio-Rad ChemiDoc, and the mean±s.d. of three independent experiments are plotted. (D–F) The indicated cell lines were stained with antibodies against paxillin or TRITC–phalloidin and imaged by confocal microscopy. The number (D), area (E) and perimeter (F) of focal adhesions were quantitated in three independent experiments, and the mean±s.d. were plotted. (G–J) Protein extracts from cell lines that were untreated or treated with MIgG or the anti-LCN2 antibody were resolved by SDS-PAGE followed by western blotting with the indicated antibodies (G). The intensity of the bands was determined using a Bio-Rad ChemiDoc, and the mean±s.d. of three independent experiments are plotted (H–J). Note that treatment with the anti-LCN2 antibodies resulted in a decrease in the activation of c-Src and paxillin. Blots for GAPDH served as loading controls. Where indicated, *P*-values were determined using a paired two-tailed Student's *t*-test. ns, not significant.

To identify the mechanisms underlying the increased focal adhesion formation stimulated by LCN2, we determined the levels of activated FAK and paxillin in LCN2-overexpressing or -knockdown clones. Paxillin is phosphorylated at a tyrosine residue at 118 (Y118), resulting in increased formation of nascent focal adhesions (Zaidel-Bar et al., 2007), and FAK is activated by auto-phosphorylation at tyrosine 397 (Y397), leading to the formation of a docking site for activated c-Src (reviewed in Tilghman and Parsons, 2008). The binding of activated c-Src leads to phosphorylation of FAK at Y576/577, which is required for further FAK activation and phosphorylation of paxillin at Y118, resulting in increased formation of nascent focal adhesions (Zaidel-Bar et al., 2007). Western blot analysis demonstrated that although paxillin phosphorylation at Y118 was significantly elevated in LCN2-overexpressing cells and decreased in LCN2-knockdown cells, no changes were observed in the levels of FAK phosphorylated at Y397 (Fig. 3A–F). These results suggested that LCN2 has no role in relieving FAK auto-inhibition. However, it promotes nascent focal adhesion formation. Another possibility is that LCN2 might be stimulating the activation of c-Src. c-Src kinase activity is inhibited by phosphorylation at tyrosine 527 (Y527) and stimulated by phosphorylation at tyrosine 416 (Y416) (Okada, 2012; Xu et al., 1999). Western blot analysis demonstrated that the phosphorylation of c-Src at Y527 was significantly decreased, and phosphorylation at Y416 was increased, in the LCN2-overexpressing cells (Fig. 3G–I). In contrast, LCN2 knockdown led to an increase in phosphorylation at Y527 and a decrease in phosphorylation at Y416 (Fig. 3J–L). Consistent with these data, treatment with an inhibitor of c-Src led to a significant decrease in Y118 phosphorylation in paxillin and Y416 phosphorylation in c-Src in LCN2-overexpressing cells (Fig. 4A-C). The decrease in c-Src and paxillin phosphorylation upon c-Src inhibitor treatment was accompanied by a decrease in the number, area and perimeter of focal adhesions in LCN2-overexpressing cells (Fig. 4D–F; Fig. S4A) and a decrease in invasion (Fig. S4B,C). Further, treatment with the anti-LCN2 antibody led to a significant decrease in paxillin Y118 and c-Src Y416 phosphorylation and an increase in Y527 phosphorylation compared to that in untreated cells or cells treated with MIgG (Fig. 4G–J). To confirm the role of activated c-Src in focal adhesion formation, we overexpressed WT and constitutively active c-Src (c-Src Y530F) in HCT116 cells and determined the effects of expression on focal adhesion, invasion and paxillin phosphorylation. Expression of WT c-Src led to a significant increase in focal adhesion formation and invasion compared to the vector control, and a further increase was observed in cells transfected with the constitutively active c-Src (Fig. S4D–G). We observed a significant increase in the levels of the activating phosphorylations in c-Src, and in Y118 phosphorylation in paxillin, in cells transfected with c-Src Y530F and WT c-Src (Fig. S4H–J). Taken together, these results indicated that LCN2 stimulates focal adhesion formation by stimulating the kinase activity of c-Src.

**Fig. 5.**
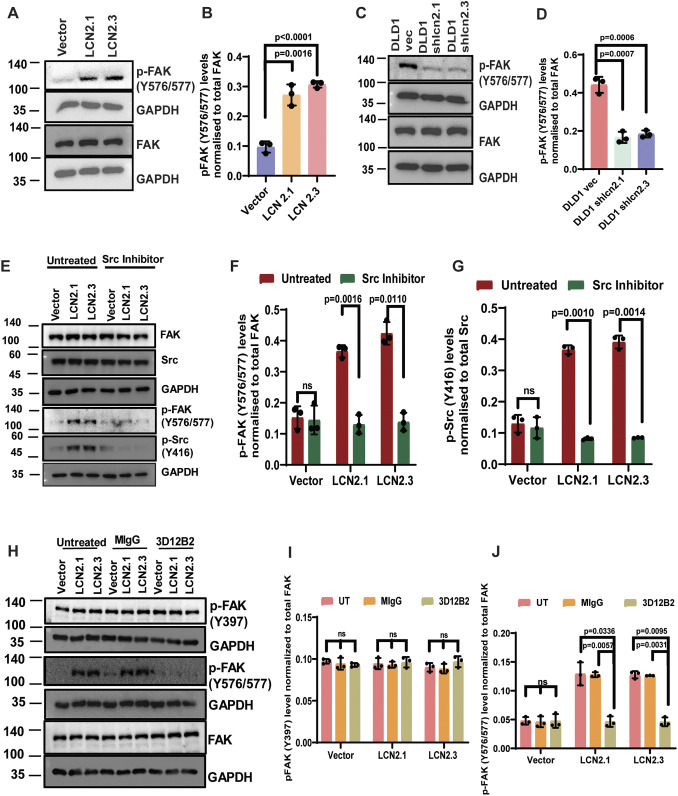
**Activation of c-Src in LCN2-expressing cells leads to increased activation of FAK.** (A–D) Protein extracts prepared from the HCT116-derived vector control (vector) and LCN2-overexpressing lines (LCN2.1 and LCN2.3), and the DLD1-derived vector control (DLD1 vec) and LCN2-knockdown lines (DLD1 shlcn2.1 and DLD1 shlcn2.3), were resolved by SDS-PAGE followed by western blotting with the indicated antibodies. The intensity of the bands was determined using a Bio-Rad ChemiDoc, and the mean±s.d. of three independent experiments are plotted. (E–G) Protein extracts prepared from the indicated cell lines that were untreated or treated with the c-Src inhibitor were resolved by SDS-PAGE followed by western blotting with the indicated antibodies. The intensity of the bands was determined using a Bio-Rad ChemiDoc, and the mean±s.d. of three independent experiments are plotted. (H–J) Protein extracts from cell lines that were untreated or treated with MIgG or the anti-LCN2 antibody were resolved by SDS-PAGE followed by western blotting with the indicated antibodies (H). The intensity of the bands was determined using a Bio-Rad ChemiDoc, and the mean±s.d. of three independent experiments are plotted (I–J). Note that treatment with the anti-LCN2 antibodies resulted in a decrease in the phosphorylation of FAK at Y576/577 but not at Y397. Blots for GAPDH served as loading controls. Where indicated, *P*-values were determined using a paired two-tailed Student's *t*-test.

**Fig. 6.**
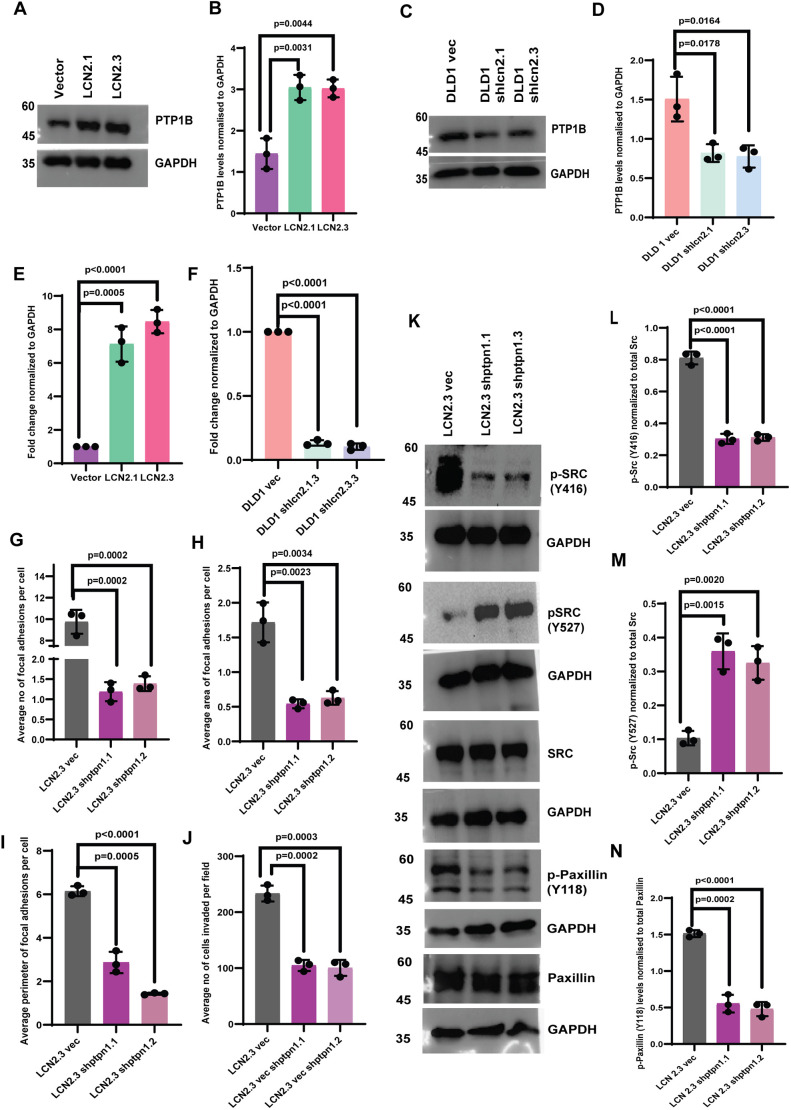
**LCN2 expression leads to an increase in PTP1B levels and c-Src activation.** (A–D) Protein extracts prepared from the HCT116-derived vector control (vector) and LCN2-overexpressing lines (LCN2.1 and LCN2.3), and the DLD1-derived vector control (DLD1 vec) and LCN2-knockdown lines (DLD1 shlcn2.1 and DLD1 shlcn2.3), were resolved by SDS-PAGE, and western blotting was performed with the indicated antibodies (A,C). The mean±s.d. of three independent experiments were plotted (B,D). (E,F) mRNA prepared from the indicated cell lines was used as a template in reverse transcriptase-coupled quantitative PCR reactions. The mean±s.d. of three independent experiments are plotted. (G–J) The LCN2.3-derived vector control (LCN2.3 vec) or PTP1B-knockdown clones (LCN2.3 shptpn1.1 and LCN2.3 shptpn1.2) were stained with antibodies against paxillin and TRITC–phalloidin, and the number (G), area (H) and perimeter (I) of focal adhesions was determined. The mean±s.d. of three independent experiments were plotted. The indicated cell lines were also used in invasion assays, and the numbers of invading cells were determined in three independent experiments. The mean±s.d. of three independent experiments were plotted (J). (K–N) Protein extracts prepared from the indicated cells were resolved by SDS-PAGE followed by western blotting with the indicated antibodies (K). The mean±s.d. of three independent experiments were plotted (L–N). Western blots for GAPDH served as a loading control. Where indicated, *P*-values were determined using a paired two-tailed Student's *t*-test.

Previous results have shown that, in addition to phosphorylation of FAK at Y397, additional phosphorylation of Y576 and Y577 in FAK by c-Src leads to the formation of an active FAK–c-Src complex and an increase in paxillin phosphorylation (Calalb et al., 1995; Lietha et al., 2007). To determine whether these phosphorylation events were stimulated upon LCN2 expression, we performed western blot analysis in cells with overexpression or knockdown of LCN2. As shown in Fig. 5A–D, the phosphorylation of these residues was increased in LCN2-overexpressing cells and decreased in the LCN2-knockdown clones. Similarly, treatment with an inhibitor of c-Src led to a decrease in activating phosphorylation in c-Src and a decrease in Y576/577 phosphorylation in FAK (Fig. 5E–G). Treatment with the anti-LCN2 antibody led to a decrease in the levels of Y576/577 phosphorylation, but not the levels of FAK397 phosphorylation, in FAK (Fig. 5H–J). As these results suggested that FAK activation was critical for focal adhesion formation, we treated cells with a chemical inhibitor of FAK, which inhibits the phosphorylation of FAK at Y397. Treatment with the FAK inhibitor led to a decrease in FAK activation (Fig. S5A,B), a decrease in focal adhesion formation (Fig. S5C,D) and a decrease in invasion (Fig. S5E,F). These results suggest that the LCN2-mediated activation of c-Src drives the activation of the FAK–c-Src complex, which is required to stimulate focal adhesion formation and invasion in cells expressing LCN2.

c-Src is activated when Y527 is dephosphorylated by the tyrosine phosphatase PTP1B (Zhu et al., 2007). PTP1B protein and *PTPN1* mRNA levels were elevated in LCN2-overexpressing cells (Fig. 6A,B,E) and decreased in LCN2-knockdown cells (Fig. 6C,D,F). Similarly, treatment with the anti-LCN2 antibody led to a decrease in the levels of PTP1B compared to those in untreated cells or cells treated with MIgG (Fig. S6A,B). To determine whether the increase in PTP1B levels in LCN2-high cells was responsible for increased c-Src activation, we generated knockdown lines for PTP1B (Fig. S6C,D), and demonstrated that focal adhesion number, area and perimeter were decreased upon PTP1B knockdown in LCN2-overexpressing cells compared to the vector control cells (Fig. 6G–I; Fig. S6E). The decrease in focal adhesion formation was accompanied by a decrease in invasion (Fig. 6J; Fig. S6F), a significant decrease in the phosphorylation of Y416 and Y118 in c-Src and paxillin, respectively, and an increase in the phosphorylation of Y527 in c-Src (Fig. 6K–N). These results suggest that LCN2 activates c-Src by upregulating the expression of PTP1B.

**Fig. 7.**
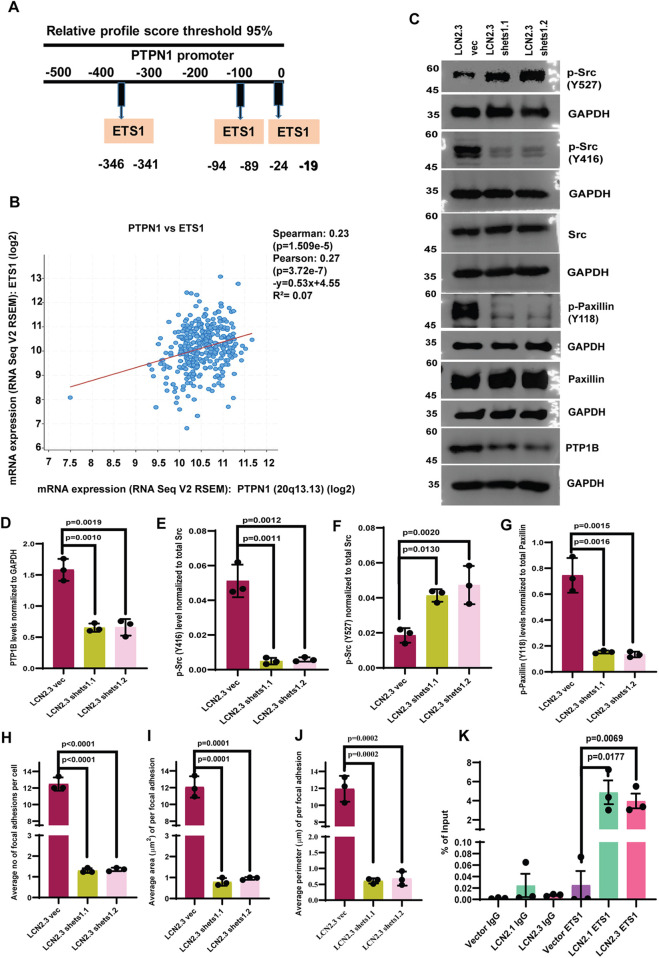
**ETS1 expression is required for the PTP1B-mediated activation of c-Src.** (A) Jasper analysis of the *PTPN1* (encoding PTP1B) promoter identifies ETS1 binding sites in the promoter. (B) Pearson's coefficient analysis of *PTPNI* and *ETS1* expression in a dataset from The Cancer Genome Atlas. The data are represented as a scatter plot in which ‘R^2^’ and ‘p’ refer to the correlation coefficient and *P*-value, respectively. (C–G) Protein extracts prepared from the LCN2.3-derived vector control (LCN2.3 vec) or ETS1-knockdown clones (LCN2.3 shets1.1 and LCN2.3 shets1.2) were resolved by SDS-PAGE followed by western blotting with the indicated antibodies (C). Band intensity was determined in three independent experiments, and the mean±s.d. are plotted (D–G). Western blots for GAPDH served as loading controls. (H–J) The LCN2.3-derived vector control (LCN2.3vec) or ETS1-knockdown clones (LCN2.3 shets1.1 and LCN2.3 shets1.2) were stained with antibodies against paxillin and TRITC–phalloidin, and the number (H), area (I) and perimeter (J) of focal adhesions were determined in three independent experiments. The mean±s.d. are plotted. (K) Chromatin immunoprecipitation assays were performed using either a non-specific rabbit IgG (IgG) or anti-ETS1 antibodies from the indicated cell, followed by quantitative PCR to detect the *LCN2* promoter. The mean±s.d. are plotted. Where indicated, *P*-values were determined using a paired two-tailed Student's *t*-test.

We have previously reported that LCN2 stimulates expression of the transcription factor ETS1 (Chaudhary et al., 2021). The LCN2-overexpressing cells showed an increase in ETS1 protein and mRNA levels, whereas the LCN2-knockdown cells showed a decrease in ETS1 protein and mRNA levels (Fig. S7A–D). Similarly, inhibiting LCN2 function with the anti-LCN2 antibody led to a significant decrease in ETS1 levels compared to those in the untreated cells or cells treated with MIgG (Fig. S7E,F). A Jasper analysis demonstrated that the promoter of the PTP1B-encoding gene (*PTPN1*) contained binding sites for ETS1 (Fig. 7A). A correlation was observed between the expression of *PTPN1* and *ETS1* in colon adenocarcinoma samples (Fig. 7B). To confirm the contribution of ETS1 to PTP1B expression, we inhibited ETS1 expression in the LCN2-overexpressing cells using previously described shRNA constructs for *ETS1* (Chaudhary et al., 2021) (Fig. S7G,H). Loss of ETS1 led to a significant decrease in the levels of PTP1B, and phosphorylation of c-Src and paxillin at Y416 and Y118, respectively, which was accompanied by an increase in the levels of Y527 phosphorylation in c-Src (Fig. 7C–G). This was accompanied by a significant decrease in the number, area and perimeter of focal adhesions (Fig. 7H–J), leading to decreased invasion compared to that in the vector control cells (Fig. S7I,J). Finally, chromatin immunoprecipitation assays demonstrated significantly increased occupancy of the *PTPN1* promoter by ETS1 in LCN2-overexpressing cells compared to that in the vector control cells (Fig. 7K). These results suggest that LCN2 expression leads to an increase in c-Src activation by upregulating the expression of ETS1, leading to increased PTP1B expression.

EGFR has been reported to stimulate the expression of ETS1 (Phuchareon et al., 2015; Tetsu et al., 2015, 2016). Previous reports have suggested that a secretion-defective mutant of LCN2 promotes the recycling of EGFR to the cell border, resulting in further EGFR activation, implying that LCN2 has intracellular functions (Yammine et al., 2019). To determine whether a secretion-defective mutant of LCN2 can stimulate the activation of FAK and c-Src, we generated a recombinant version of the LCN2 mutant with deletion of the N-terminal secretion signal (LCN2Δss) and tested the ability of this mutant to induce FAK and c-Src activation. As shown in Fig. S8A,B, WT LCN2, the K125AK134A mutant and the LCN2Δss mutant entered HCT116 cells. Western blot analysis indicated that all the recombinant proteins could induce the phosphorylation of c-Src at Y416 and FAK at Y576/577, in contrast to cells treated with the vehicle control (Fig. S8C–E). However, although WT LCN2 could stimulate invasion, in comparison to the vehicle control, the iron-binding defective mutant could not stimulate invasion. The secretion-defective LCN2 mutant stimulated some invasion, probably because it can still sequester iron inside the cell but cannot export iron out of the cell (Fig. S8F,G). These results suggest that intracellular LCN2 stimulates focal adhesion formation.

## DISCUSSION

The data shown here demonstrate that LCN2 promotes invasion in two ways: the first by preventing actin glutathionylation, allowing actin polymerization, and the second by promoting focal adhesion formation. The first is dependent on the ability of LCN2 to sequester iron, thereby reducing ROS levels, and the second is dependent on the ability of LCN2 to promote the activity of c-Src, leading to focal adhesion formation. Both LCN2 functions are required for the invasion of colon cancer cells.

Previous work from multiple laboratories has demonstrated that LCN2 promotes invasion and metastasis in multiple tumor types (Basu et al., 2018; Ding et al., 2015; Guo et al., 2014; Leng et al., 2009; Miyamoto et al., 2016; Oren et al., 2016; Yang et al., 2009). Further, Sun et al. (2011) demonstrated that increased LCN2 levels correlated with poor survival in people with colorectal cancer at all stages, and that increased LCN2 levels correlated with increased invasion in the cecum and increased metastasis in mouse xenograft models (Sun et al., 2011). The ability of LCN2 to promote invasion has been ascribed to the ability of LCN2 to promote epithelial–mesenchymal transition (Ding et al., 2015; Yang et al., 2009) and to promote angiogenesis (Yang et al., 2013), suggesting that LCN2 could be a potential therapeutic target in multiple tumor types (Leng et al., 2009; Miyamoto et al., 2016). Indeed, potential therapeutic agents that inhibit LCN2 function show anti-tumor potential and inhibit invasion (Chaudhary et al., 2021, 2023; Guo et al., 2016, 2014; Leng et al., 2009; Yao et al., 2021). LCN2 levels can be stimulated by increased Her2 (also known as ERBB2) activity (Leng et al., 2009) and an increase in p38 (also known as MAPK) activity (Basu et al., 2018), and both lead to increased invasion and tumor formation. The results in this paper demonstrate that, in addition to stimulating the polymerization of actin by inhibiting actin glutathionylation, LCN2 promotes the formation of focal adhesions by stimulating c-Src activity, leading to increased invasion and migration.

Excessive iron leads to increased tumor progression in human colorectal cancer (Nelson, 2001) and in mouse models of colorectal cancer (Radulescu et al., 2012). Increased iron levels lead to the generation of ROS (Toyokuni, 1996), which are associated with colorectal cancer progression (Myant et al., 2013; Wang et al., 2017). However, high ROS levels inhibit the polymerization of G-actin, and high levels of ROS lead to the cleavage of actin filaments (Xu et al., 2017). Further, ROS can induce the glutathionylation of cysteine 374 in actin, leading to a decrease in filament stability and reduced polymerization (Stournaras et al., 1990; Wang et al., 2001). C374 glutathionylation is exacerbated in the presence of iron in the neurological disorder Friedreich's ataxia, leading to a decrease in migration (Pastore et al., 2003). All of these results suggest that increased ROS levels inhibit actin polymerization and invasion. However, multiple pieces of evidence indicate that invasion can be stimulated by the accumulation of ROS at the invasive front in tumor samples and in cells grown *in vitro* (Gianni et al., 2011, 2010; Giannoni et al., 2005; Gozin et al., 1998). Increased ROS is associated with the increased formation of invadopodia (Gianni et al., 2010) and increased activation of FAK and c-Src (Giannoni et al., 2005; Gozin et al., 1998). These differences could be ascribed to the local accumulation of specific ROS species in cellular compartments and also to differences in total ROS (Cheung and Vousden, 2022; Tochhawng et al., 2013) and labile iron levels (Toyokuni, 1996). Our results indicate that in a high-iron environment, such as the colon, tumor cell invasion might be dependent on the ability of the tumor cell to clear ROS species by decreasing iron levels, thereby leading to a decrease in actin glutathionylation and increased actin polymerization.

Our results suggest that the actin mutant C374S, while not showing a great decrease in glutathionylation, shows increased polymerization and supports an increase in invasion in cells treated with the LCN2 K125AK134A mutant, which is defective for iron binding. A previous report has suggested that multiple cysteine residues in actin are glutathionylated (Wilson et al., 2016), which might be the reason why we do not see any significant differences in glutathionylation between WT actin and the C374S mutant. It is possible that glutathionylation at other cysteine residues in actin is elevated in the C374S mutant. However, as previously reported, glutathionylation of C374 is the major modification that inhibits actin polymerization (Stournaras et al., 1990), which is consistent with the findings in this paper. Our results also suggest that although the ability of LCN2 to bind iron is required for invasion and inhibition of actin glutathionylation, leading to the generation of actin fiber formation to generate the forces required for invasion (Choudhary et al., 2023), iron binding by LCN2 is dispensable for the formation of focal adhesions. This is important as this is the first iron-independent function of LCN2 that has been reported and opens up the possibility that there are other LCN2 functions required for tumor formation that are independent of the ability of LCN2 to regulate iron homeostasis. These results also suggest that these two independent functions of LCN2 are required for invasion.

There is a wealth of literature suggesting that although focal adhesions promote actin polymerization, stress fiber formation also promotes focal adhesion maturation (reviewed in Case and Waterman, 2015). In the models used in this study, we have demonstrated that the iron-binding-defective mutant of LCN2 can promote focal adhesion formation but not actin polymerization (Choudhary et al., 2023). However, there is evidence that suggests that actin fiber formation drives the formation of mature focal adhesions containing zyxin (Choi et al., 2008; Hirata et al., 2008). We have not performed staining for zyxin, and it is possible that, in the absence of actin filament polymerization, we would not observe the formation of mature focal adhesions.

Previous studies have suggested that c-Src phosphorylates ETS1, resulting in increased stabilization of ETS1 as it is not degraded by the E3 ligase COP1, leading to increased tumor progression (Lu et al., 2014). Further, ETS1 is required for resistance to sorafenib in hepatocellular carcinoma by disrupting mitochondrial ROS (Vishnoi et al., 2022), leading to increased invasion. These data are consistent with our results, which suggest that LCN2 promotes the activation of c-Src by stimulating the expression of ETS1, leading to increased expression of the protein phosphatase PTP1B. PTP1B dephosphorylates c-Src at Y527, resulting in increased activation of c-Src, leading to increased paxillin phosphorylation, focal adhesion formation and increased invasion. These results are consistent with previously published data suggesting that LCN2 activates c-Src (Zhang et al., 2021). Additionally, we demonstrate that c-Src leads to increased phosphorylation of FAK at Y576/577, which has been reported to activate the FAK–c-Src complex. This activated FAK–c-Src complex phosphorylates paxillin at Y118 to initiate nascent focal adhesion formation (Calalb et al., 1995; Lietha et al., 2007). Inhibition of c-Src leads to a decrease in FAK phosphorylation at Y576/577, paxillin phosphorylation at Y118, focal adhesion number and invasion. Similarly, inhibition of FAK activity using a chemical inhibitor also leads to a decrease in focal adhesion formation and invasion. Further, we previously reported that the increase in ETS1 levels stimulated by LCN2 expression is required for clearance of ROS (Chaudhary et al., 2021) and that ROS clearance by LCN2 is required to prevent actin glutathionylation and promote actin polymerization (Choudhary et al., 2023). Thus, in addition to promoting the formation of an active FAK–c-Src complex, in which FAK is additionally phosphorylated at Y576/577 (Parsons et al., 2008), leading to focal adhesion formation, LCN2 also promotes actin polymerization by decreasing the levels of ROS.

Multiple experiments have shown that LCN2 cycles between the extracellular space and is re-imported into cells through interaction between LCN2 and multiple cell surface receptors of LCN2 (Chakraborty et al., 2012; Devireddy et al., 2005, 2001). This secretion and subsequent re-import of LCN2 into cells is required for the ability of LCN2 to regulate intracellular iron levels. Under the conditions of low iron in the extracellular milieu, LCN2 import results in increased iron availability inside the cell (Chi et al., 2020; Devireddy et al., 2005). In contrast, an increase in intracellular iron levels can stimulate ferroptosis, which is inhibited by the activation of iron export and a reduction in iron import (Gao et al., 2015; Ma et al., 2016). Our previous publication has shown that treatment with the anti-LCN2 antibody results in both an increase in the levels of intracellular iron (Chaudhary et al., 2021) and in actin glutathionylation, a consequence of increased intracellular iron levels, resulting in a decrease in actin filament formation and invasion (Choudhary et al., 2023). Our work strongly suggests that the anti-LCN2 antibody prevents the interaction of extracellular LCN2 with its receptors, thereby preventing LCN2 transport into the cell, resulting in increased intracellular iron.

Previous data have demonstrated that, in a model of chronic kidney disease, LCN2 stimulates the recycling of activated EGFR to the cell border (Yammine et al., 2019), leading to increased EGFR activity; importantly, this is independent of LCN2 secretion as an LCN2 mutant that is not secreted can still stimulate the recycling of EGFR. EGFR activation leads to the activation of several downstream transcription factors, including ETS1, leading to the hypothesis that ETS1 inhibition might be a way to overcome resistance to EGFR inhibition (Phuchareon et al., 2015; Tetsu et al., 2015, 2016). The increase in ETS1 levels in LCN2-high cells might be due to the increased cycling of EGFR to the cell border. Similarly, loss of LCN2 in oral cancer cell lines leads to a decrease in EGFR recycling and a decrease in invasion (Huang et al., 2023). These results suggest that one possible mechanism by which intracellular LCN2 might function is by regulating the localization and recycling of activated EGFR.

We have demonstrated that the recombinant LCN2 proteins used in this study enter the cell, presumably through interaction with the LCN2 receptors (Fig. S8; Choudhary et al., 2023). Therefore, these proteins could regulate intracellular events. We have generated a recombinant secretion defective mutant (LCN2Δss) of LCN2, which removes the signal sequence required for export, as described in Yammine et al. (2019). We have treated HCT116 cells with either the vehicle control, the WT LCN2, the iron-binding-defective mutant of LCN2 or the secretion-defective mutant of LCN2. We have demonstrated that all recombinant proteins enter the cell, and all of them stimulate phosphorylation of c-Src at Y416 and FAK at Y576/577. However, only WT LCN2 promotes a significant increase in invasion, presumably because the iron-binding-defective and secretion-defective LCN2 mutant proteins cannot promote the export of iron outside the cell. These data strongly suggest that cellular LCN2 has a role in promoting focal adhesion formation, as suggested by the model. Finally, a previous study suggested that a secretion-defective LCN2 mutant can promote the recycling of activated EGFR to the cell border (Yammine et al., 2019), and there are multiple studies that suggest that activation of EGFR can promote the expression of multiple transcription factors including ETS1 (Phuchareon et al., 2015; Tetsu et al., 2015, 2016). These results support the argument that LCN2 promotes iron export by binding to iron and exporting it out of the cell and upregulates ETS1 expression to facilitate focal adhesion formation. However, it is also possible that interaction of LCN2 with the LCN2 receptor could contribute to the signaling events we have explored in this study.

**Fig. 8.**
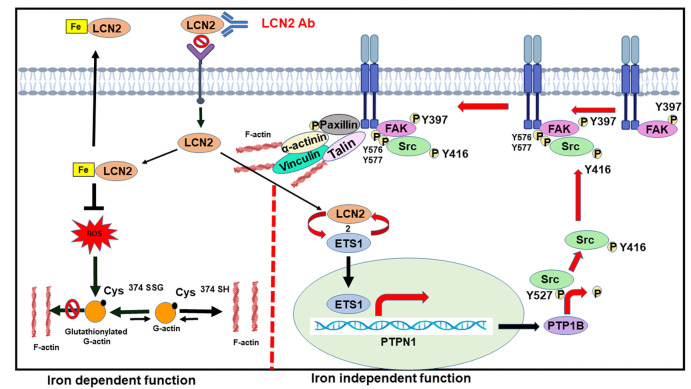
**Model for how LCN2 stimulates invasion.** LCN2 inhibits invasion in two ways: by preventing the glutathionylation of actin at C374 and by stimulating the expression of ETS1, which upregulates the expression of the phosphatase PTP1B, thereby activating c-Src and promoting focal adhesion formation. Inhibition of actin glutathionylation requires the ability of LCN2 to bind iron, and the increase in expression of ETS1 is independent of the ability of LCN2 to bind iron.

Based on the results presented in this paper, we propose the following model (Fig. 8). LCN2 promotes invasion in two ways: the first is by stimulating actin polymerization by preventing the glutathionylation of G-actin, which requires the ability of LCN2 to bind iron and inhibit ROS generation, and the second is that LCN2 promotes focal adhesion formation. Intracellular LCN2 leads to an increase in the expression of ETS1, leading to c-Src activation and focal adhesion formation in a manner that does not require the ability of LCN2 to bind iron or be secreted out of the cell. This is the first report of an LCN2 function that is not dependent on the ability of LCN2 to bind iron. Both functions of LCN2 are required to promote invasion, and LCN2 expression is associated with increased tumor stage in colorectal cancer (Chaudhary et al., 2021) and present in 60–70% of colorectal cancers (Chaudhary et al., 2021; Zhang et al., 2009). Thus, combining therapeutics targeting LCN2 and c-Src in invasive tumors might result in better patient outcomes and improved responses to chemotherapy in colorectal cancer.

## MATERIALS AND METHODS

### Cell lines and transfections

HCT116 cells (RRID: CVCL_0291) were procured from Dr Bert Vogelstein (Johns Hopkins Medical School, Baltimore, MD, USA), and DLD-1 cells (RRID: CVCL_0248) were procured from Dr Sanjeev Galande (Shiv Nadar University, Greater Noida, India), and cultured as described (Choudhary et al., 2023). Cell lines were authenticated by short tandem repeat (STR) profiling during the past 3 years. Mycoplasma-free cells were used for all the experiments. The HCT116-derived LCN2-overexpressing lines (LCN2.1 and LCN2.3) and vector control cells (PTPCD1), and the DLD1-derived LCN2-knockdown clones (DLD1 shlcn2.1 and DLD1 shlcn2.3) and vector control cells (DLD1 vec), were previously described (Choudhary et al., 2023). The vector control and PTP1B-knockdown clones were generated in LCN2.3 by transfecting these cells with an shRNA construct against PTP1B and maintained in 0.5 μg/ml puromycin (Sigma, 8833) ETS1 knockdowns were performed as described (Chaudhary et al., 2021). To determine the role of c-Src and FAK in invasion, migration and focal adhesion formation, LCN2-overexpressing cells were treated with 10 µM of the c-Src inhibitor (Sigma, S-2075) or 1 µM of the FAK inhibitor (Sigma, PF-573228) or vehicle control (DMSO) for 24 h prior to the assays being performed.

### Plasmids

The shRNA constructs for LCN2 and ETS1 have been described previously (Chaudhary et al., 2021). The human c-Src constructs pDEST40-2XHA-wtSrc (Addgene plasmid #140294) and pDEST40-2XHA-Src-Y530F (Addgene plasmid #140318) were deposited by Mark Moasser (Spassov et al., 2018). The oligonucleotide sequences used to generate the *PTPN1* shRNA, C374S mutant and LCN2Δss mutant are in Table S1. The product was amplified and cloned into pET28b using BamHI and XhoI. The WT and K125AK134A mutants are previously described (Chaudhary et al., 2021).

### Antibodies and western blot analysis

Whole-cell protein extracts were made either in EBC lysis buffer (made in house) or in 1× sample buffer as described (Basu et al., 2015). Proteins from the cell supernatant were precipitated in acetone, and lysate was prepared in 1× sample buffer for western blots (Chaudhary et al., 2021). Dilutions of antibodies are shown in Table S2. The blots were developed using either Super Signal West Pico Chemiluminescent Substrate (Pierce), Super Signal West Femto Chemiluminescent Substrate (Pierce) or Clarity Western ECL substrate (Bio-Rad) as per the manufacturers’ instructions. The blots were visualized on a Bio-Rad Chemidoc and the images quantifed using the Image lab software. The anti-LCN2 antibody, 3D12B2, was generated and purified as described (Chaudhary et al., 2021). We used 5 µg of the anti-LCN2 antibody or MIgG to treat the cells for 24 h prior to performing the assays. Similarly, we used 5 µg of the exogenous LCN2 proteins Apo-LCN2, WT LCN2 and mutant LCN2 for 24 h prior to the assays. Western blots used for generation of the figures in this paper are in Fig. S9.

### Immunofluorescence and invasion assays

Immunofluorescence was performed as described previously (Basu et al., 2018). Dilutions used for primary and secondary antibodies are in Table S3. Confocal images were obtained using an LSM780 Carl Zeiss confocal microscope and 63× objective with 2× optical zoom. The quantitation of paxillin streaks was performed using Fiji software. Paxillin streaks were measured by quantitating points having maximum intensity using the find maxima tool in Fiji software after setting the prominence at 80. The area and perimeter of the paxillin streak were measured by drawing a region of interest around individual streaks. 30 cells were used to quantify the paxillin streak number, area and perimeter in three independent experiments. Matrigel invasion assays were performed using Boyden chambers as described previously (Basu et al., 2018). Briefly, 2×10^5^ cells that were untreated or treated with the indicated inhibitors or 5 µg of recombinant proteins were added to the upper chamber in serum-free medium, while the lower chamber contained complete medium. After 24 h, the invading cells were stained with 0.5% Crystal Violet, and the number of invading cells was determined from ten fields in three independent experiments.

### Actin glutathionylation assays

HCT116 cells were transfected with 1 µg of either pEGFP actin vector (Clontech, 6116-1) or pEGFP actin C374S (generated in house) or pEGFP N1 vector (Clontech, 6085-1) and selected in 5 µg/ml neomycin (Sigma, A1720) for 10 days. Actin glutathionylation using the His–GST protein was determined as described previously (Choudhary et al., 2023), and the maximum fluorescent intensity of actin filaments was measured with Fiji software. We used 20 µg of the total protein lysate for the input and 67 µg of the total protein for the actin pulldown. The quantitation for His–GST–actin pulldown was done using Image Lab software (Bio-Rad). Pulldown of His–GST–actin was normalized to the actin in the input and converted to a percentage.

### Chromatin immunoprecipitation assays

Cell lines grown to 60–70% confluence in 10 cm culture dishes were used and fixed with 1% (v/v) formaldehyde for 10 min at room temperature, followed by quenching with 0.25 M glycine. The cells were sonicated to lyse and fragment chromatin to size (200–600 bp). 5% of the lysed chromatin served as input for control, and the rest of the lysate was equally divided to be used for immunoprecipitation with either non-specific IgG or the anti-ETS1 antibody. The DNA eluted from the immunoprecipitated lysate was used in RT-PCR with primer against *PTPN1*. The oligonucleotides used for the chromatin immunoprecipitation experiments are in Table S1. The percentage of pulled down chromatin was determined using the amount of input DNA.

### Bioinformatics analysis

cBioPortal software (https://www.cbioportal.org) was used to determine the correlation between *ETS1* and *PTPN1* by retrieving the RNA-sequencing data of 342 samples for colorectal adenocarcinoma from a dataset from The Cancer Genome Atlas. In cbioPortal, the mRNA expression levels were calculated using RNA seq V2 RSEM and aligned to the reference genome. RSEM (RNA Seq by Expectation Maximization) is an algorithm used to estimate the expression levels with 95% credibility intervals.

## Supplementary Material



10.1242/joces.263663_sup1 Supplementary information
