# Supplementary material for: LCN2 promotes focal adhesion formation and invasion by stimulating c-Src activation
Source: J Cell Sci. 2025 Jun 5;138(11):jcs263663. doi: 10.1242/jcs.263663 (PMC12188317; doi:10.1242/jcs.263663)
Supplement: Supplementary information [file joces-138-263663-s1.pdf]

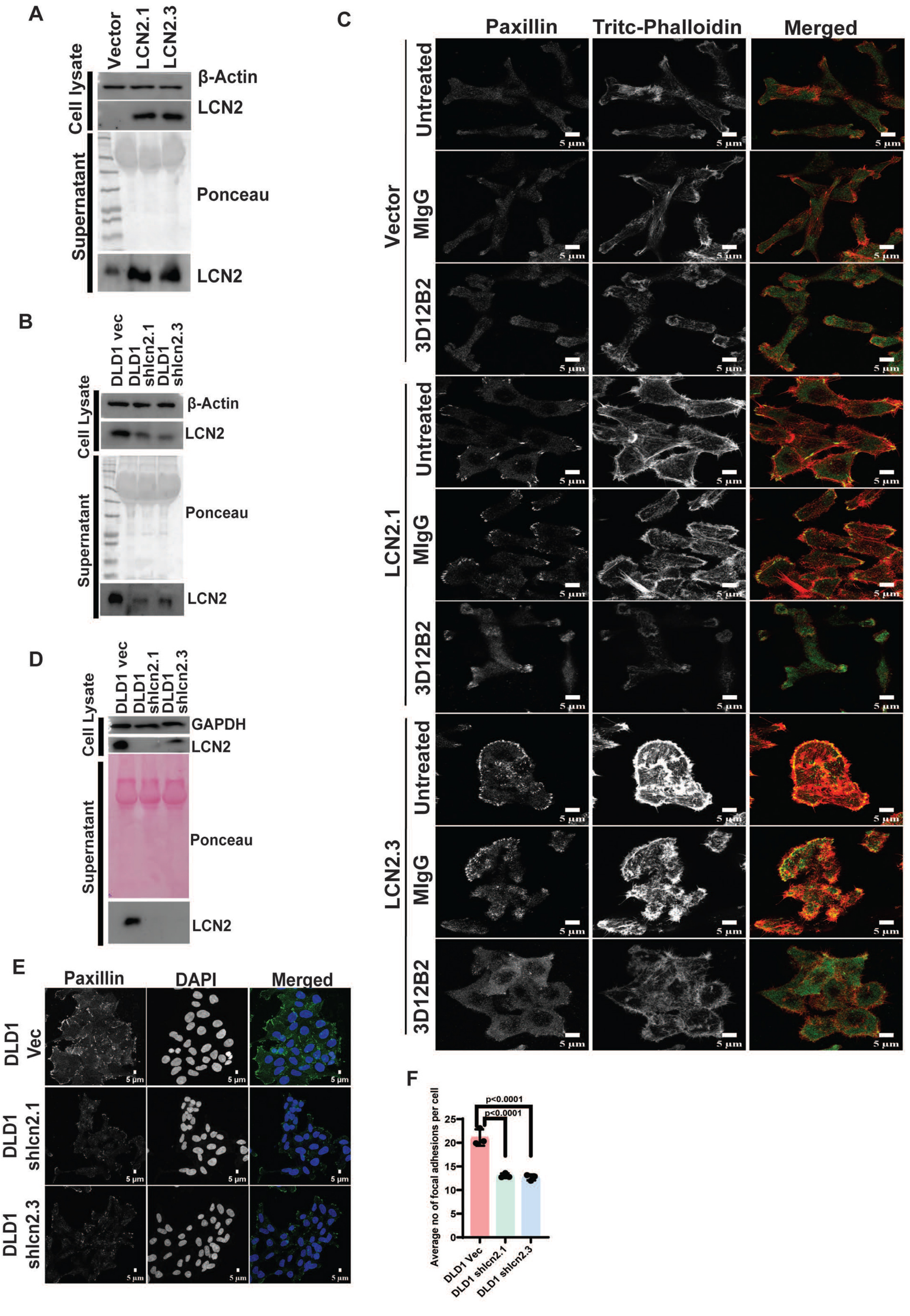

Journal of Cell Science • Supplementary information

Fig. S1. LCN2 stimulates focal adhesion formation. A-B. Protein extracts prepared from the HCT116 derived vector control (vector) and LCN2 over-expressing lines (LCN2.1 and LCN2.3) and the DLD1 derived vector control (vector) or LCN2 knockdown lines (shlcn2.1 and shlcn2.3) were resolved on SDS-PAGE gels followed by Western blotting with the indicated antibodies. Blots for actin and the ponceau stain serve as loading controls for the cell lysate and secreted proteins respectively. **C.** The indicated cell lines were untreated (UT) or treated with a non-specific mouse IgG (MIgG) or the anti-LCN2 antibody (3D12B2) and stained with antibodies to paxillin (green) and TRITC-phalloidin (red). D-F. DLD1 cells were transfected with either the vector control or two different LCN2 knockdown constructs (shlcn2.1 and shlcn2.3) and protein extracts prepared from these cells were resolved on SDS-PAGE gels followed by Western blotting with the indicated antibodies. Blots for actin and the ponceau stain serve as loading controls for the cell lysate and secreted proteins respectively. The cells were stained with antibodies to paxillin (green) or DAPI (blue)and imaged by confocal microscopy. Representative images are shown (E) and the number (F) of focal adhesions quantitated from 30 cells each in three independent experiments and the mean and standard deviation were plotted. p values were generated using a students t-test. Scale = 5µm

Journal of Cell Science • Supplementary information

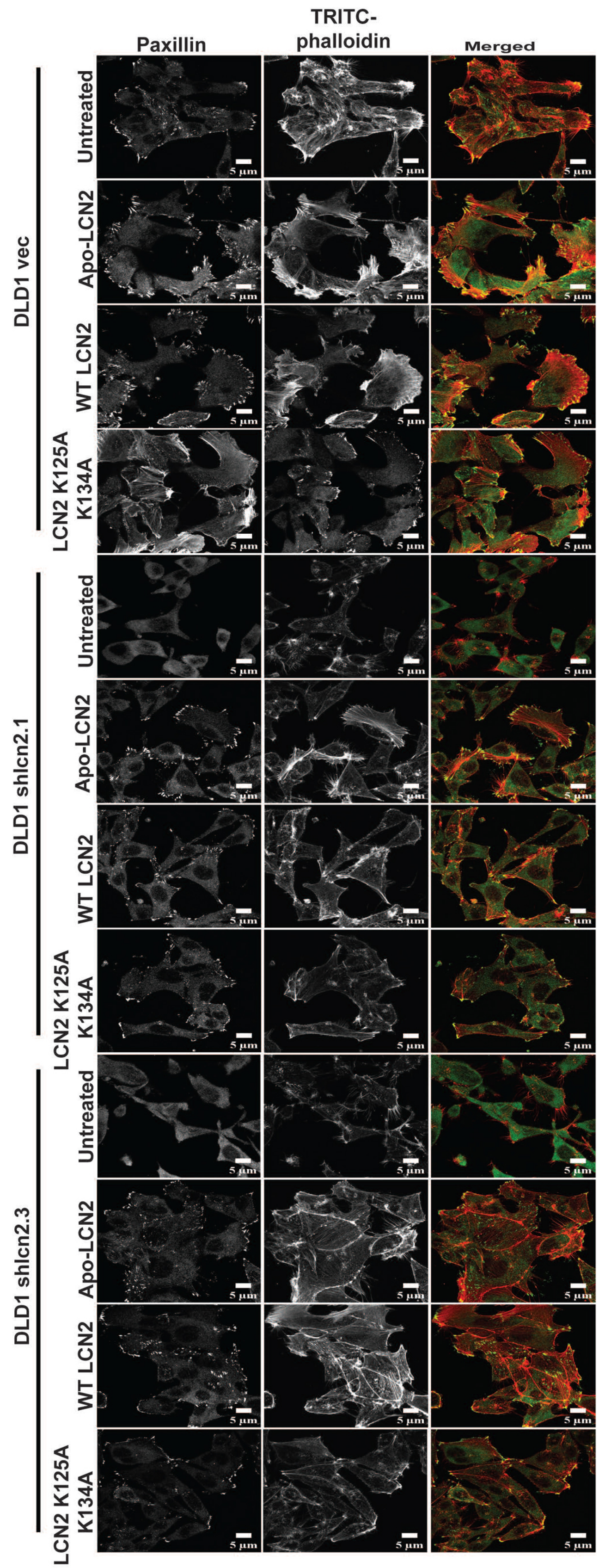

Fig. S2. The ability of LCN2 to bind iron is not required for the formation of focal adhesions. The indicated cell lines were untreated (UT) or treated with WTLCN2, ApoLCN2, or Mutant LCN2 and stained with antibodies to paxillin (green) and TRITC-phalloidin (red). Scale = 5μm

Journal of Cell Science • Supplementary information

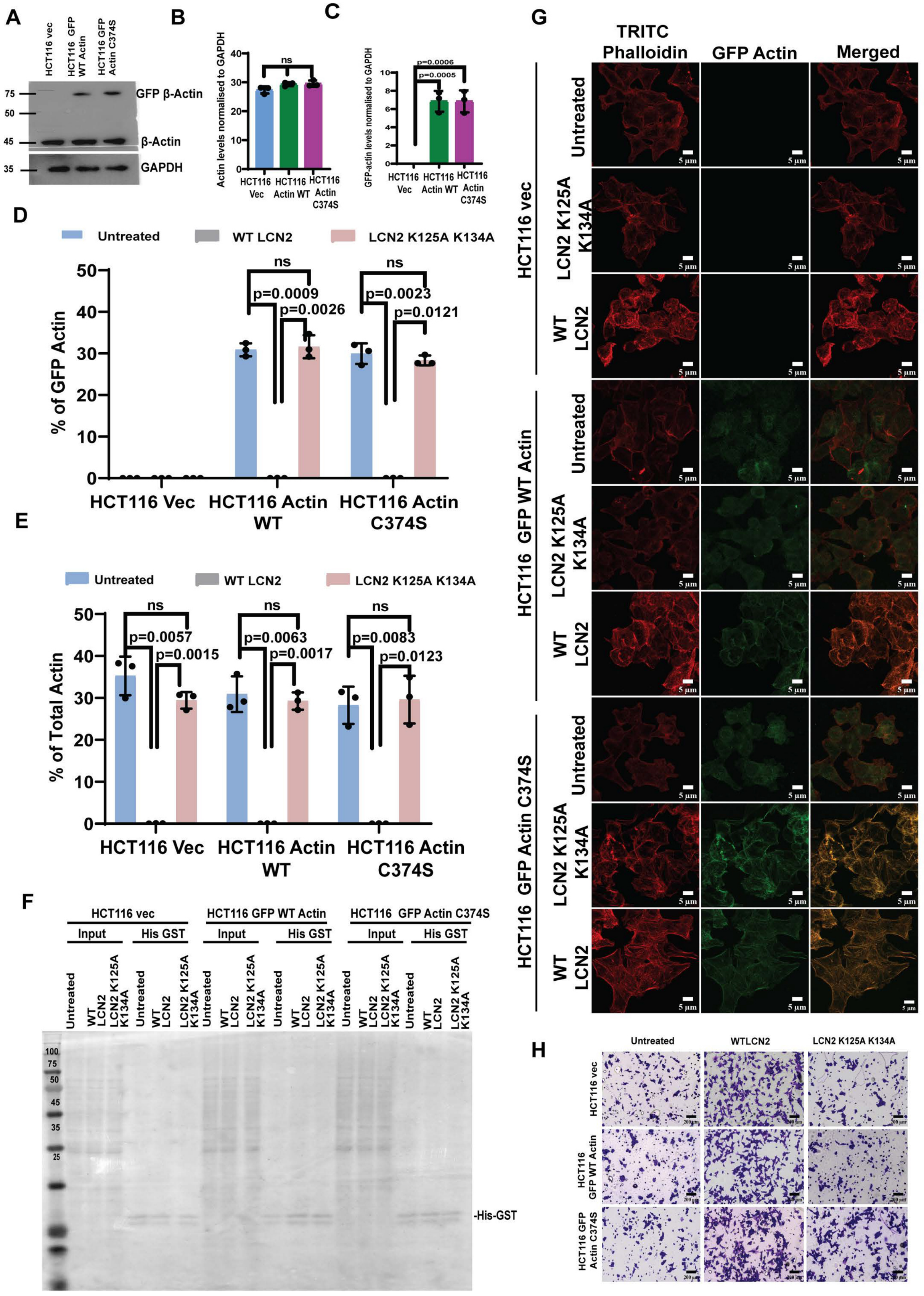

Journal of Cell Science • Supplementary information

Fig. S3. Mutation of the major glutathionylation site in actin results in a decrease in actin filament formation and invasion. A-C. Protein extracts prepared from cells transfected with either the vector control, GFP-WT Actin, or GFP Actin C374S were resolved on SDS-PAGE gels followed by Western blotting with the indicated antibodies. The quantitation for endogenous actin (B) and GFP-actin (C) are shown. The mean and standard deviation of three independent experiments is plotted. **D-E.** Quantitation of the levels of glutathionylated actin for the blots shown in figure 2D, for either GFP-actin (D) or endogenous actin (E). The mean and standard deviation of three independent experiments is plotted. **F.** Ponceau stain of the blots shown in Figure 2D. **G.** The indicated cells were treated as described and stained TRITC-phalloidin (red) and GFP-actin is in green. Scale = 5µm. Representative images are shown. **H.** The indicated cells were treated as described and used in invasion assays. Representative images are shown. Scale = 200µm. p values were generated using a students t-test.

Journal of Cell Science • Supplementary information

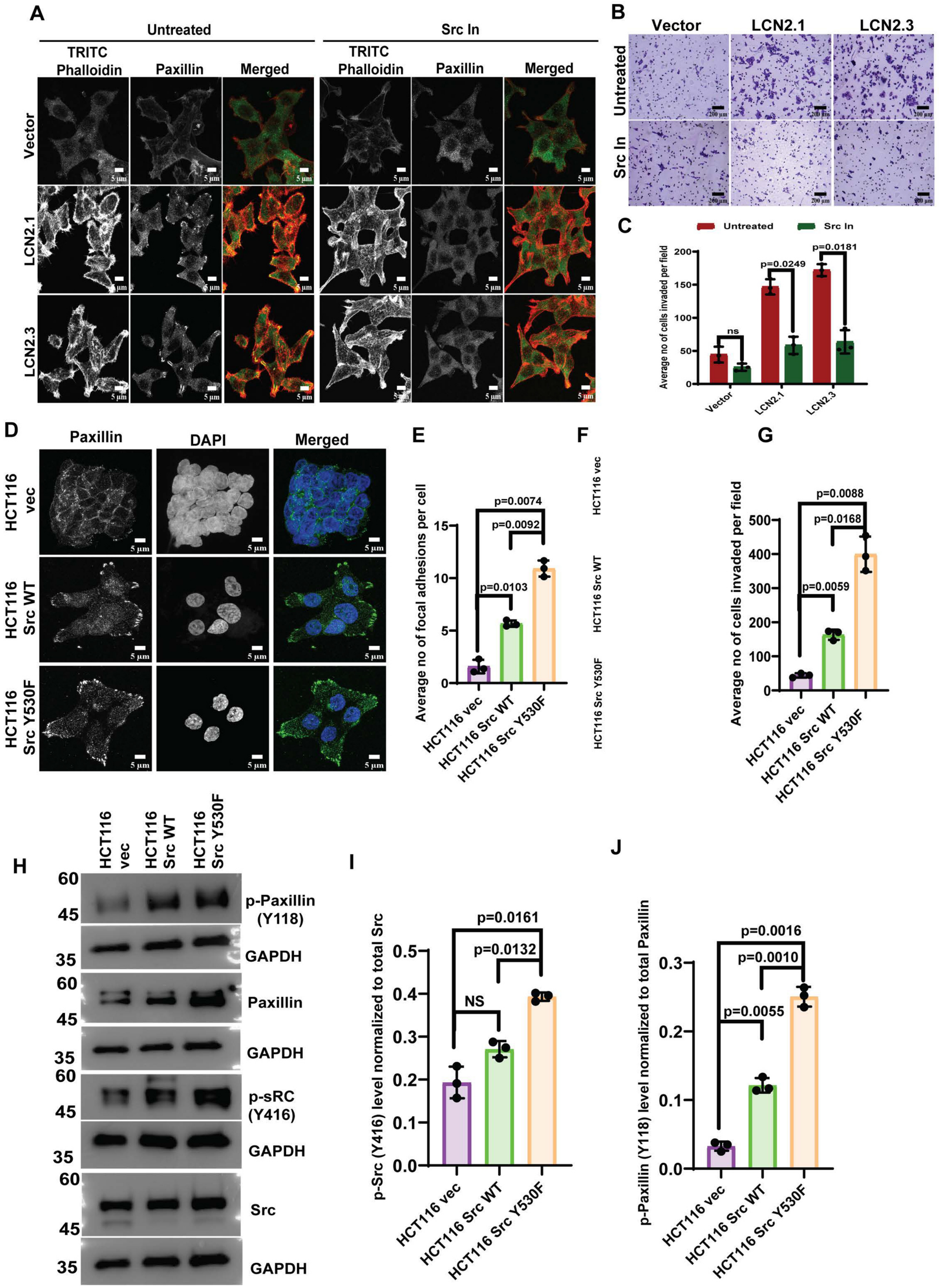

Journal of Cell Science • Supplementary information

Fig. S4. Treatment of cells with the c-Src inhibitor results in a decrease in filamentous actin formation and invasion. A-C. The indicated cell lines were untreated or treated with an inhibitor of c-Src. The treated cells were stained with antibodies to paxillin (green) and TRITC-phalloidin (red). Representative images are shown (A). The treated cells were used in invasion assays and representative images are shown (B). The mean and standard deviation were plotted for three independent experiments (C). D-J. HCT116 cells were transfected with the vector control, WT-c-SRC and a constitutively active c-Src (Y530F). The transfected cells were stained with antibodies to paxillin (green) and DAPI (blue). Representative images are shown (D) and the mean and standard deviation of number of focal adhesions per cell from 30 cells each in three independent experiments is plotted (E). Invasion assays were performed with the same cells. Representative images are shown (F) and the mean and standard deviation from three independent experiments is plotted (G). Protein extracts were prepared from the transfected cells and resolved on SDS-PAGE gels followed by Western blots with the indicated antibodies (H). The levels of phosphorylated c-Src (Y416) (I) and phosphorylated paxillin (Y118) (J) were plotted. Where indicated, p values were determined using a student's t-test. Scale = 5μm for fluorescence images and Scale = 200μm for invasion assays.

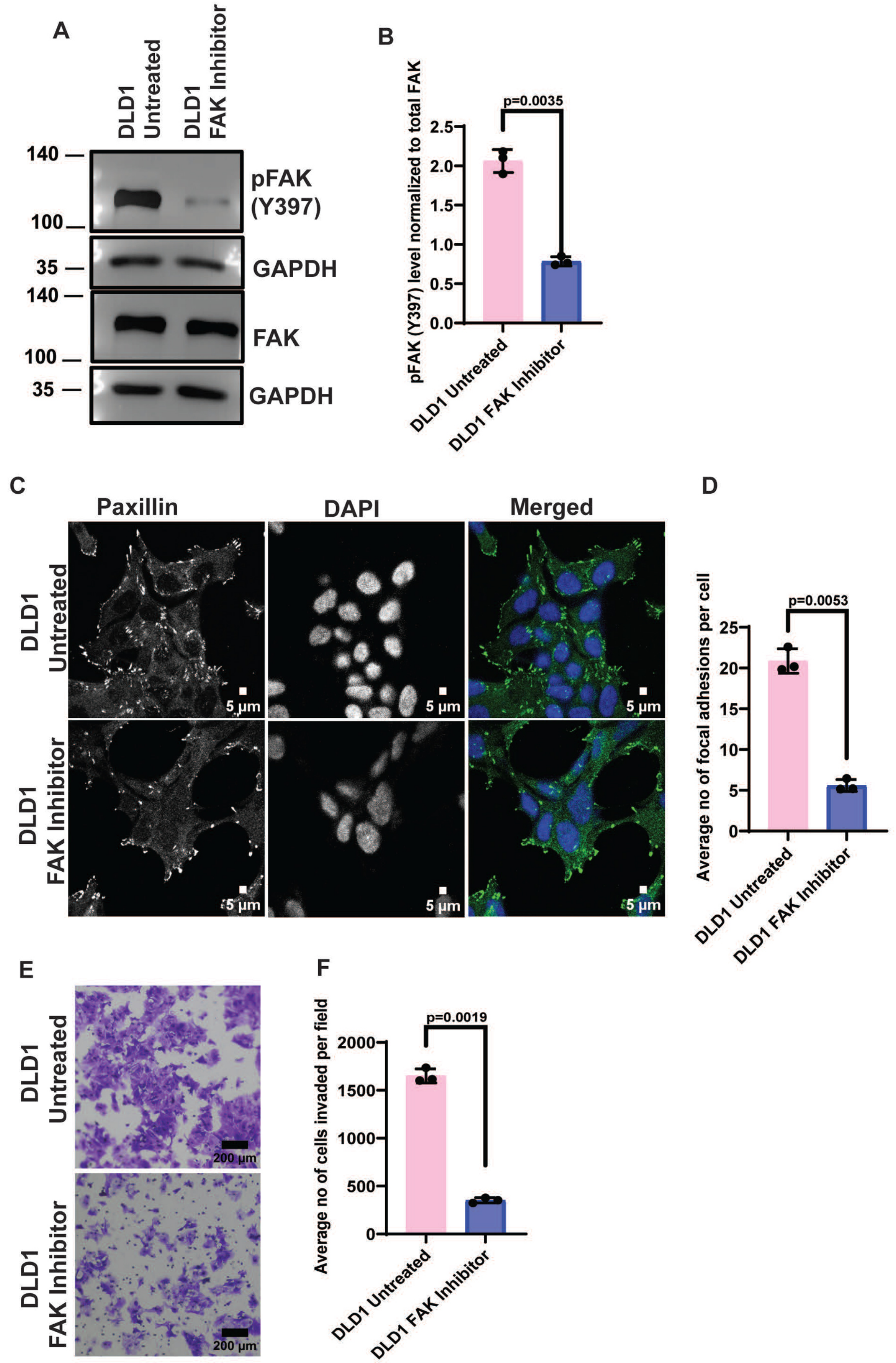

Journal of Cell Science • Supplementary information

Fig. S5. Inhibition of FAK leads to a decrease in focal adhesion formation and invasion. A-F. DLD1 cells were either untreated or treated with the FAK inhibitor as described. Protein extracts from these cells were resolved on SDS-PAGE gels and Western blots performed with the indicated antibodies (A) and the levels of p-FAK (Y397) were quantitated and the mean and standard deviation of three independent experiments is plotted (B). The cells were stained with antibodies to paxillin (green) and DAPI (blue). Representative images are shown (C) and the mean and standard deviation of number of focal adhesions per cell from 30 cells each in three independent experiments is plotted (D). Invasion assays were performed with the same cells. Representative images are shown (E) and the mean and standard deviation from three independent experiments is plotted (F). Scale = 5μm for fluorescence images and Scale = 200μm for invasion assays.

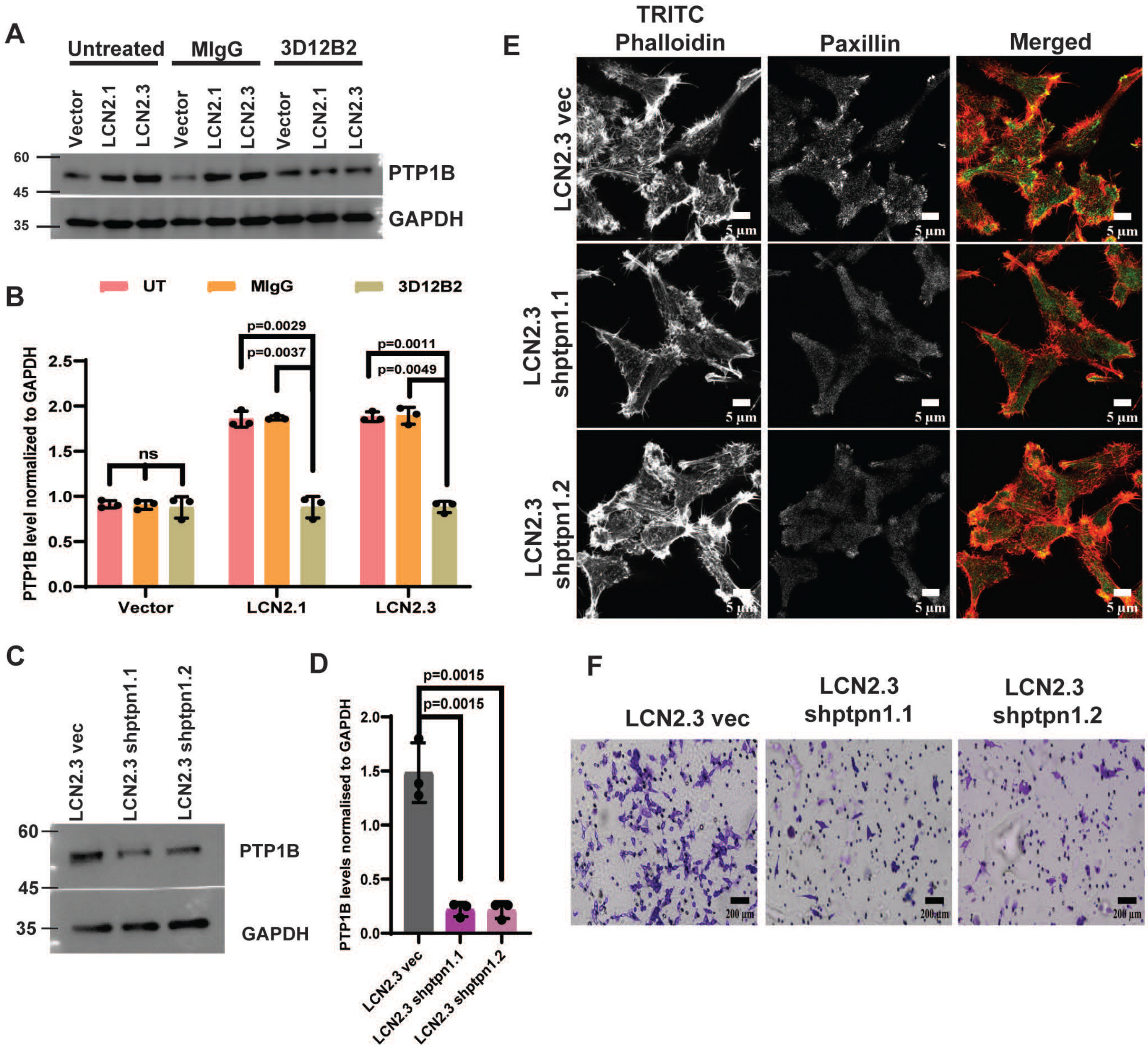

Fig. S6. LCN2 stimulates PTP1B expression to promote invasion. A-B. Protein extracts prepared from HCT116 derived vector and LCN2 over-expressing cell lines were untreated (UT) or treated with a non-specific mouse IgG (MIgG) or the anti-LCN2 antibody (3D12B2) were resolved on SDS-PAGE gels followed by Western blots and probed with the indicated antibodies (A). The mean and standard deviation from three independent experiments is plotted (B). **C-D.** Western blots of PTP1B protein extracts (C) or mRNA levels of PTPN1 (D) for the LCN2.3 derived vector control (LCN2.3vec) or PTP1B knockdown clones (LCN2.3shptpn1.1 and shptpn1.2). **E-F.** The LCN2.3 derived vector control (LCN2.3vec) or PTP1B knockdown clones (LCN2.3shptpn1.1 and shptpn1.2) were either stained with antibodies to paxillin and TRITC-phalloidin (E) or used in matrigel invasion assays (F). Representative images are shown and the scale bars are 5µm and 200µm respectively. Where indicated, p values were determined using a student's t-test.

Journal of Cell Science • Supplementary information

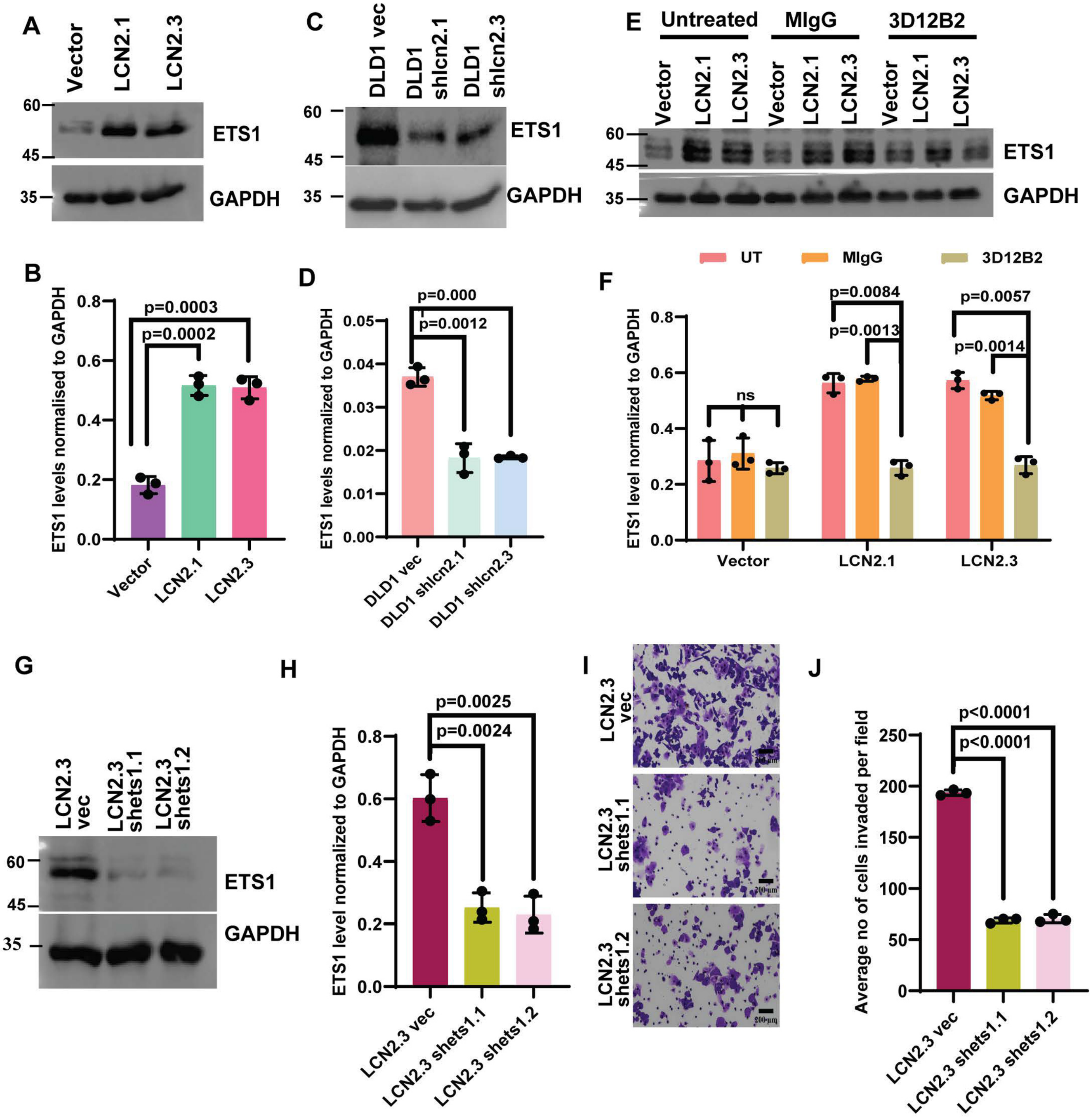

Journal of Cell Science • Supplementary information

Fig. S7. LCN2 expression leads to increased ETS1 levels which is required for invasion. A-D. Protein extracts from the HCT116 derived vector control (vector) and LCN2 over-expressing lines (LCN2.1 and LCN2.3) and the DLD1 derived vector control (vector) or LCN2 knockdown lines (shlcn2.1 and shlcn2.3) were resolved on SDS-PAGE gels followed by Western blotting with the indicated antibodies (A&C). The mean and standard deviation of three independent experiments is plotted (B&D). **E-F.** Protein extracts prepared from the indicated cell lines that were untreated (UT) or treated with a non-specific mouse IgG (MIgG) or the anti-LCN2 antibody (3D12B2) were resolved on SDS-PAGE gels followed by Western blotting with antibodies to ETS1 and GAPDH (E). ETS1 levels normalized to GAPDH were measured in three independent experiments and the mean and standard deviation were plotted (F). **G-H.** Protein extracts prepared from the LCN2.3 derived vector control (LCN2.3vec) or ETS1 knockdown clones (LCN2.3ets1.1 and shets1.2) were resolved on SDS-PAGE gels followed by Western blotting with the indicated antibodies (G) and the mean and standard deviation of ETS1 levels in three independent experiments were plotted. **I-J.** The LCN2.3 derived vector control (LCN2.3vec) or ETS1 knockdown clones (LCN2.3ets1.1 and shets1.2) were used in invasion assays. Representative images are shown (Scale = 200µm) and the mean and standard deviation of invading cells from three independent experiments is plotted. Where indicated, p values were determined using a student's t-test.

Journal of Cell Science • Supplementary information

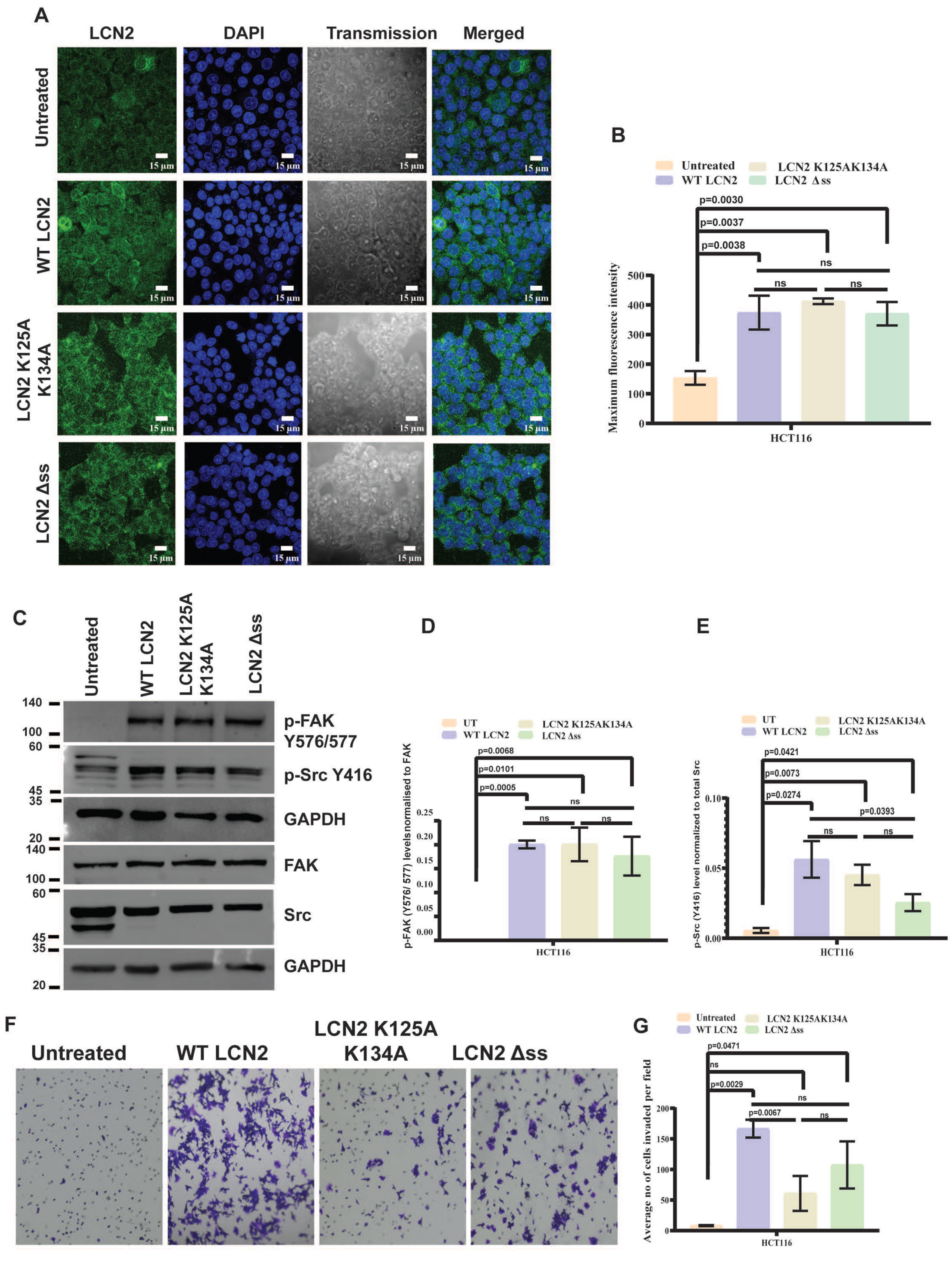

Journal of Cell Science • Supplementary information

Fig. S8. A secretion-defective mutant of LCN2 promotes the phosphorylation of FAK and c-Src. A-G. HCT116 cells were treated with the vehicle control (untreated) or with the indicated recombinant proteins (WT LCN2, K125AK134A and LCN2Δss). The cells were fixed and stained with antibodies to LCN2 (green) and counterstained with DAPI (blue). Representative images are shown, and the scale bar = 15μm (A). The fluorescence intensity was determined in 30 cells in three independent experiments, and the mean and standard deviation were plotted (B). Protein extracts prepared from the cells were resolved on SDS-PAGE gels followed by Western blotting with the indicated antibodies (C). GAPDH served as a loading control. The levels of pFAK576/5777 (D) and pSrcY416 (E) were determined in three independent experiments and the mean and standard deviation are plotted. $2x10^5$ of the treated cells were used to perform Matrigel invasion assays. Representative images are shown (F), and the mean and standard deviation of three independent experiments are plotted (G). p values were generated using a student t-test.

Image in the manuscript

Figure 2 D

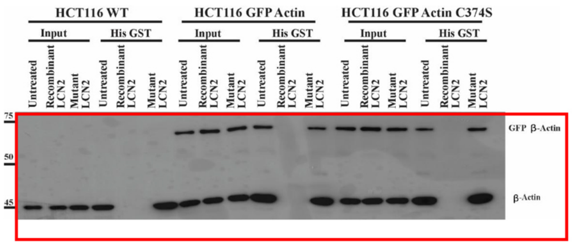

Set1

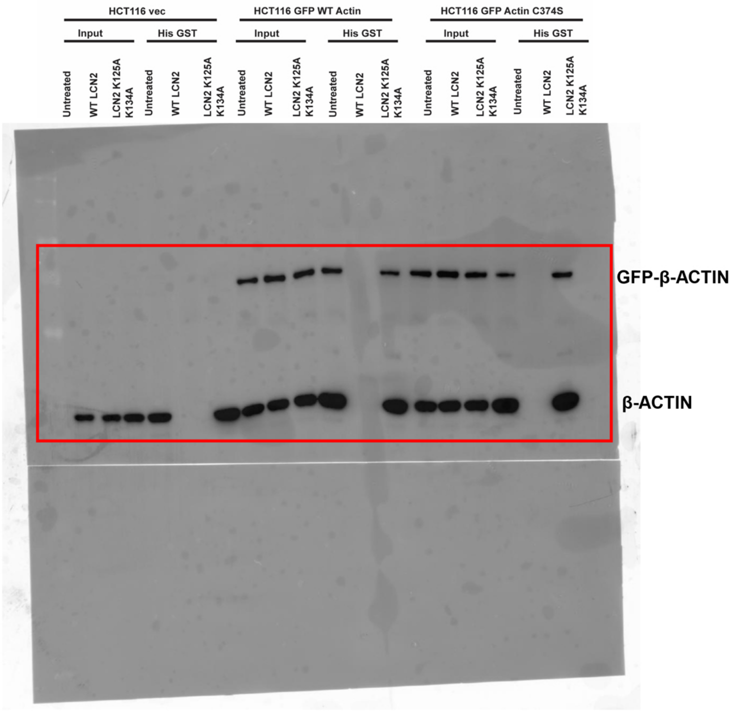

FIGURE 2D
SET-2

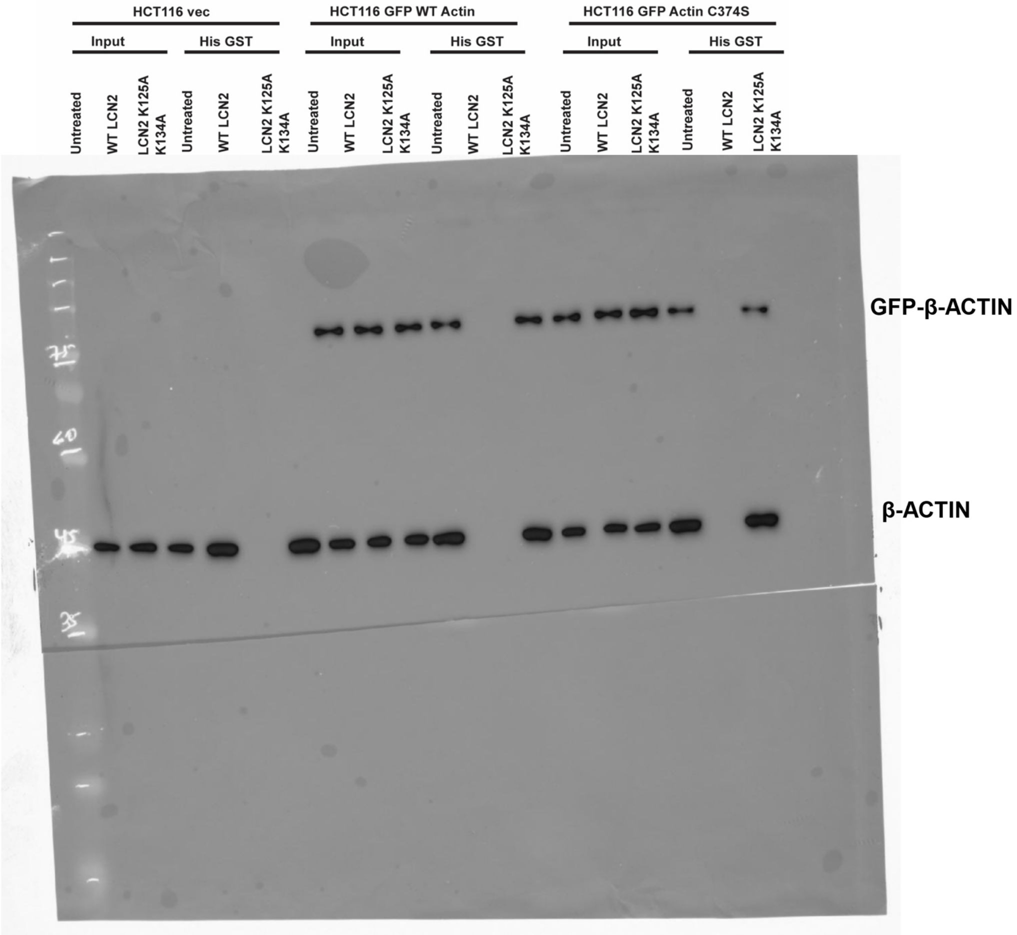

Journal of Cell Science • Supplementary information

FIGURE 2D
SET-3

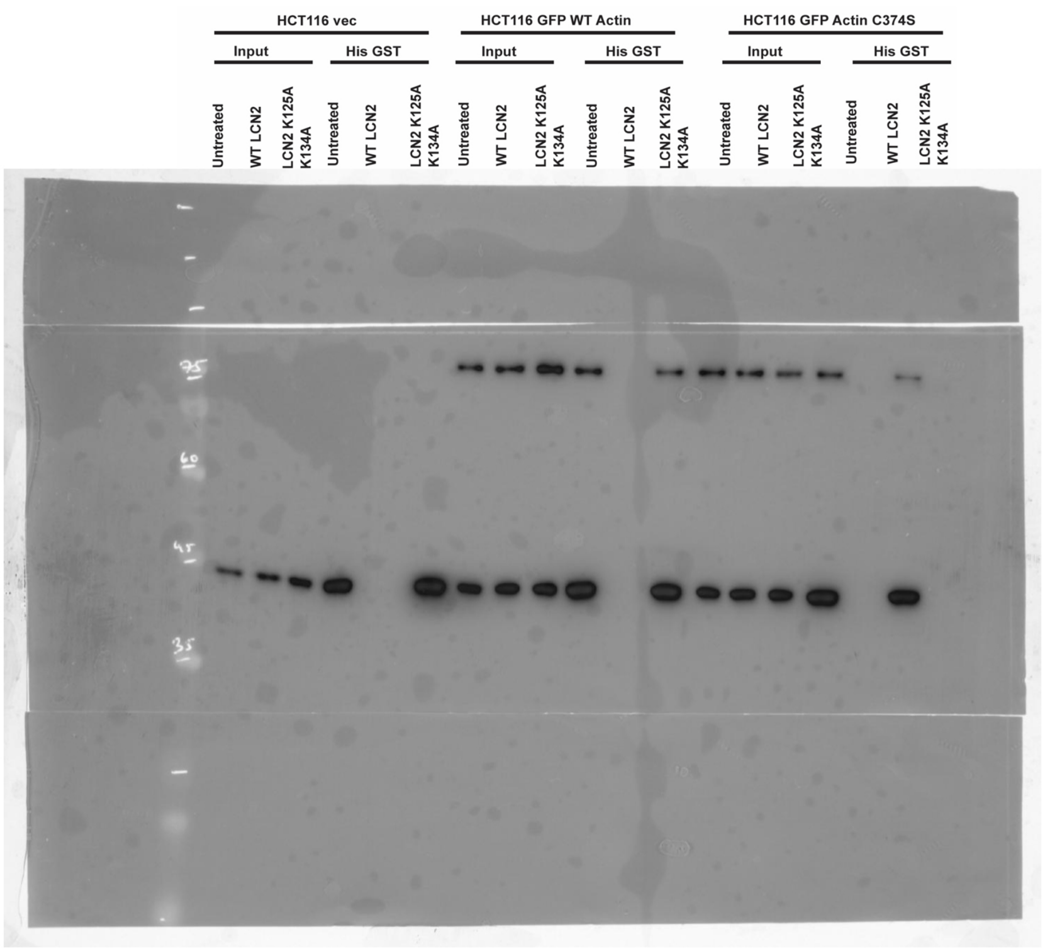

Image in the manuscript

Figure 3A

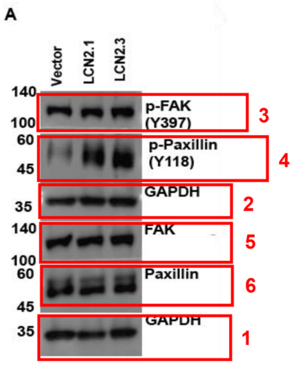

Figure 3A
Set1 (used in figure panel)
Set2

Entire blot

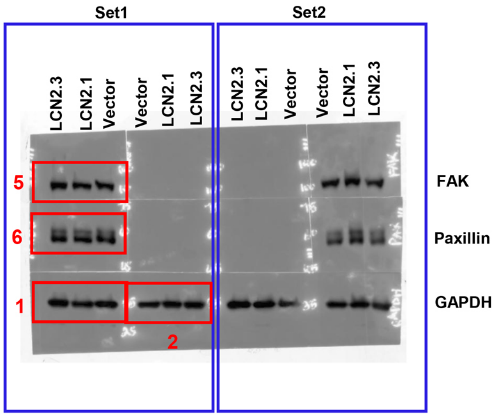

Developed separately

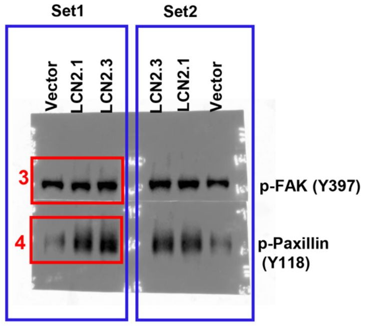

Journal of Cell Science • Supplementary information

Figure 3A
Set3
Set4

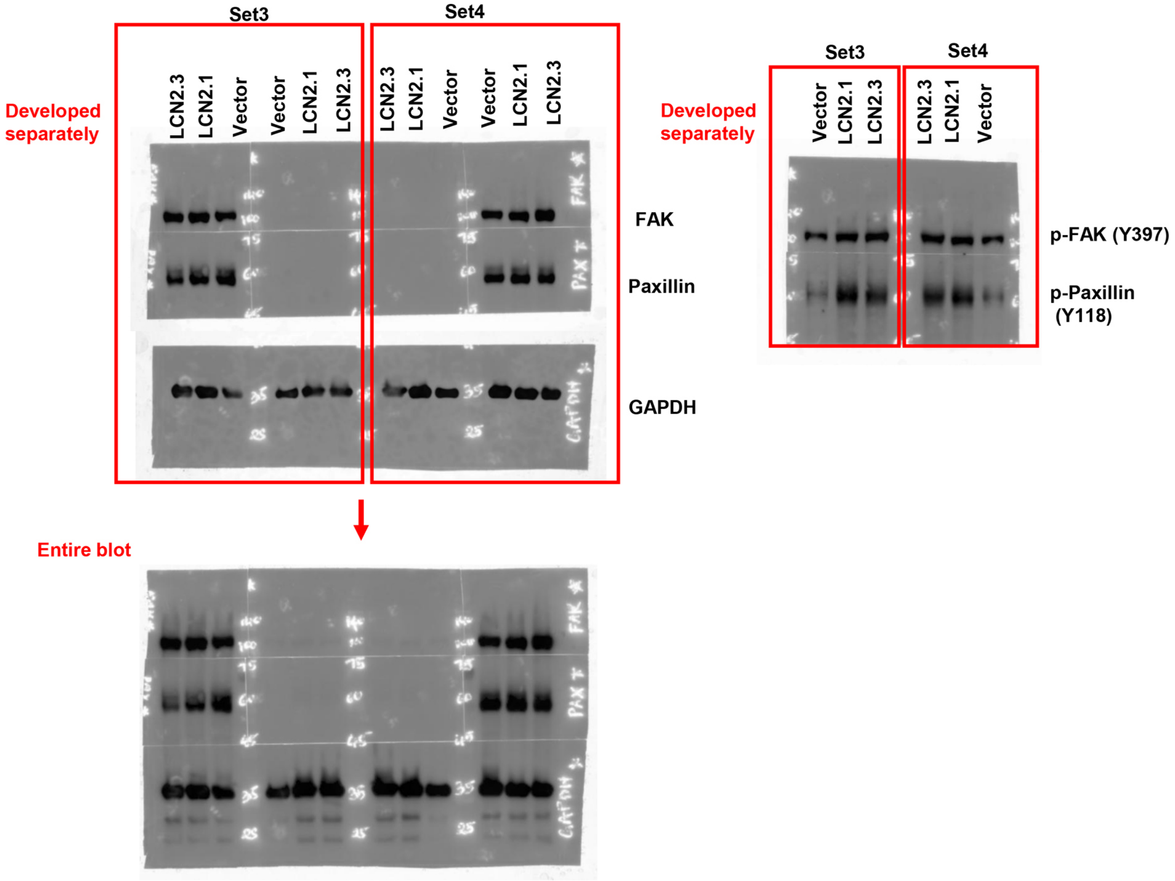

Image in the manuscript
Figure 3D

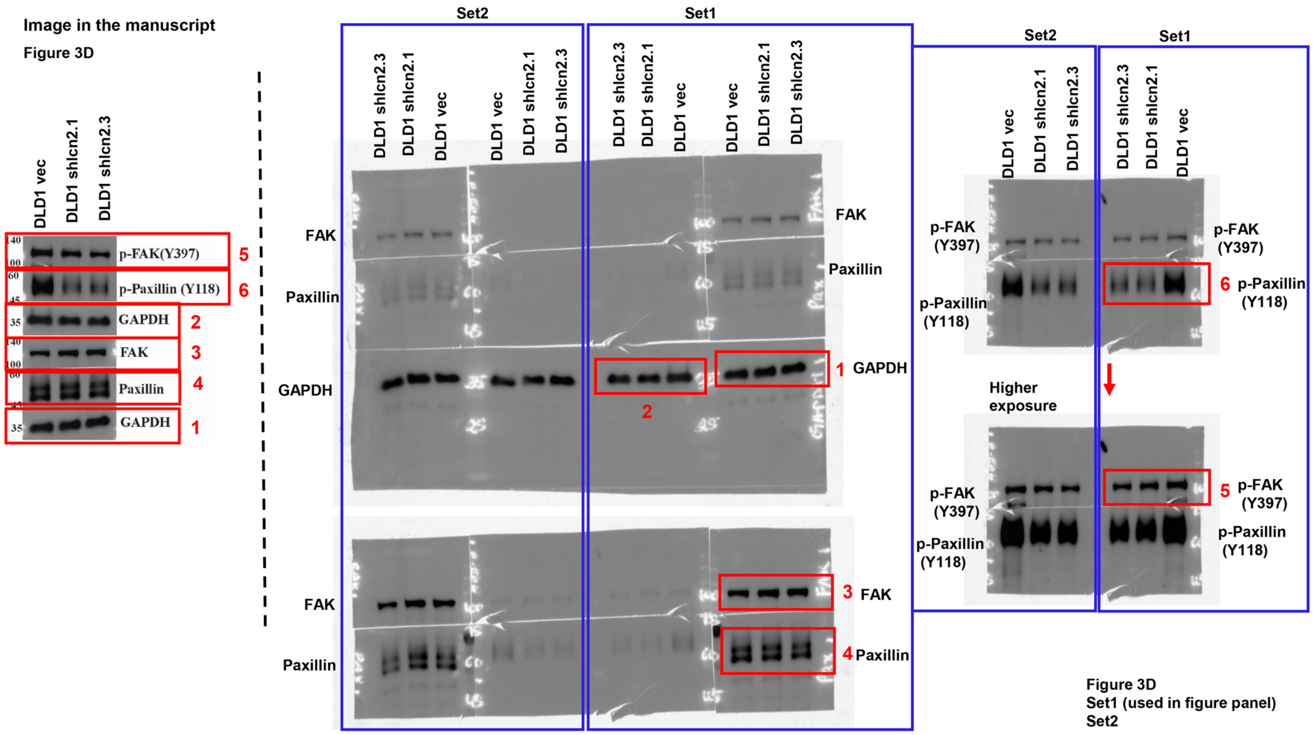

Figure 3D
Set1 (used in figure panel)
Set2

Journal of Cell Science • Supplementary information

Figure 3D

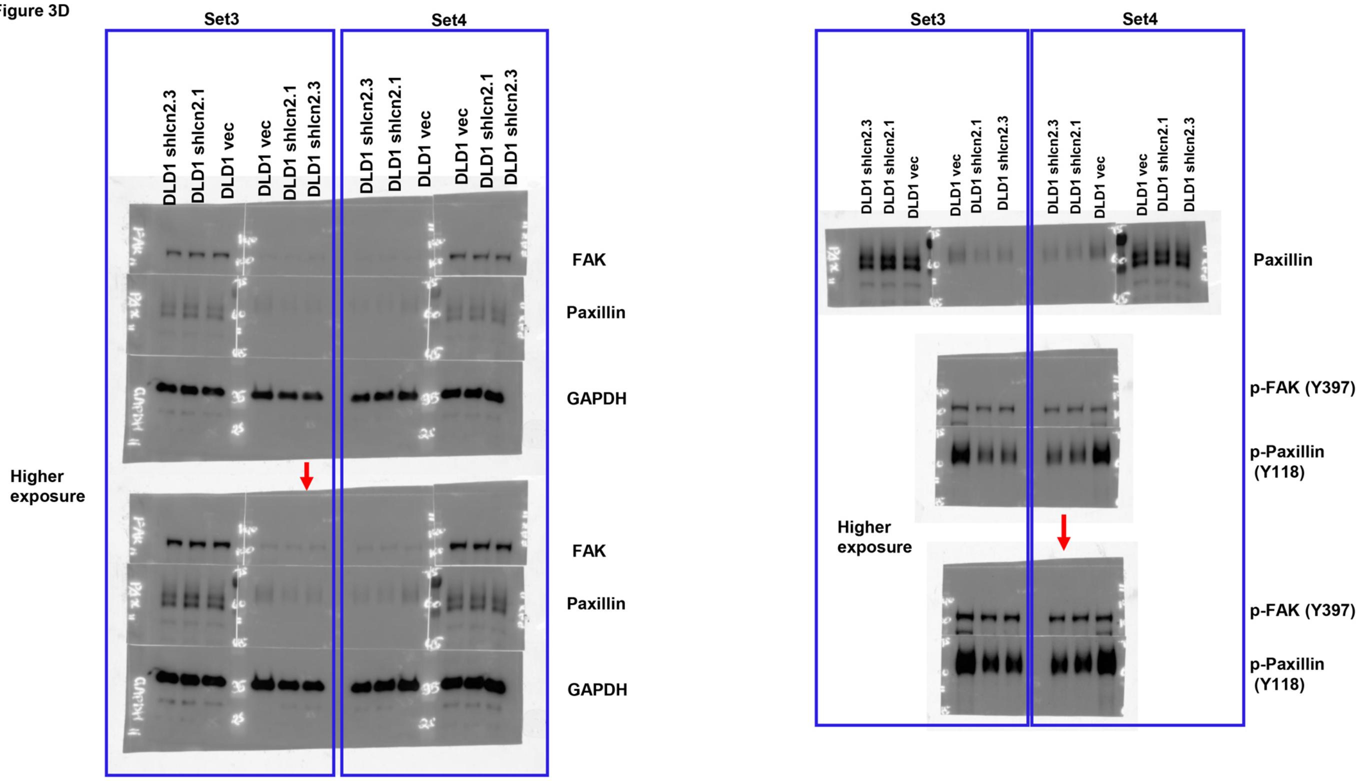

Image in the manuscript

Figure 3G

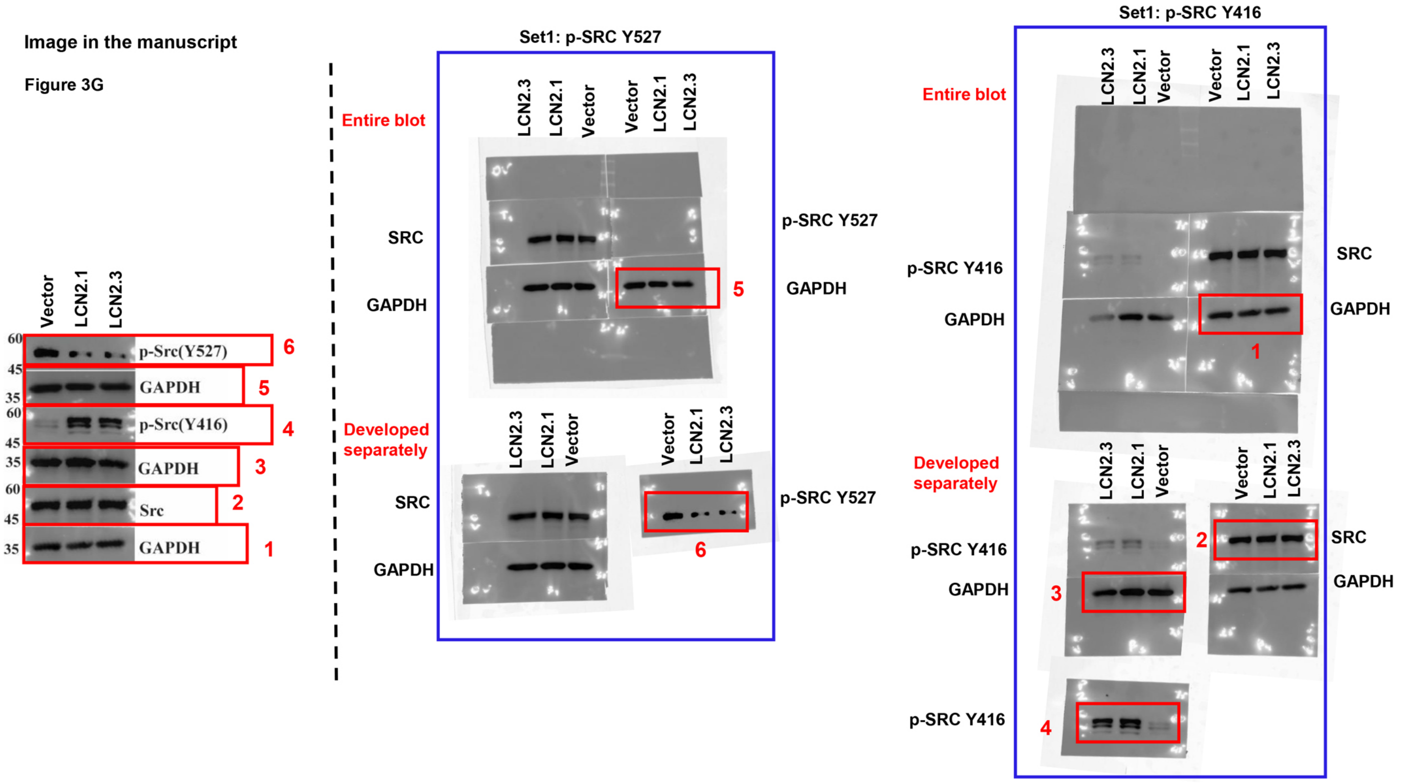

Journal of Cell Science • Supplementary information

Figure 3G

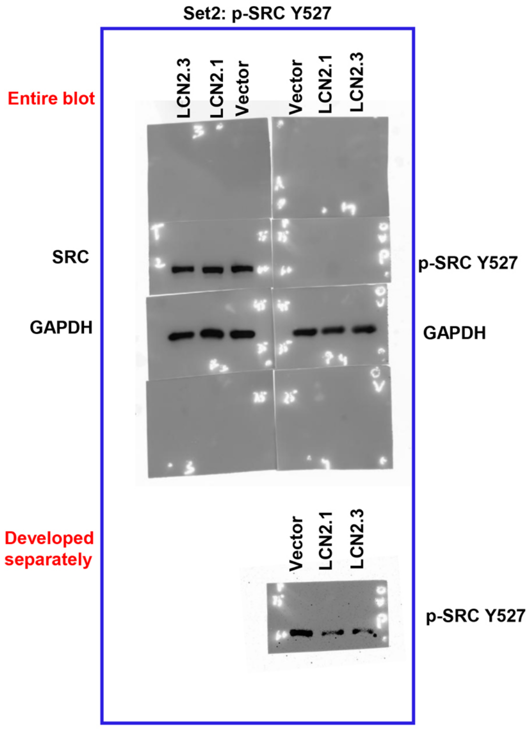

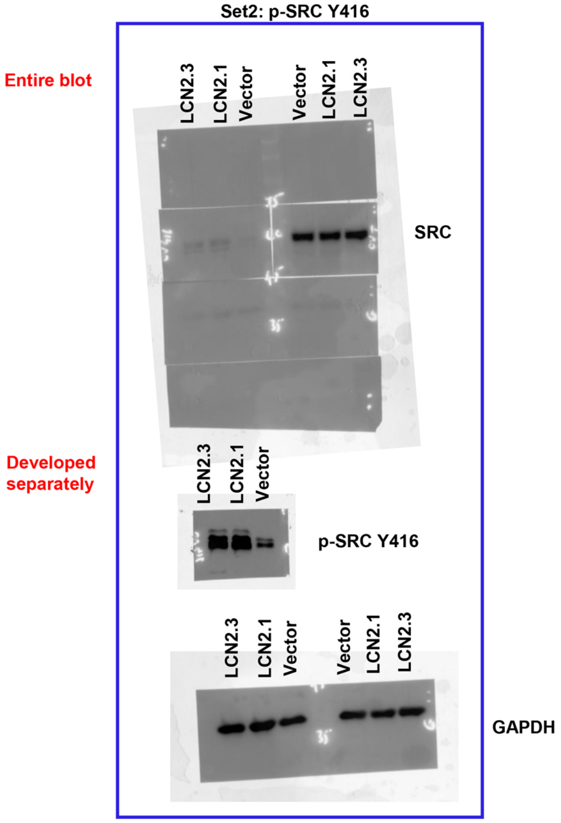

Figure 3G

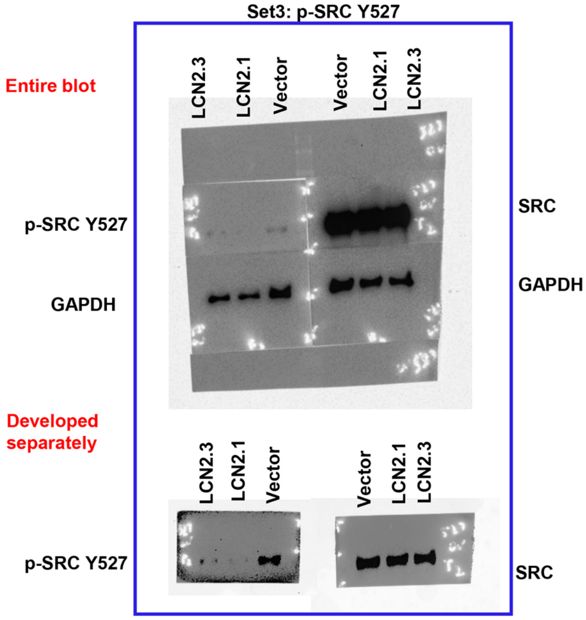

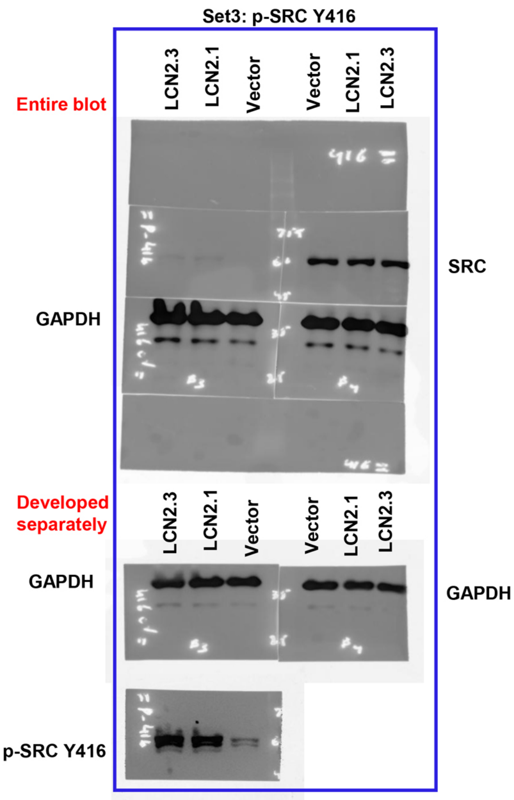

Journal of Cell Science • Supplementary information

Image in the manuscript

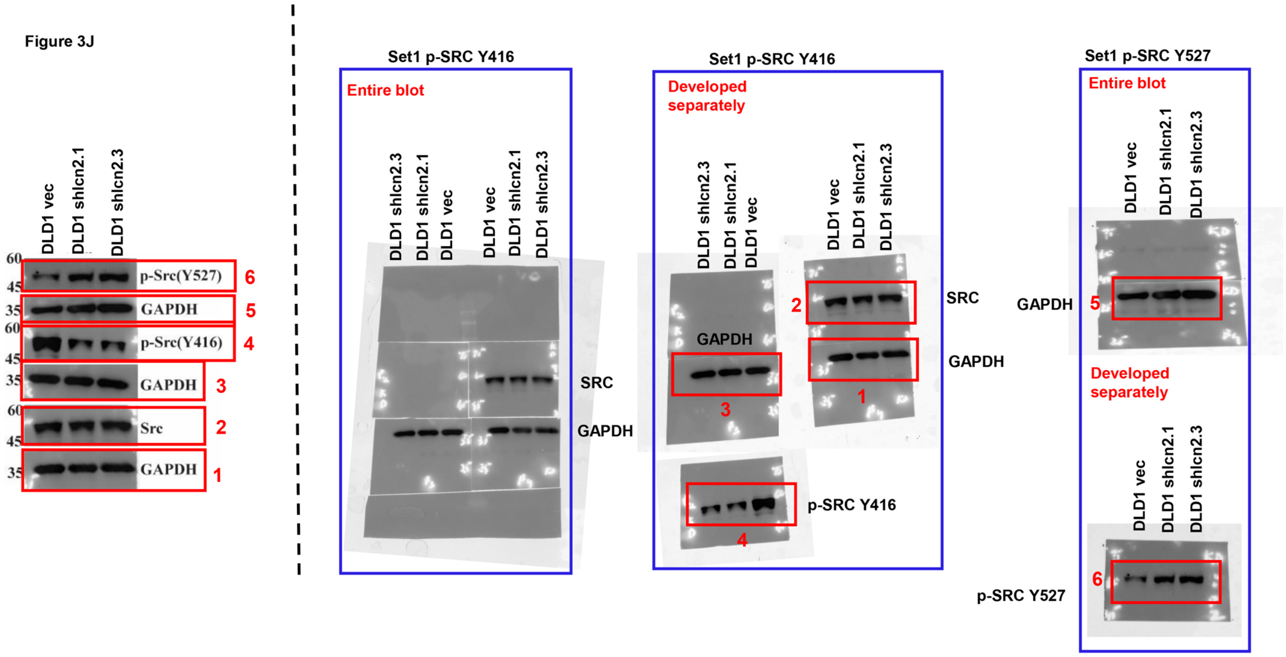

Figure 3J

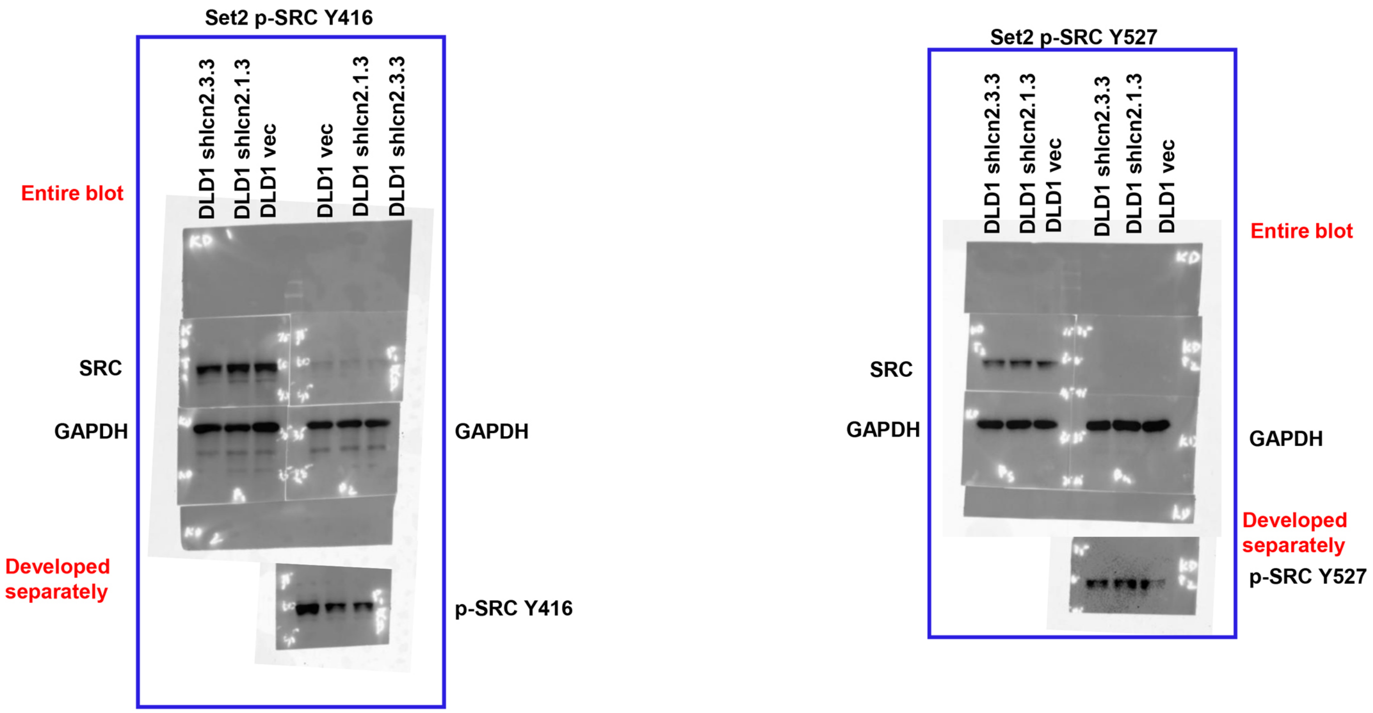

Journal of Cell Science • Supplementary information

Figure 3J

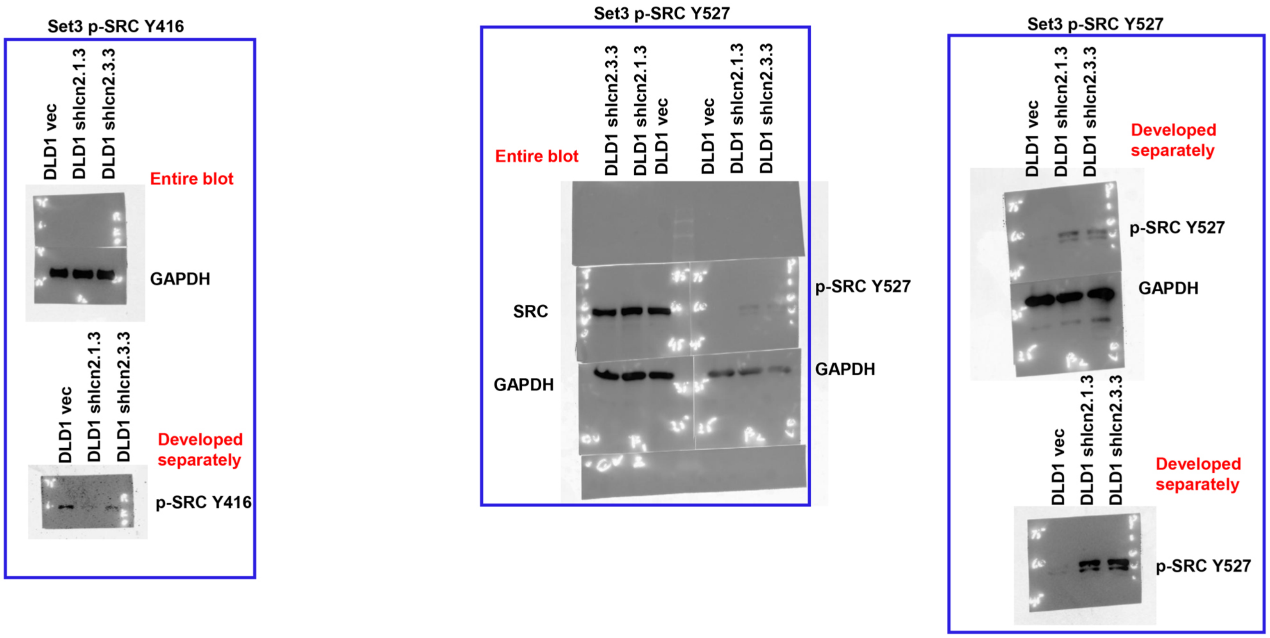

Image in the manuscript

Figure 4A

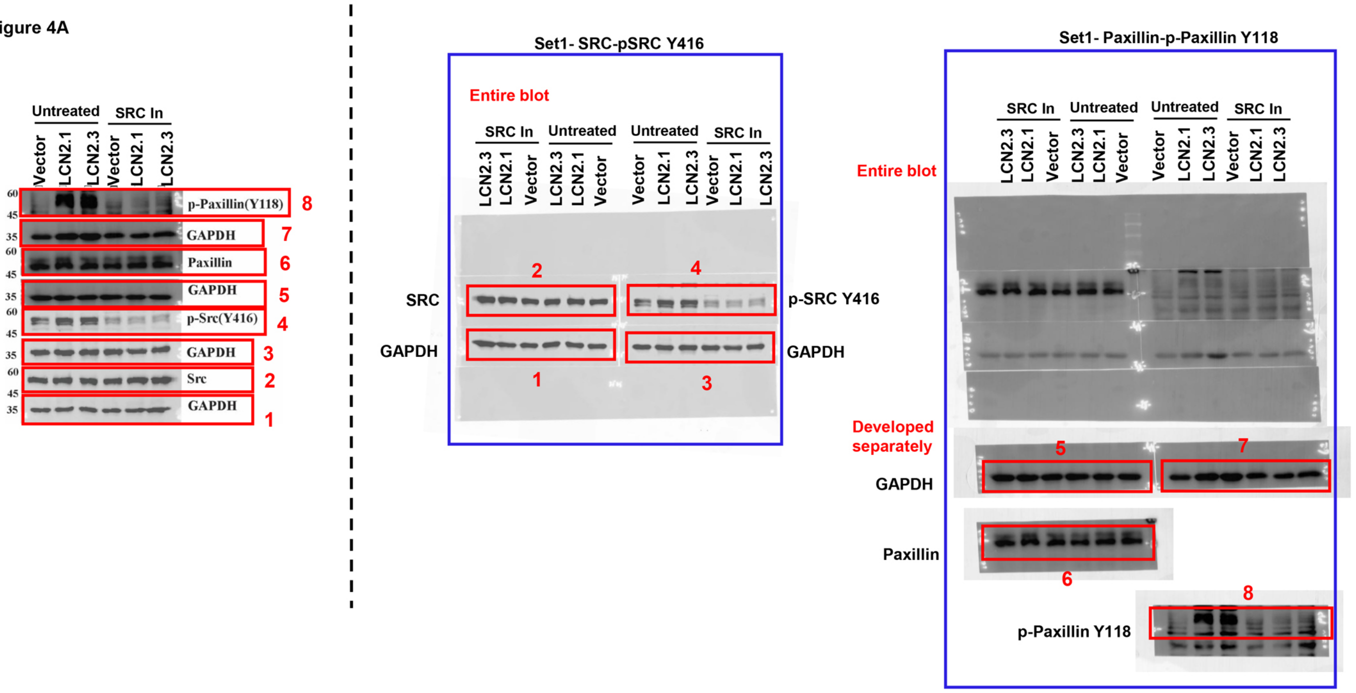

Journal of Cell Science • Supplementary information

Figure 4A

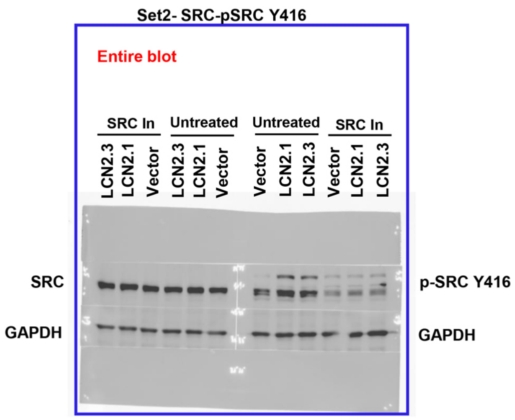

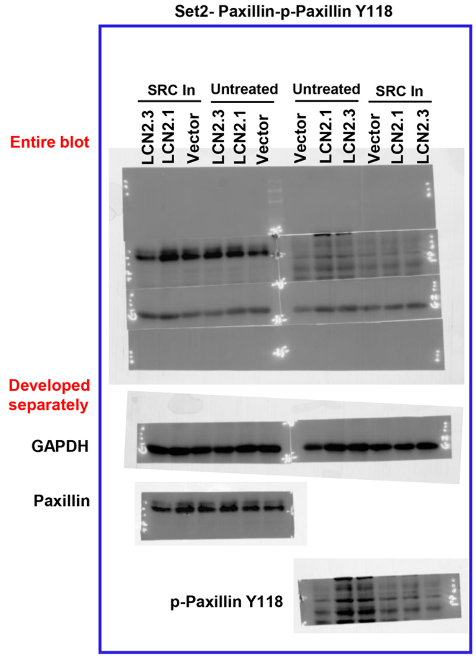

Figure 4A

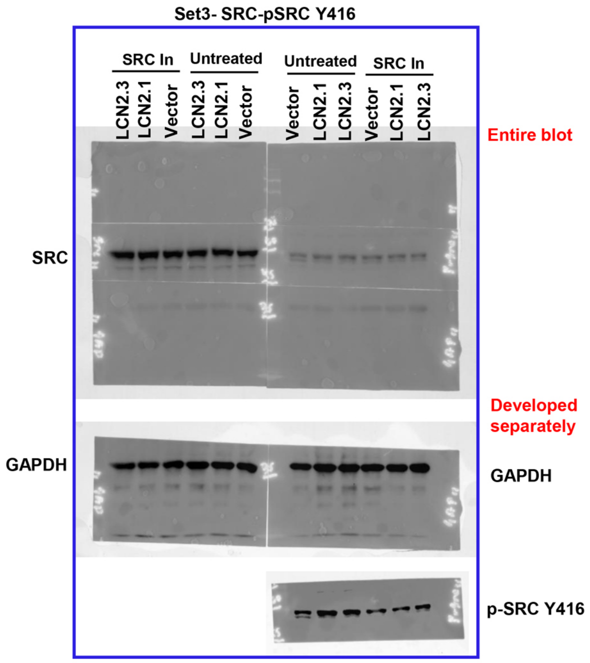

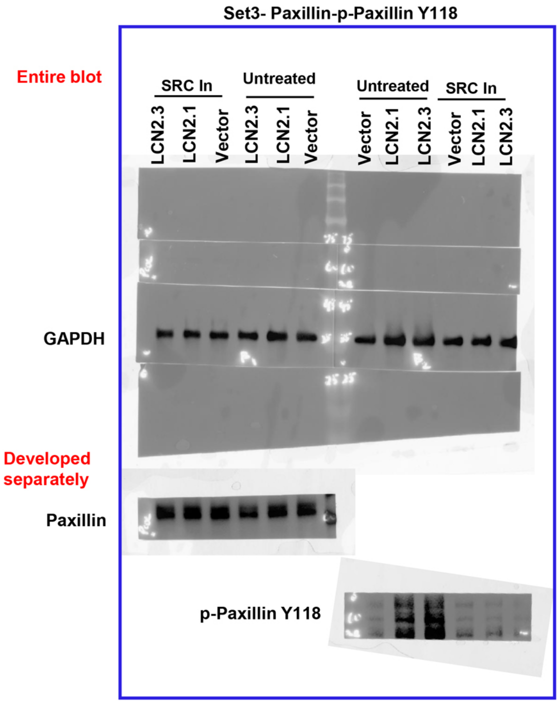

Image in the manuscript

Figure 4A

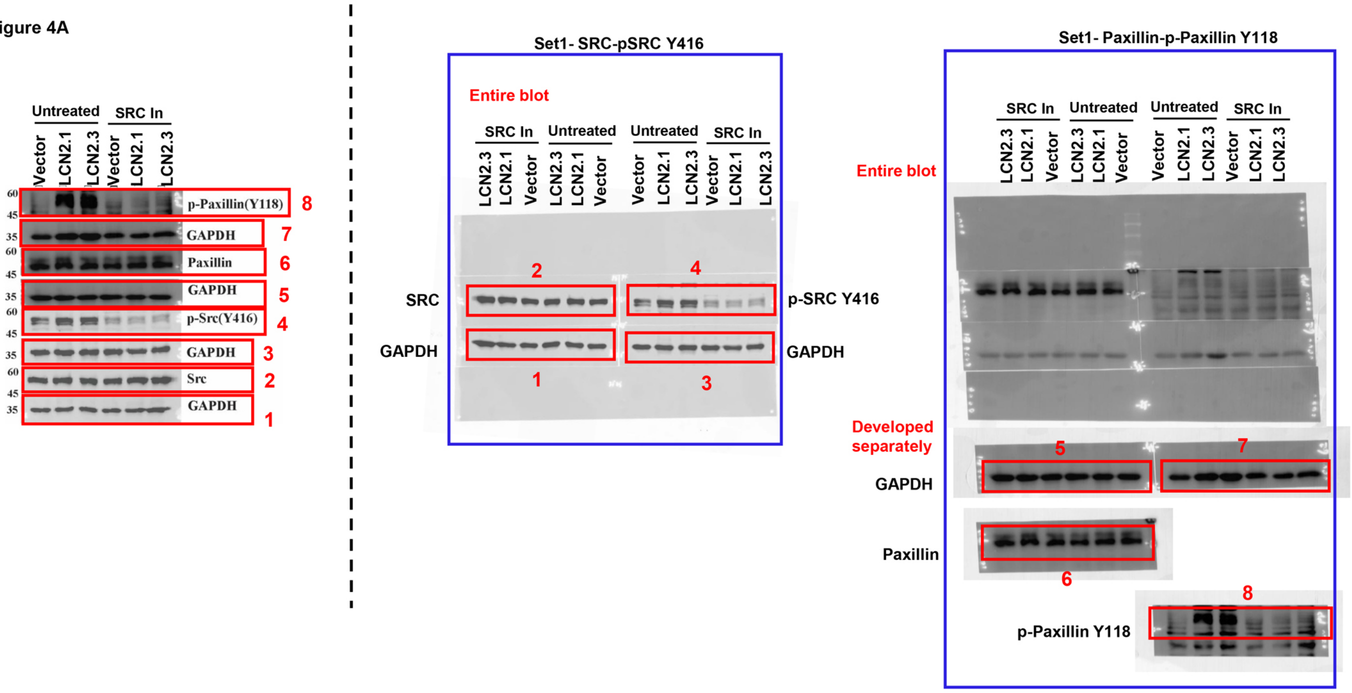

Figure 4A

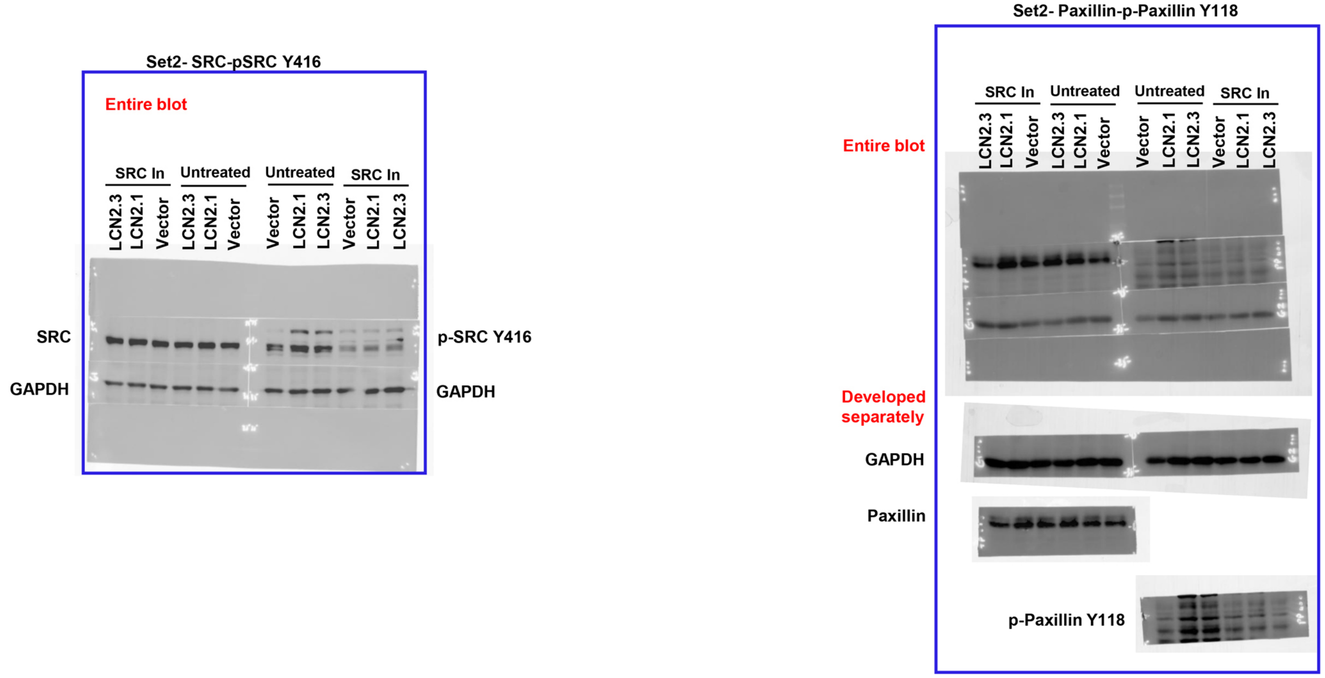

Journal of Cell Science • Supplementary information

Figure 4A

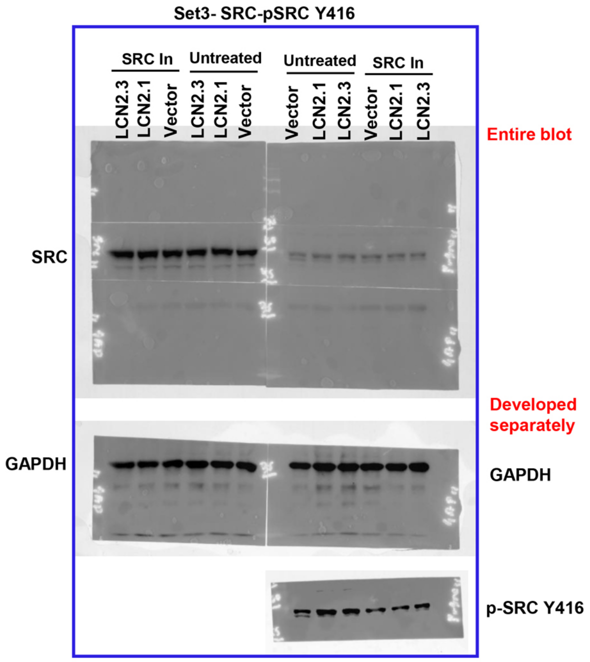

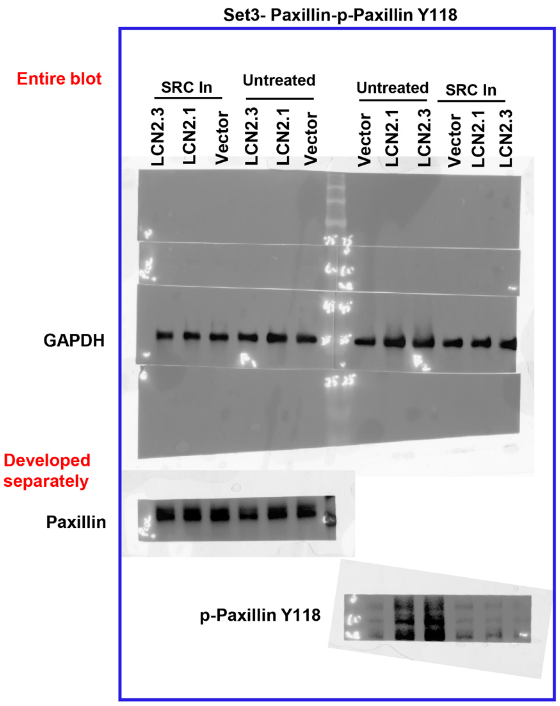

Image in the manuscript

Figure 4 G

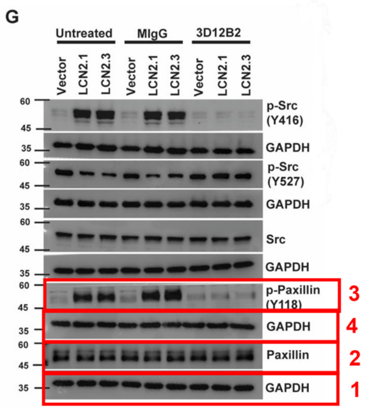

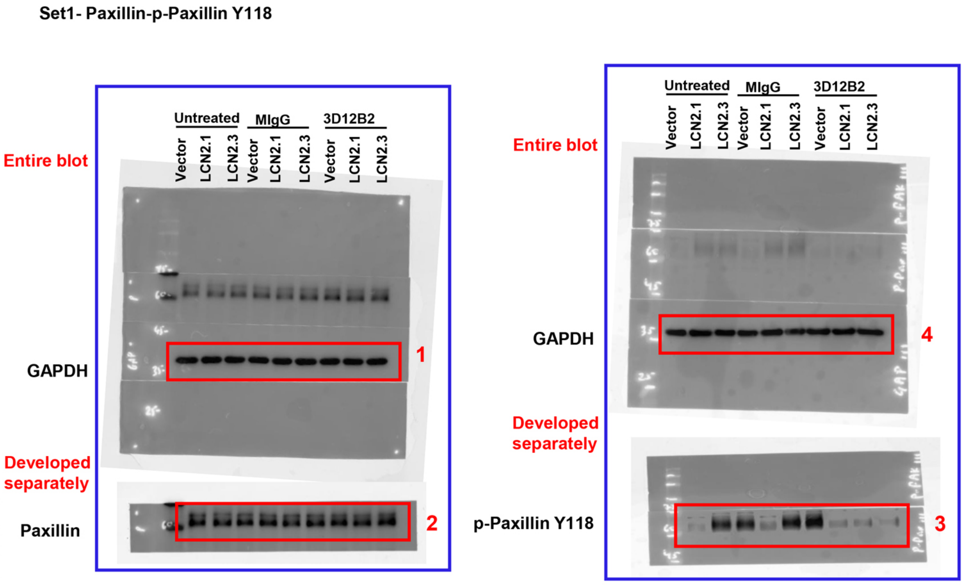

Journal of Cell Science • Supplementary information

Figure 4 G

Set2- Paxillin-p-Paxillin Y118

Entire blot

Paxillin

GAPDH

Developed separately

GAPDH

Paxillin

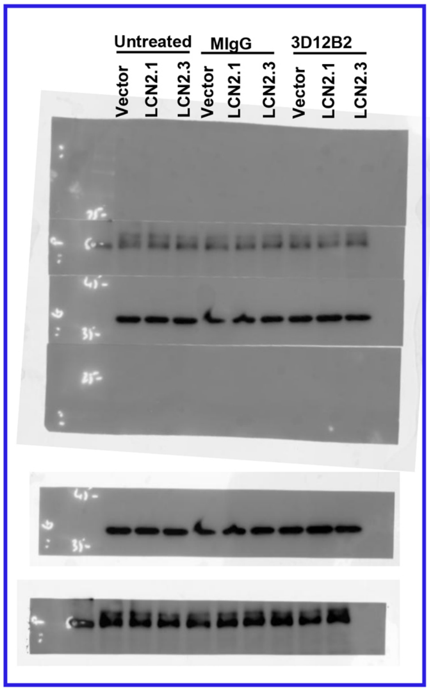

Entire blot

GAPDH

Developed separately

p-Paxillin Y118

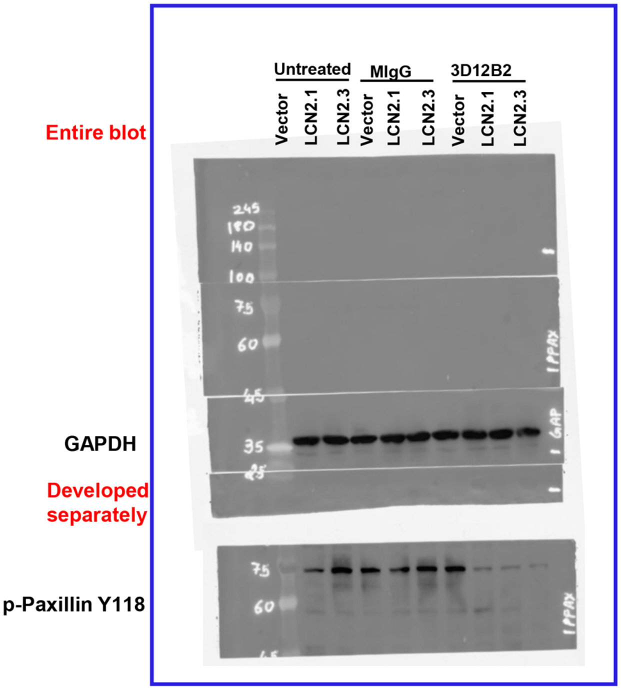

Figure 4 G

Set3- Paxillin-p-Paxillin Y118

Entire blot

Paxillin

GAPDH

Developed separately

Paxillin

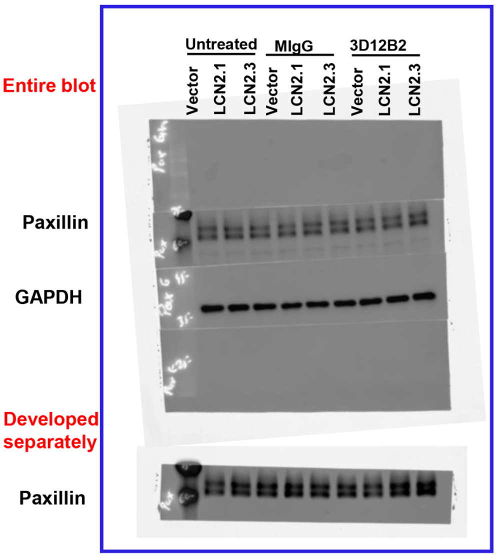

Entire blot

GAPDH

Developed separately

GAPDH

p-Paxillin Y118

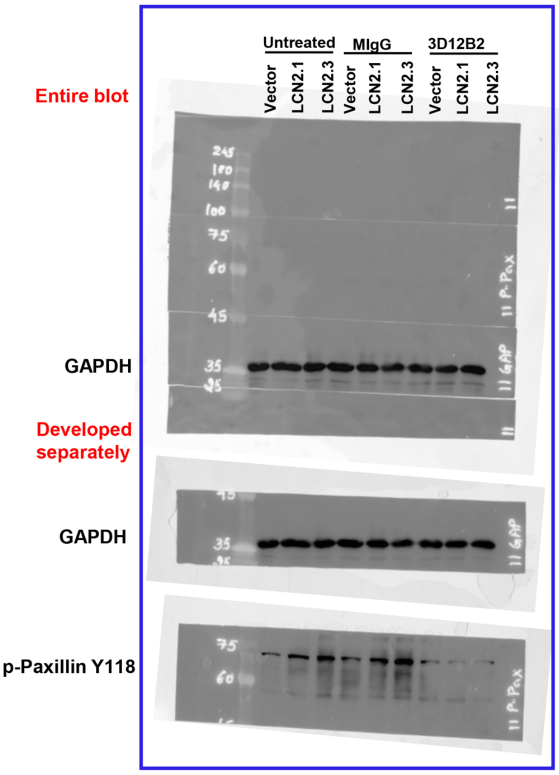

Journal of Cell Science • Supplementary information

Image in the manuscript

Figure 4 G

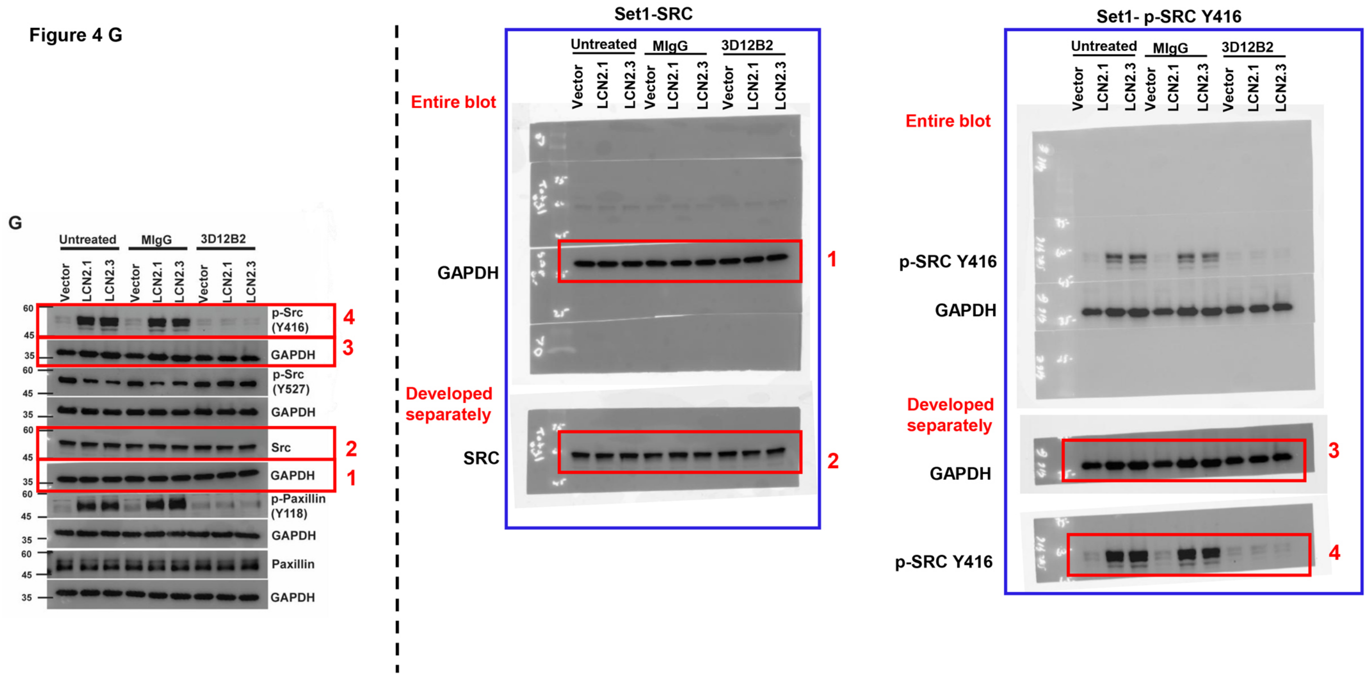

Image in the manuscript

Figure 4 G

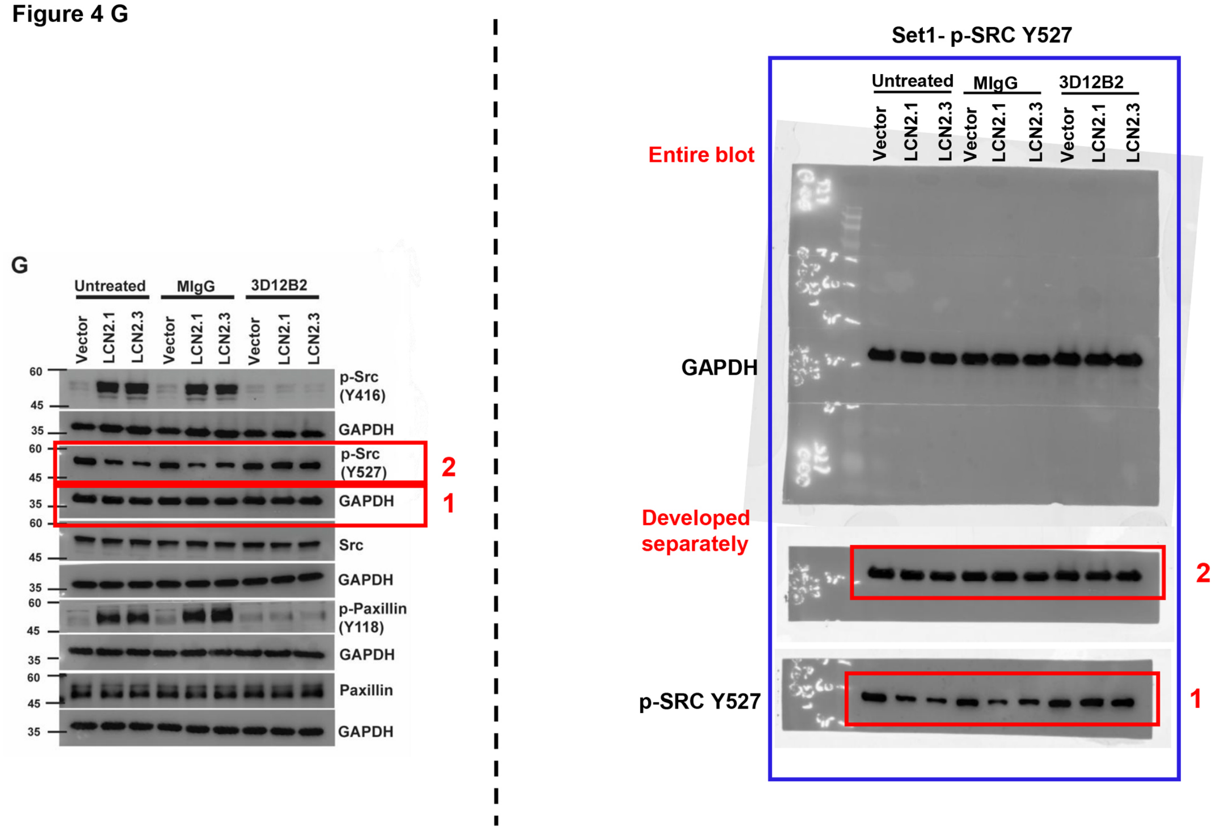

Journal of Cell Science • Supplementary information

Figure 4 G

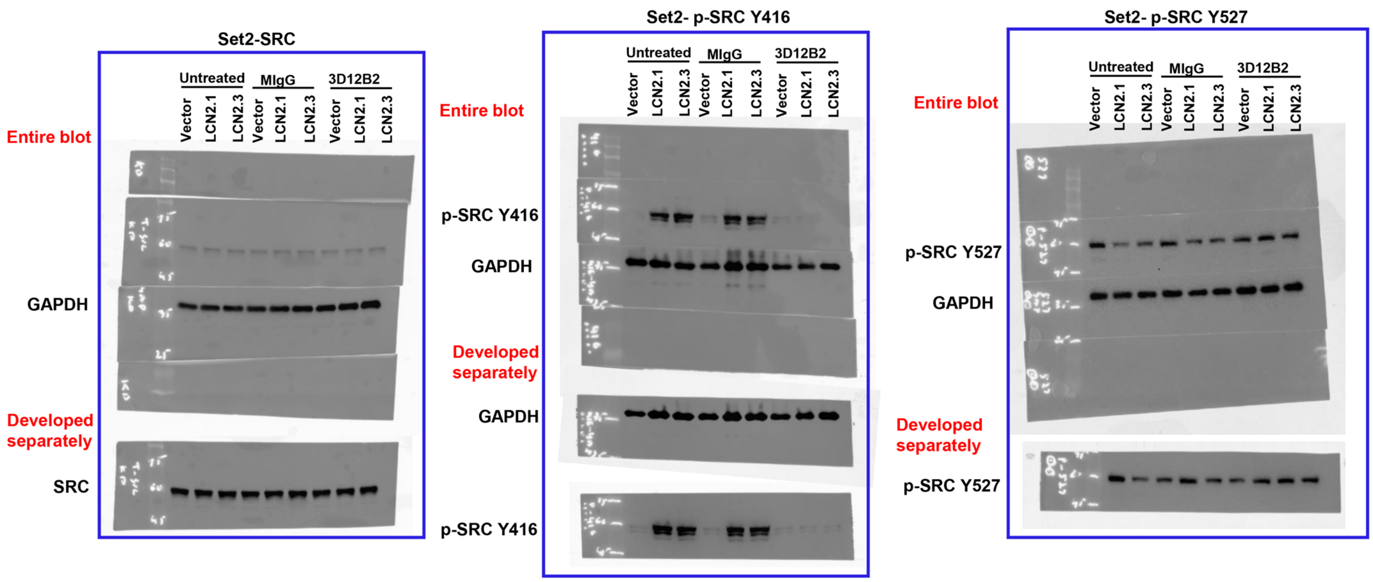

Figure 4 G

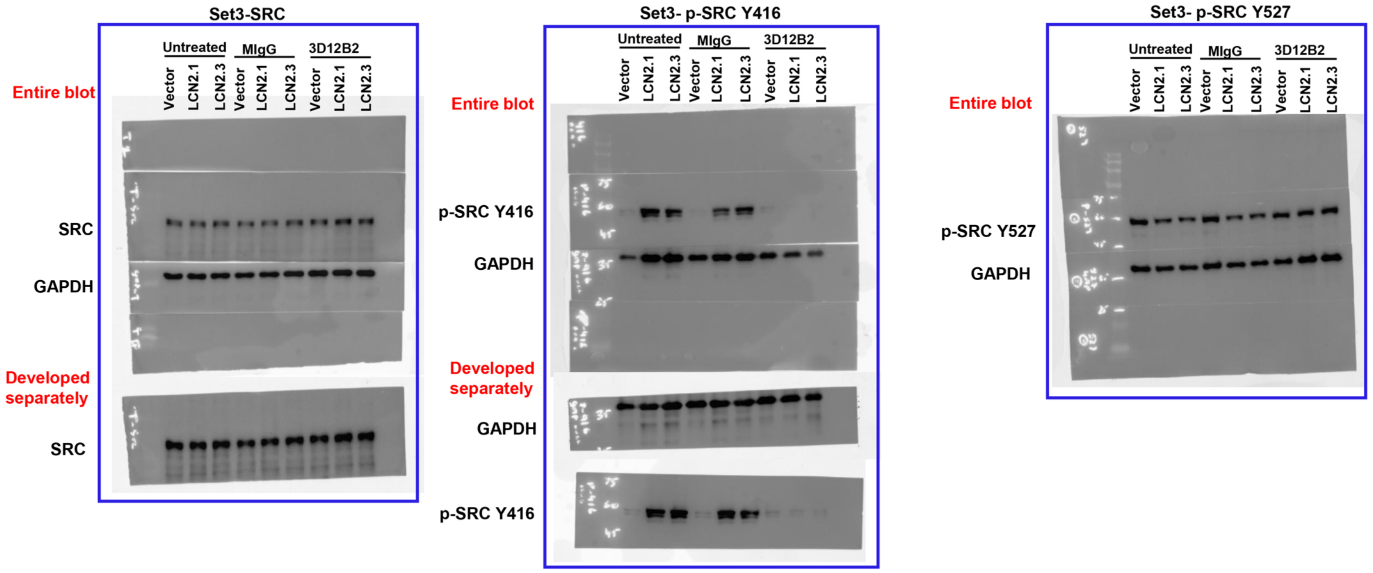

Journal of Cell Science • Supplementary information

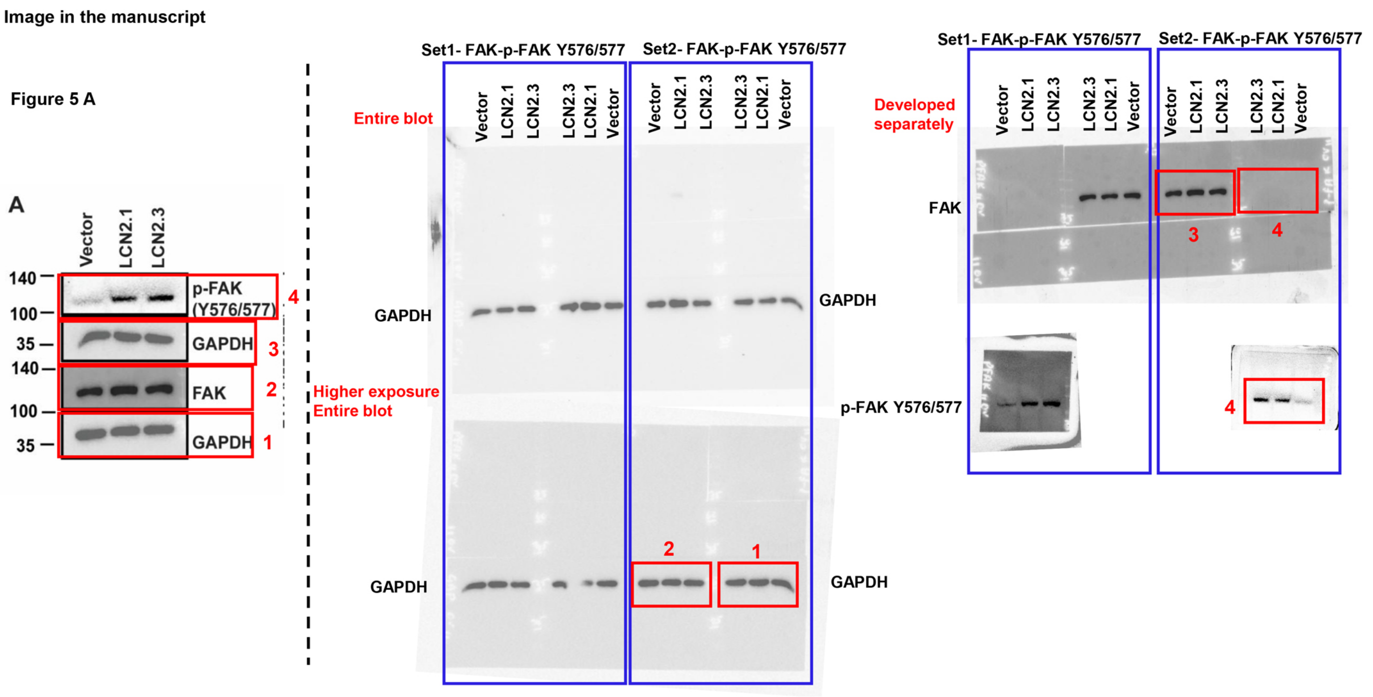

Image in the manuscript
Figure 5 A
A
Vector
LCN2.1
LCN2.3
140
100
35
140
100
35
p-FAK (Y576/577)
GAPDH
FAK
GAPDH
4
3
2
1
Entire blot
Higher exposure Entire blot
Set1- FAK-p-FAK Y576/577
Set2- FAK-p-FAK Y576/577
GAPDH
Developed separately
FAK
p-FAK Y576/577

Figure 5 A

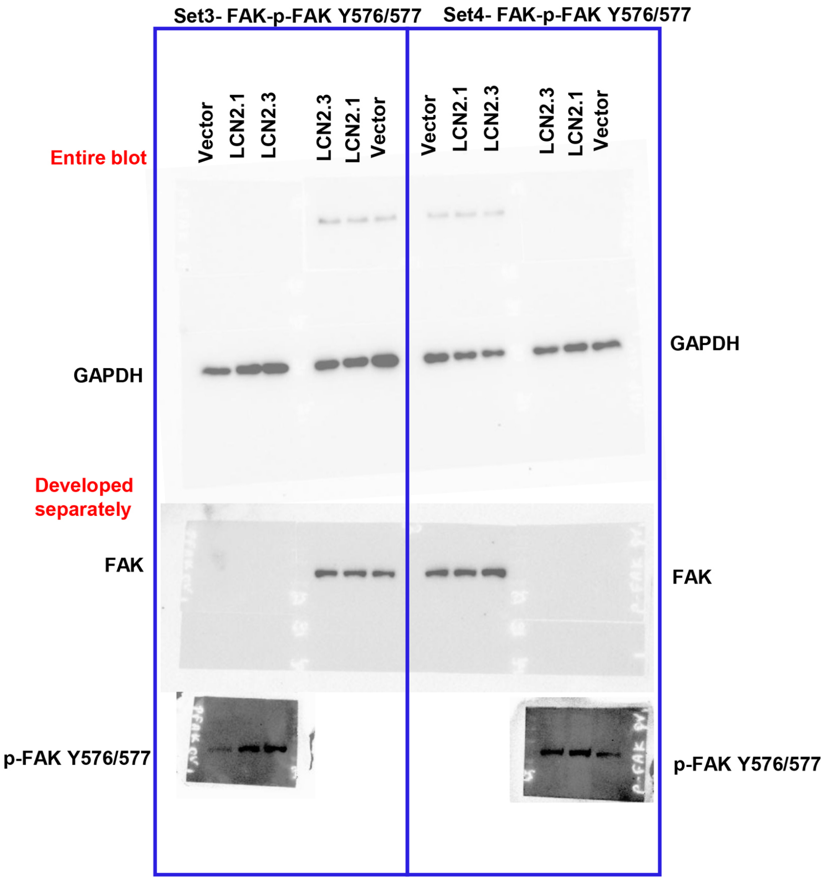

Image in the manuscript

Figure 5 C

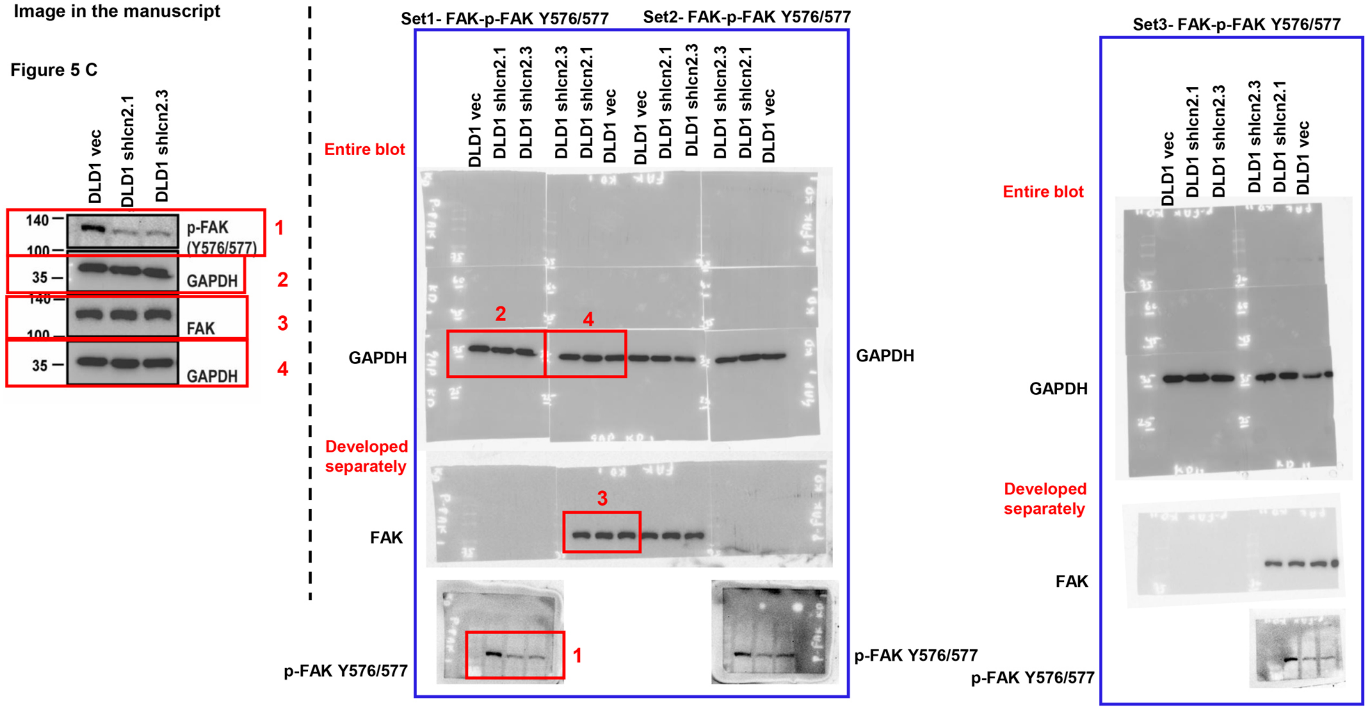

Journal of Cell Science • Supplementary information

Image in the manuscript

Figure 5 E

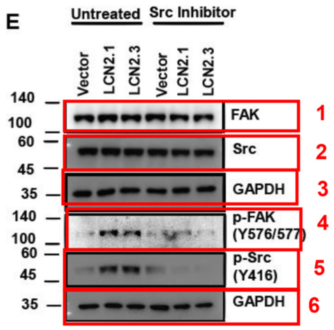

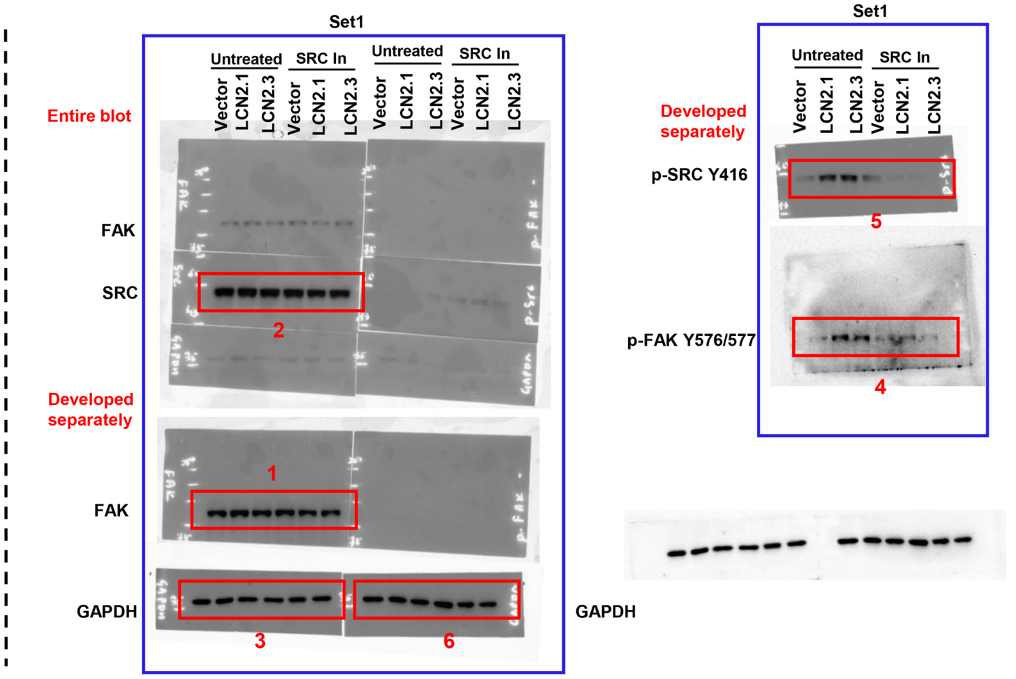

Figure 5 E

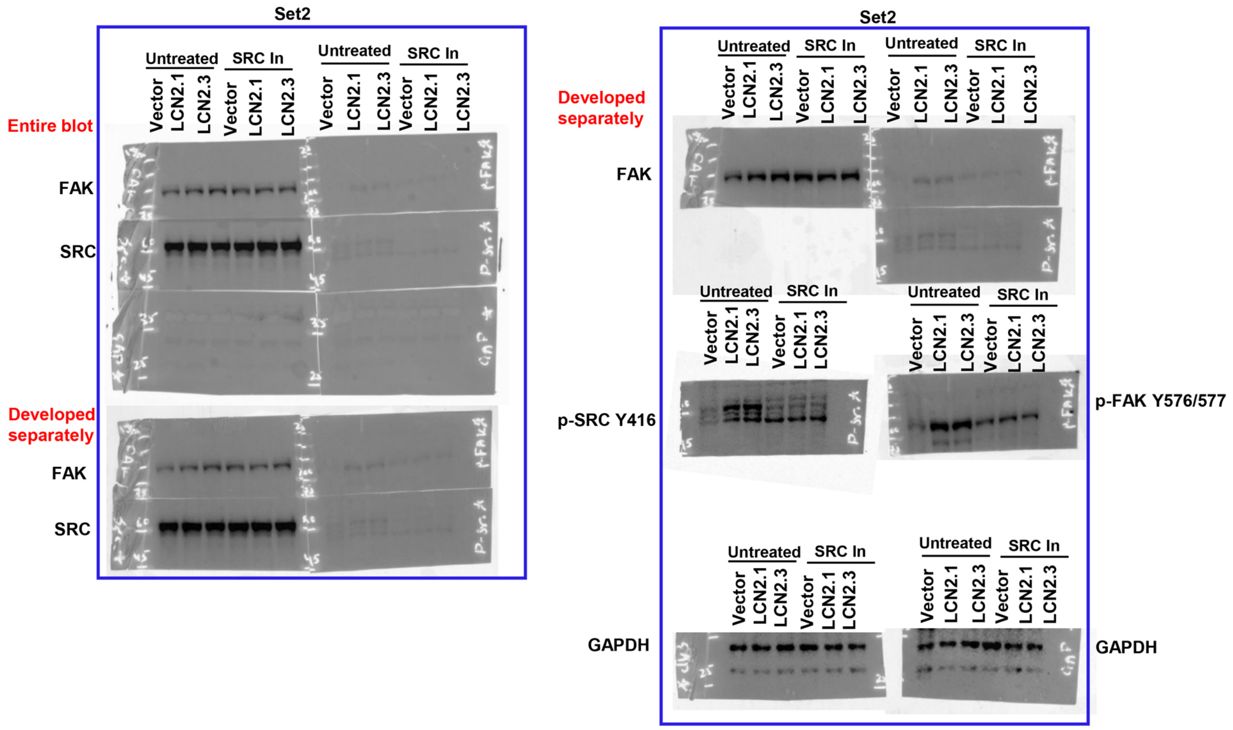

Journal of Cell Science • Supplementary information

Figure 5 E

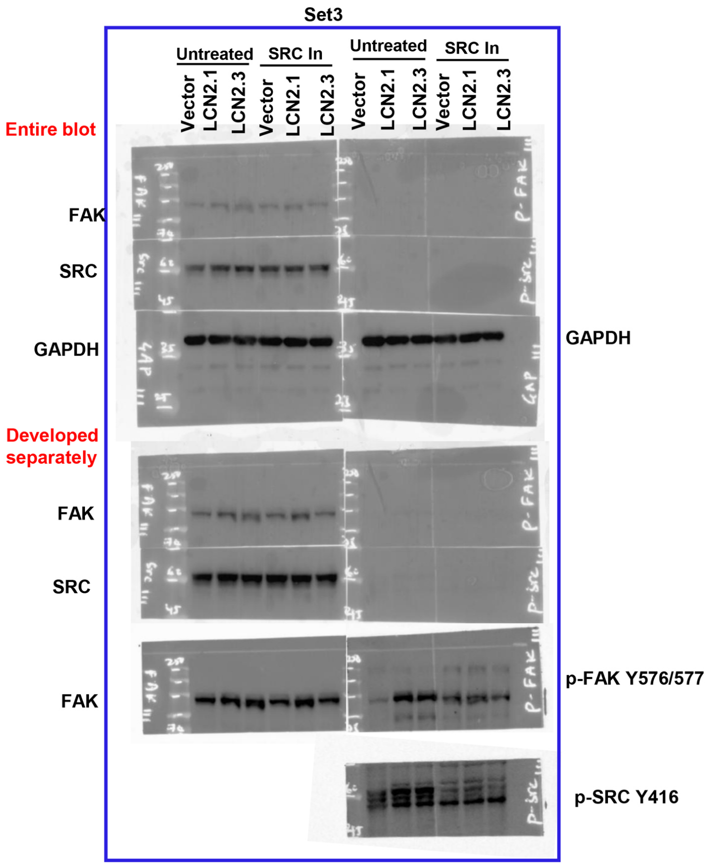

Image in the manuscript

Figure 5 H

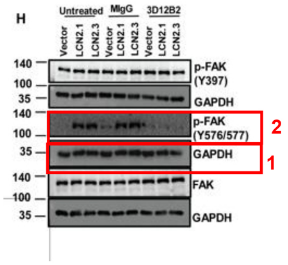

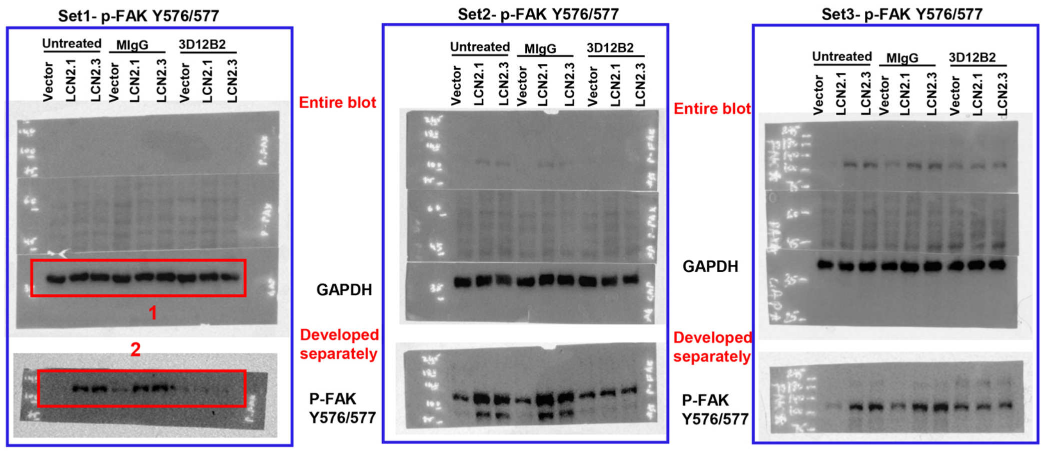

Journal of Cell Science • Supplementary information

Image in the manuscript

Figure 5 H

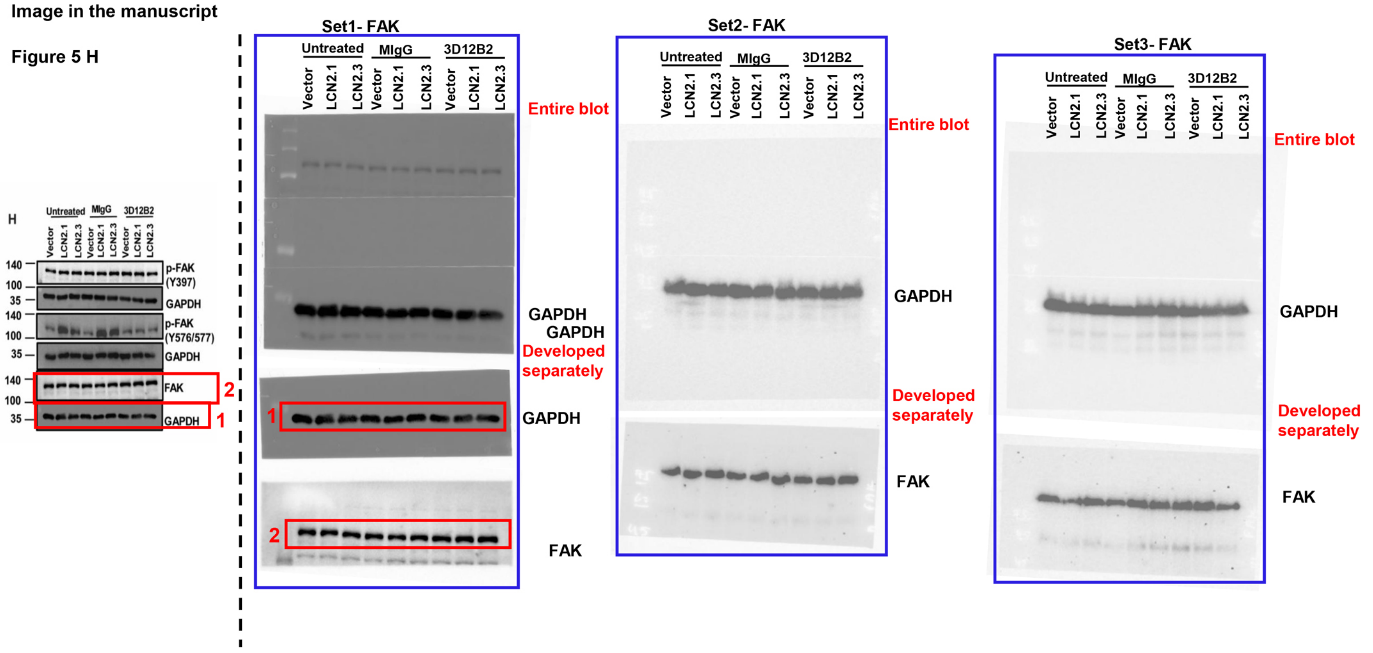

Image in the manuscript

Figure 5 H

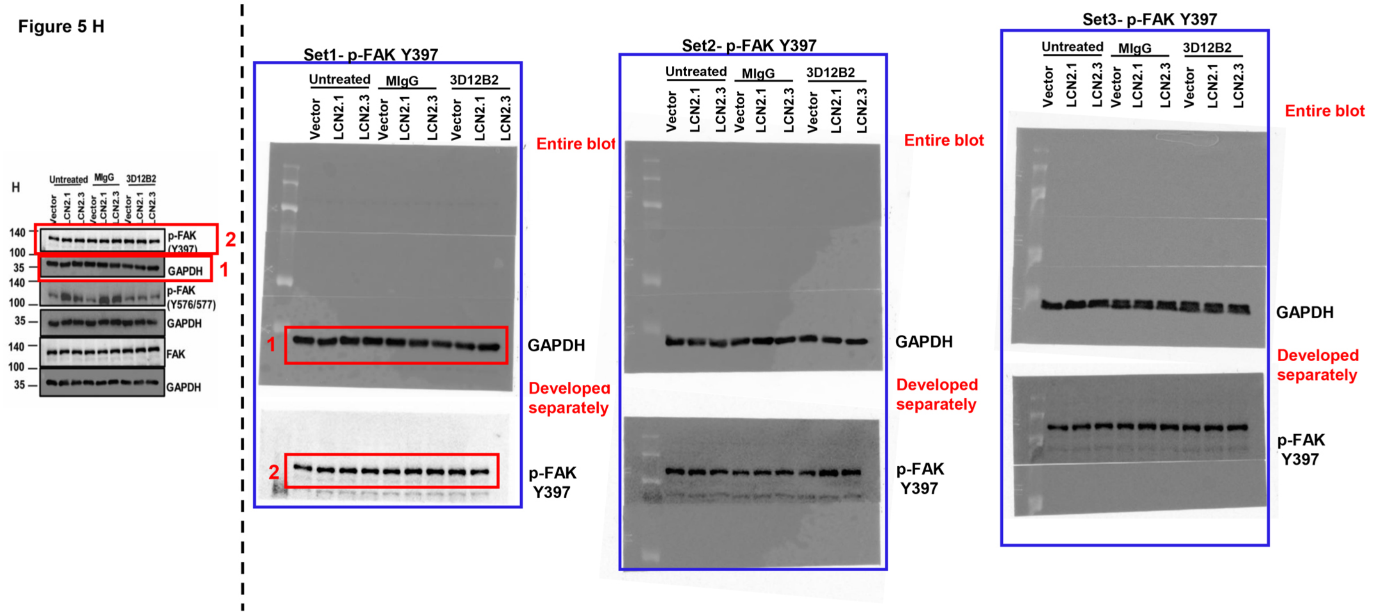

Journal of Cell Science • Supplementary information

Image in the manuscript

Figure 6 A

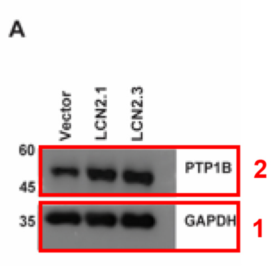

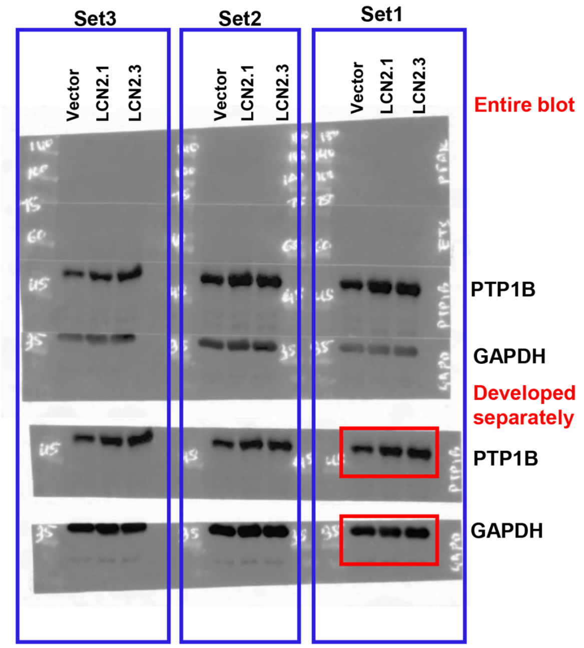

Image in the manuscript

Figure 6 C

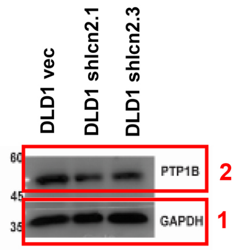

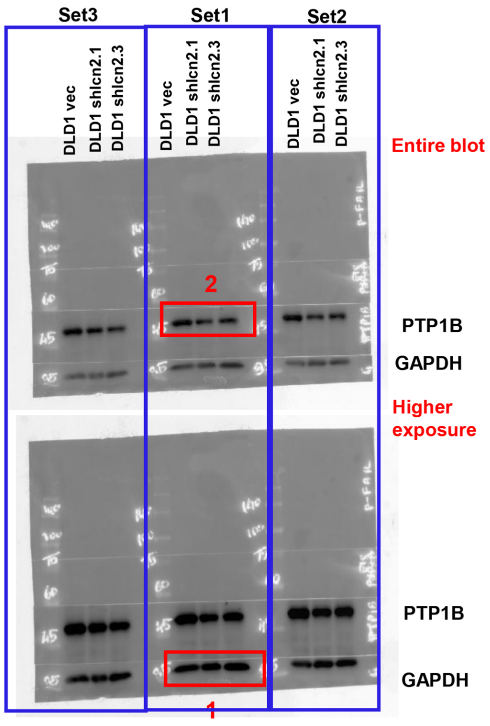

Journal of Cell Science • Supplementary information

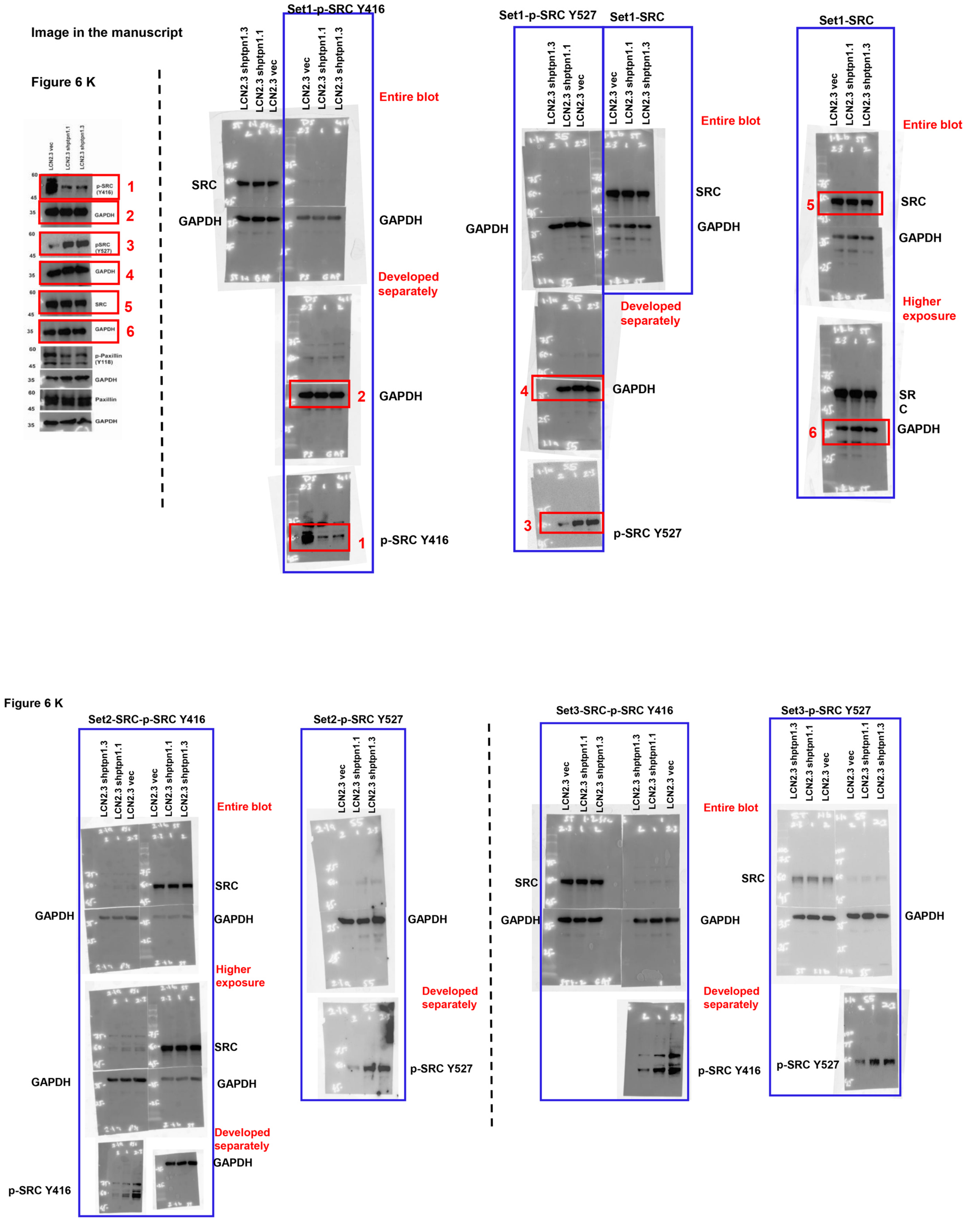

Journal of Cell Science • Supplementary information

Image in the manuscript

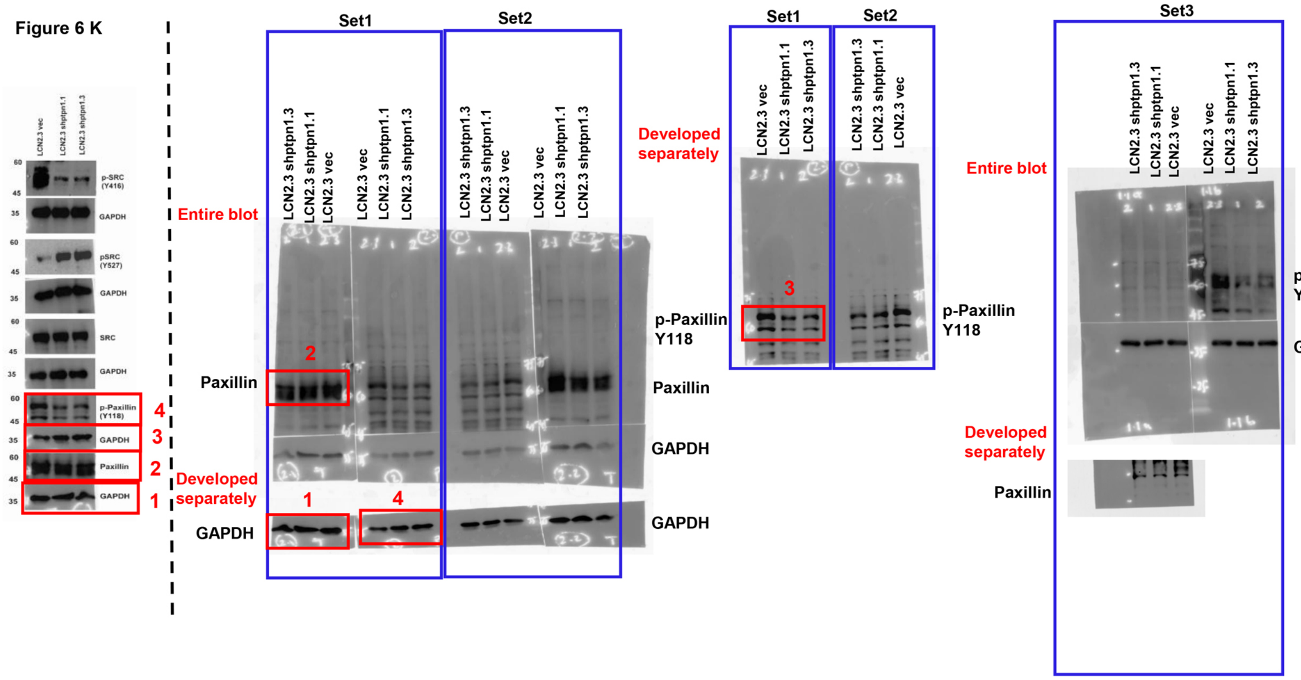

Image in the manuscript

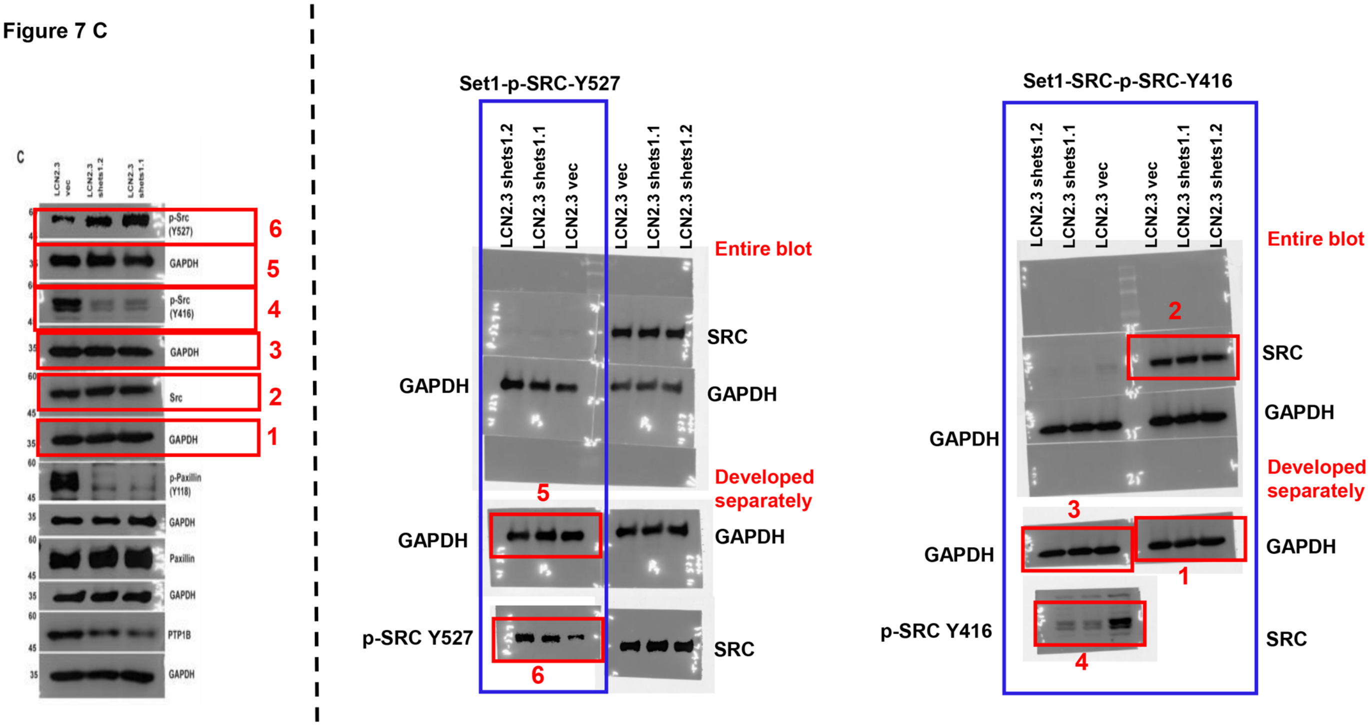

Figure 7 C

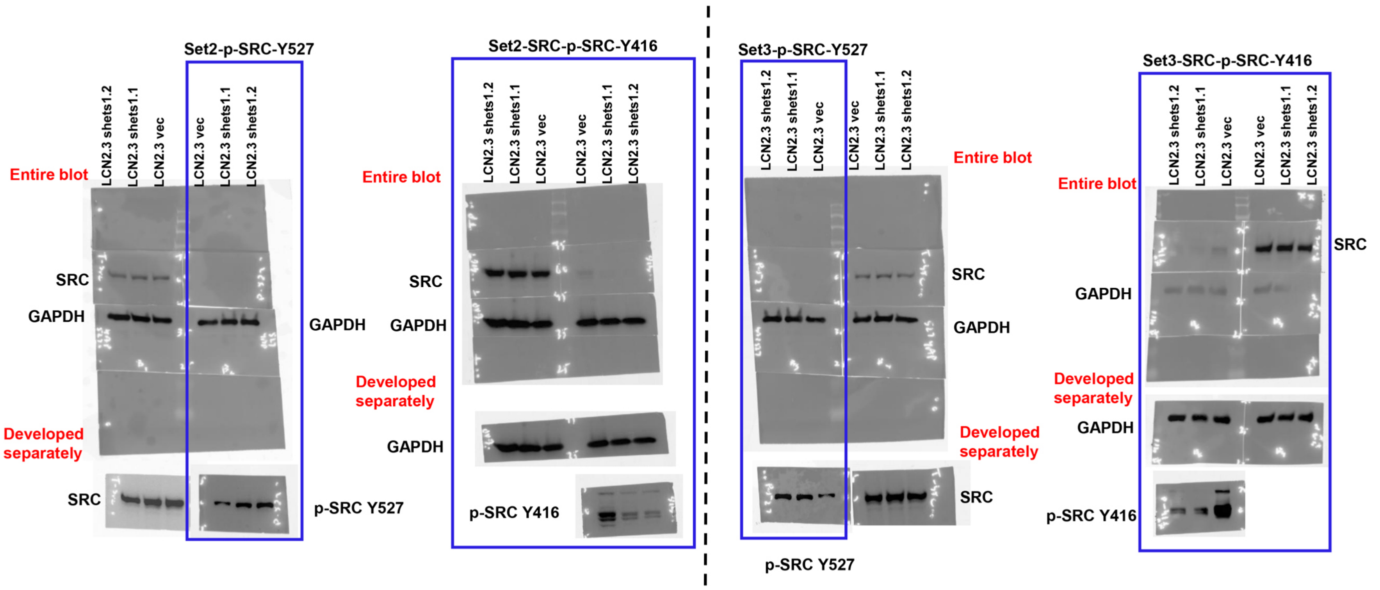

Image in the manuscript

Figure 7 C

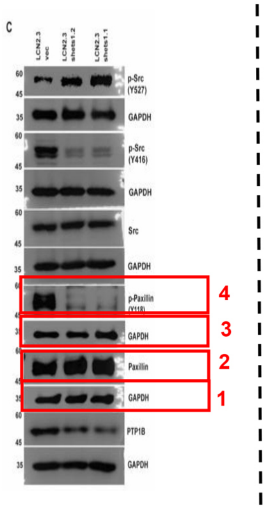

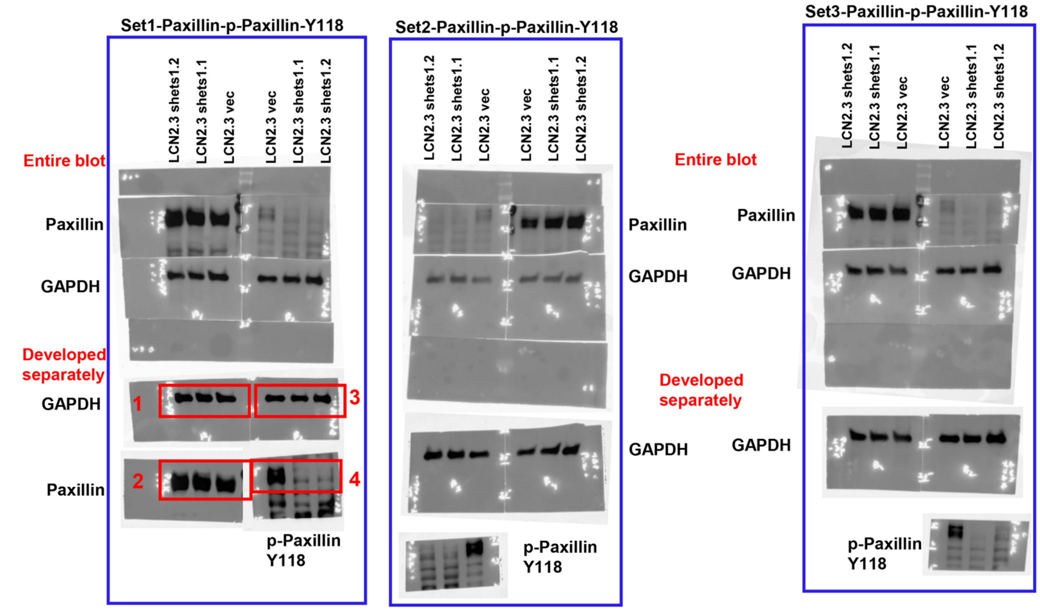

Journal of Cell Science • Supplementary information

Image in the manuscript

Figure 7 C

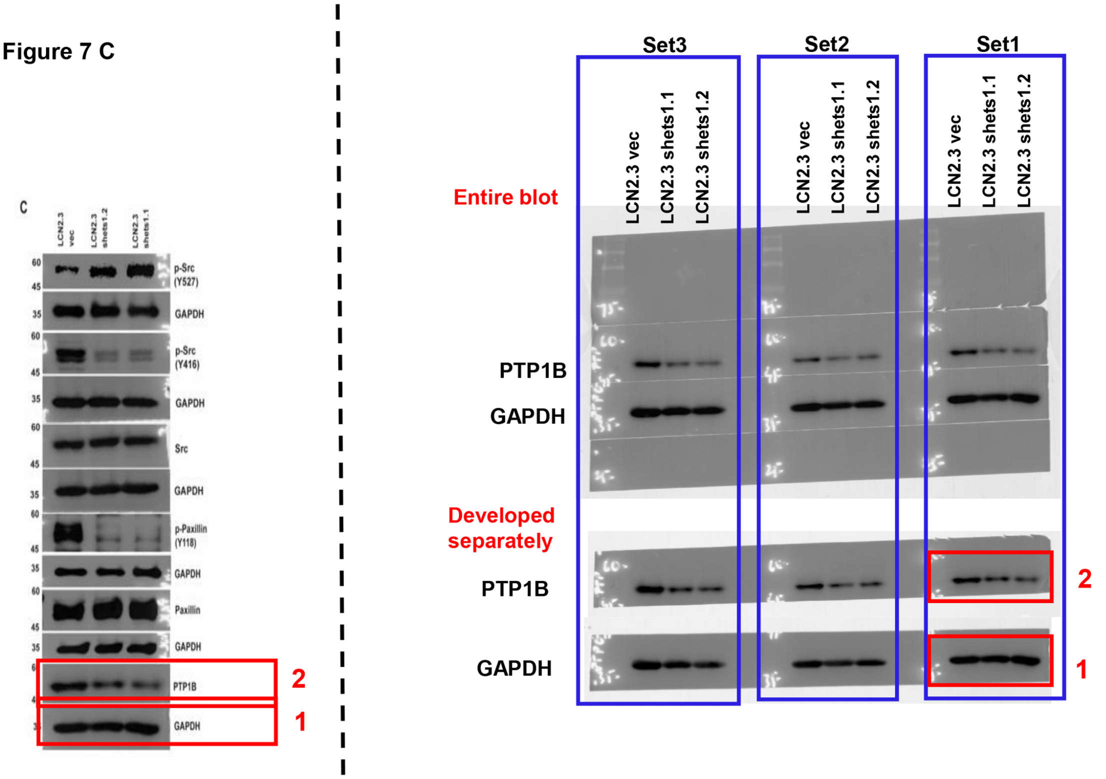

Image in the manuscript

Supplementary figure 1 A

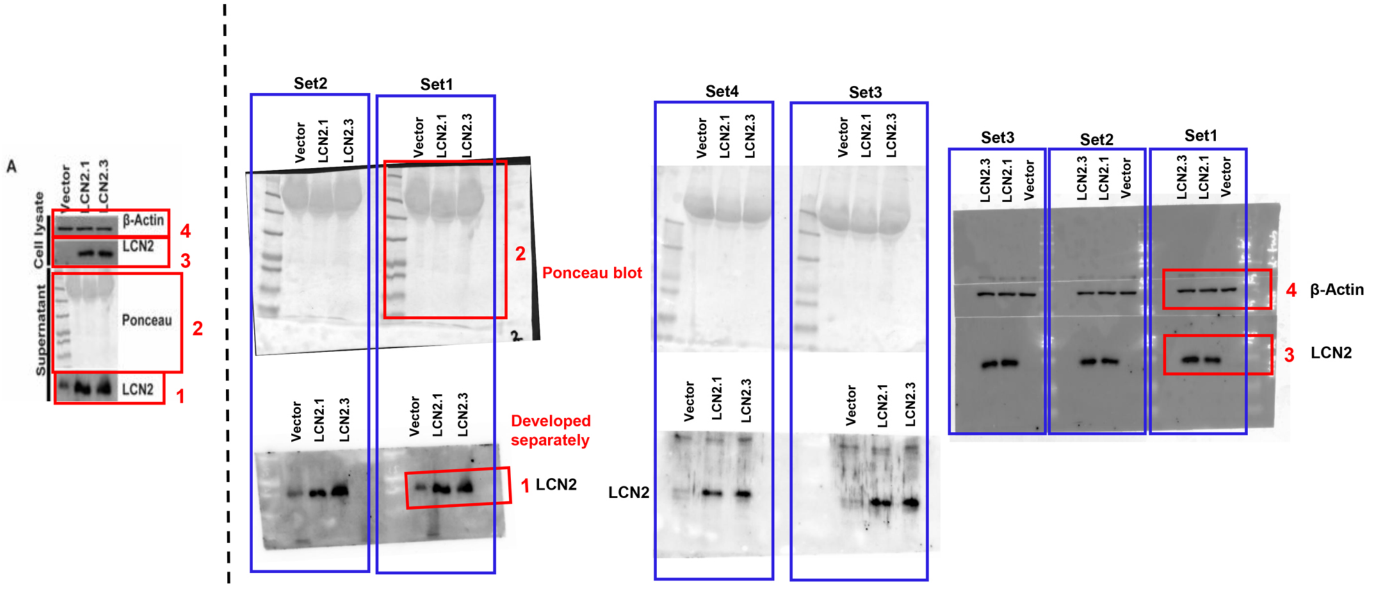

Journal of Cell Science • Supplementary information

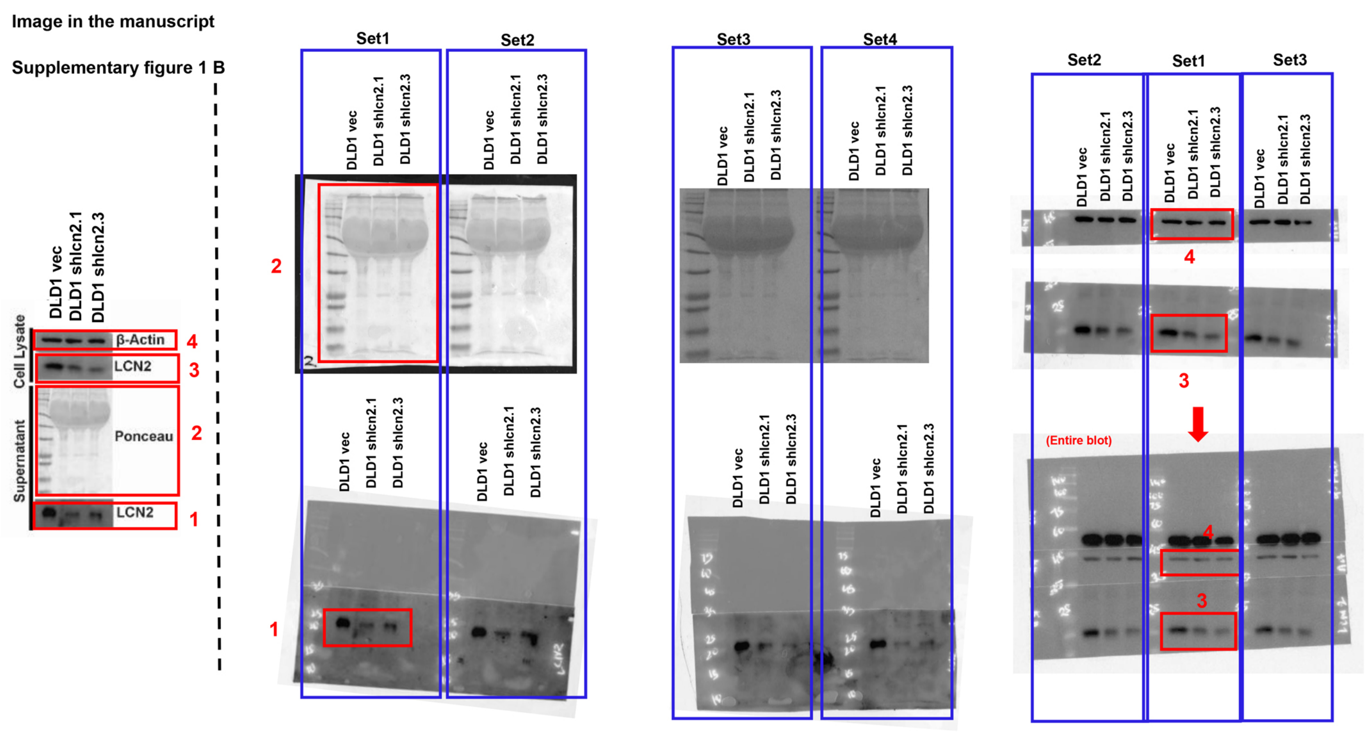

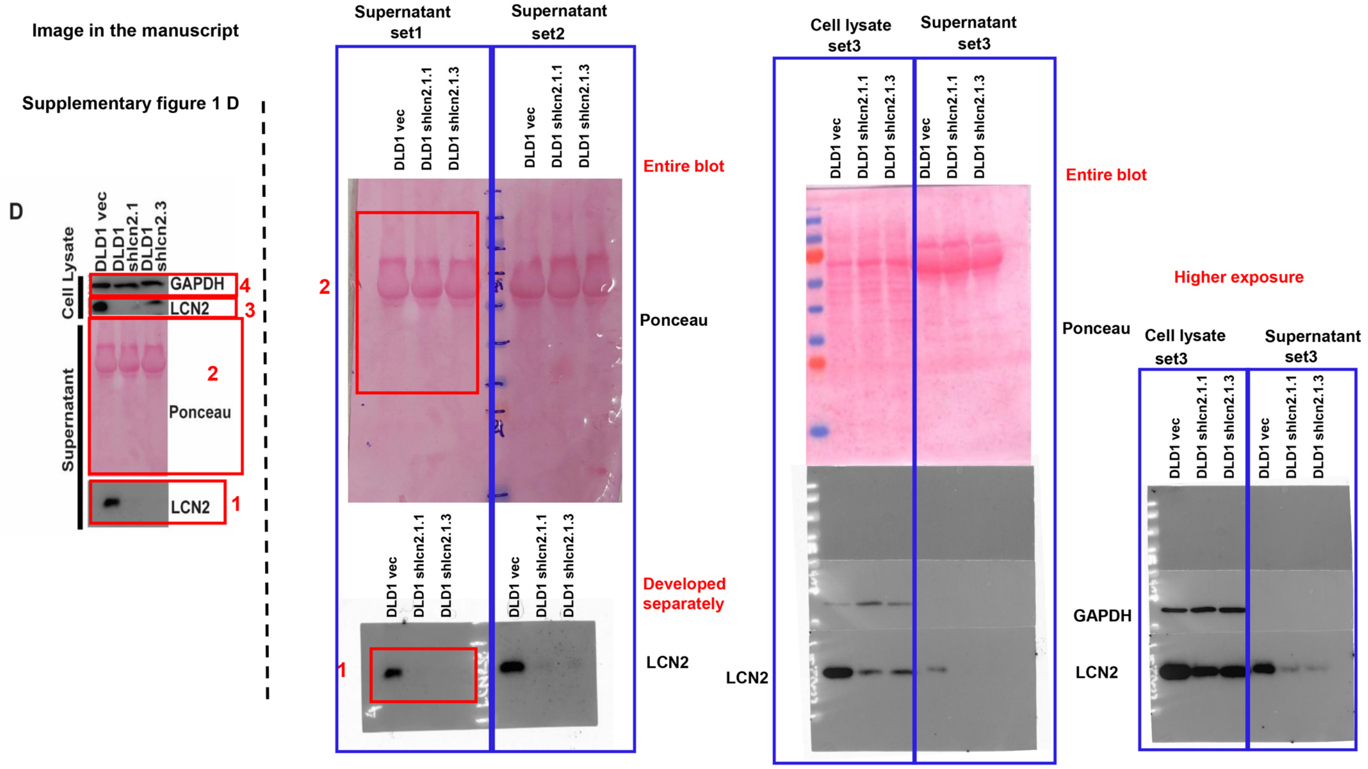

Journal of Cell Science • Supplementary information

J. Cell Sci.: doi:10.1242/jcs.263663: Supplementary information

Image in the manuscript

Supplementary figure 1 D

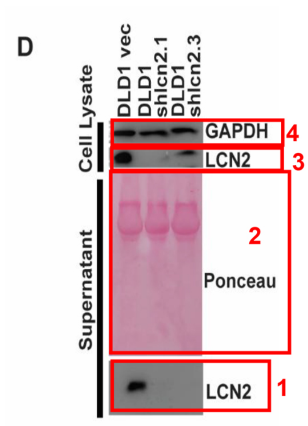

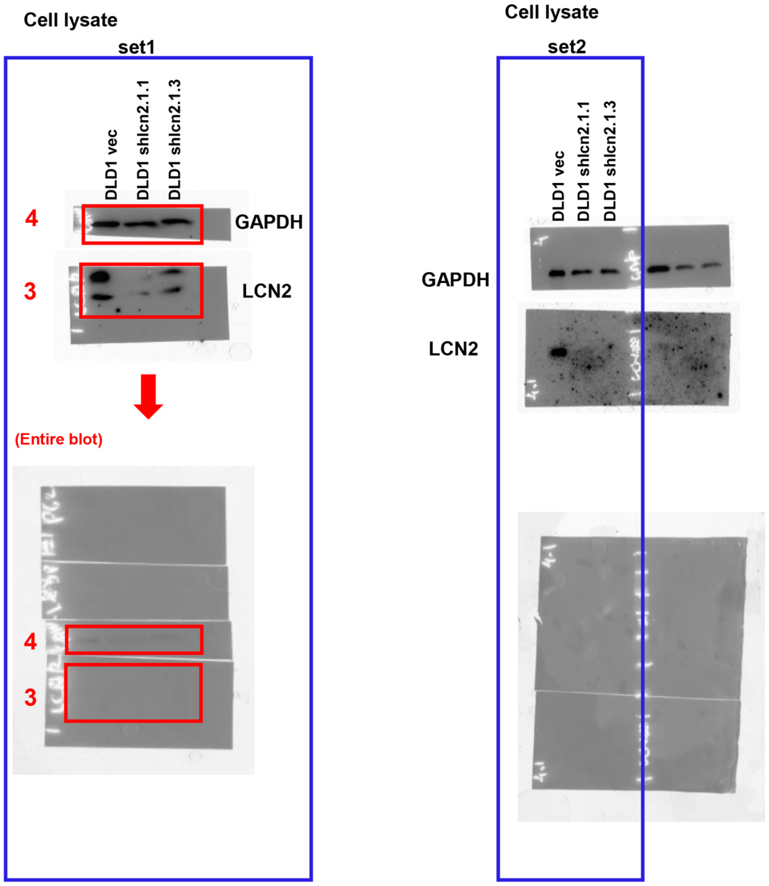

Image in the manuscript

Supplementary figure 3 A

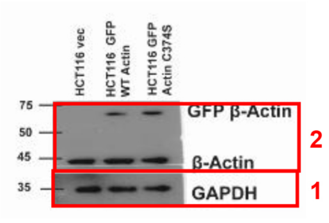

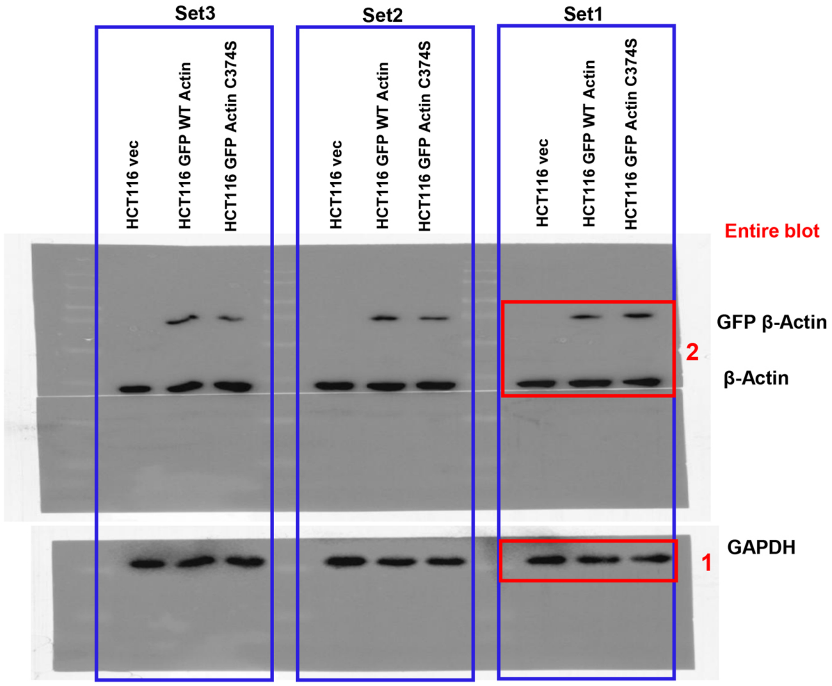

Journal of Cell Science • Supplementary information

Image in the manuscript

Supplementary figure 3 F

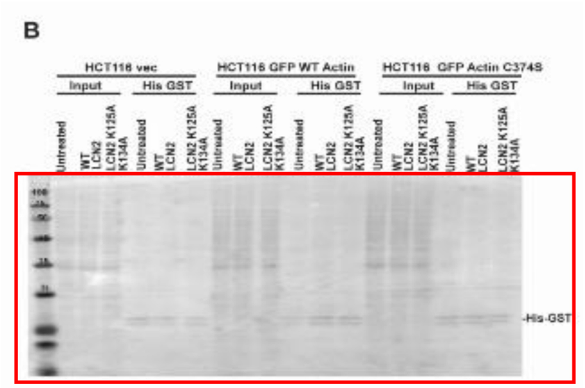

Set1

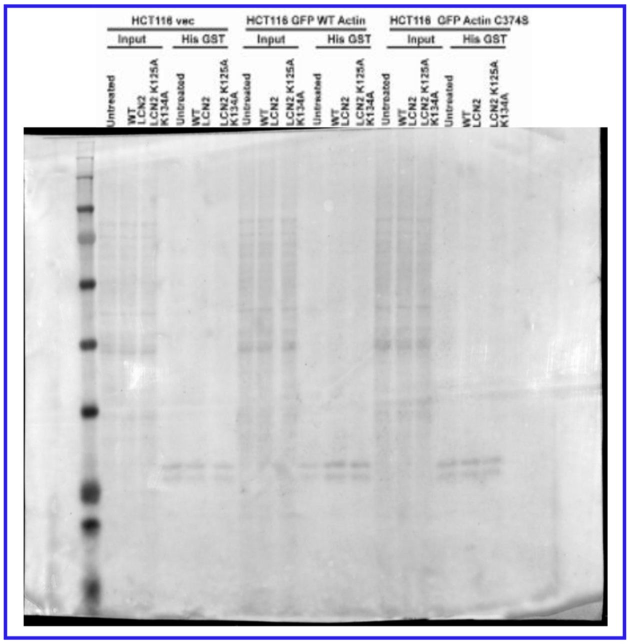

Supplementary figure 3 F

Set2

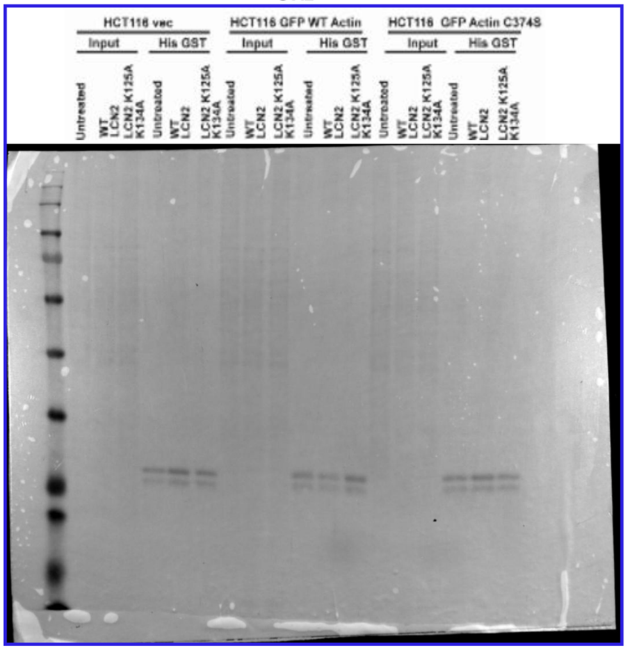

Set3

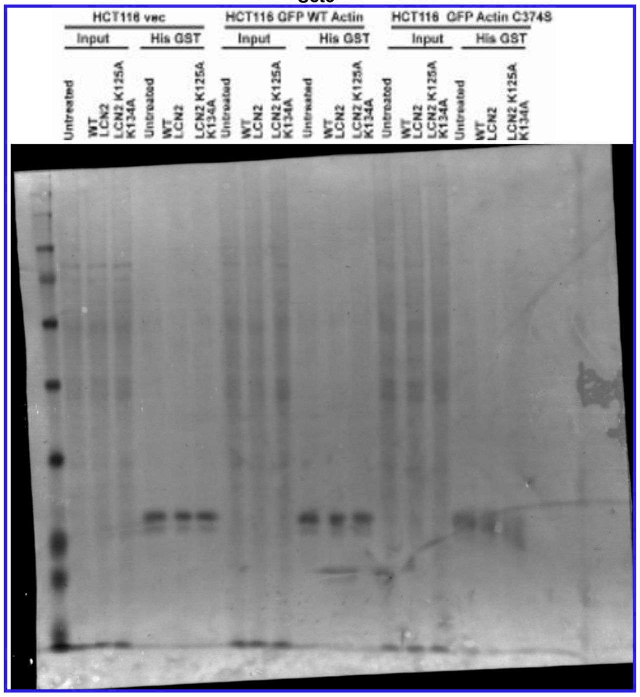

Journal of Cell Science • Supplementary information

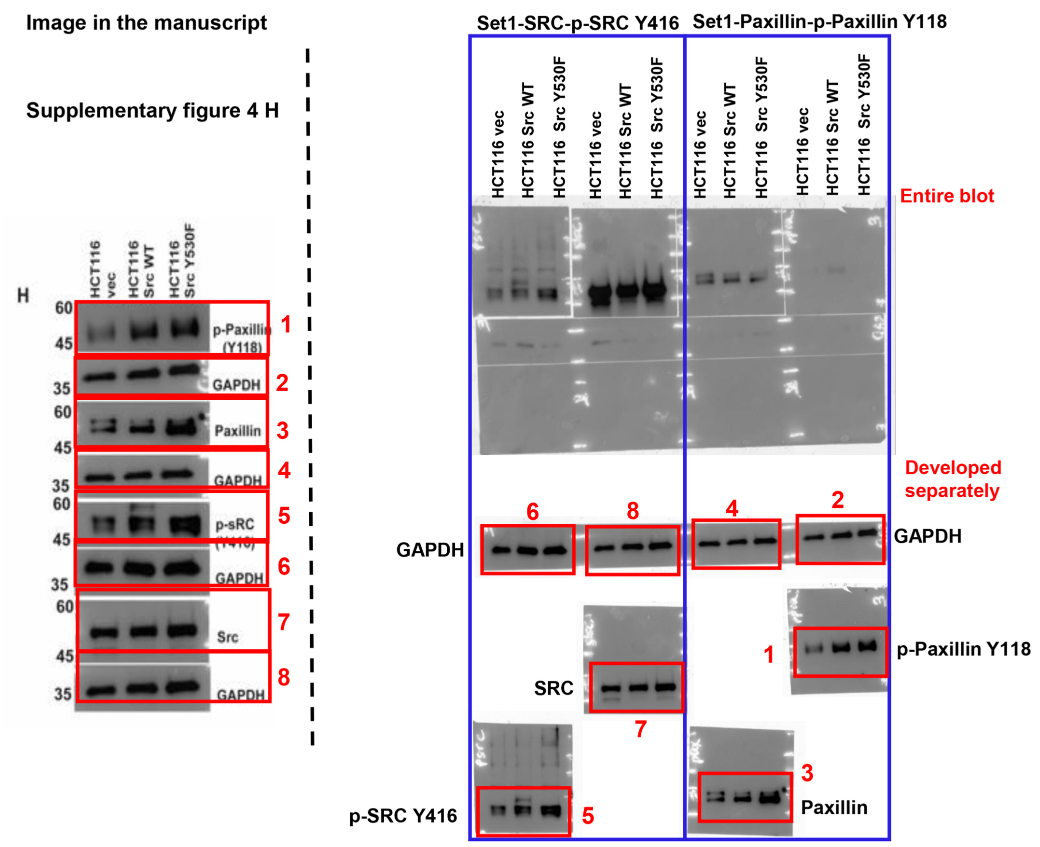

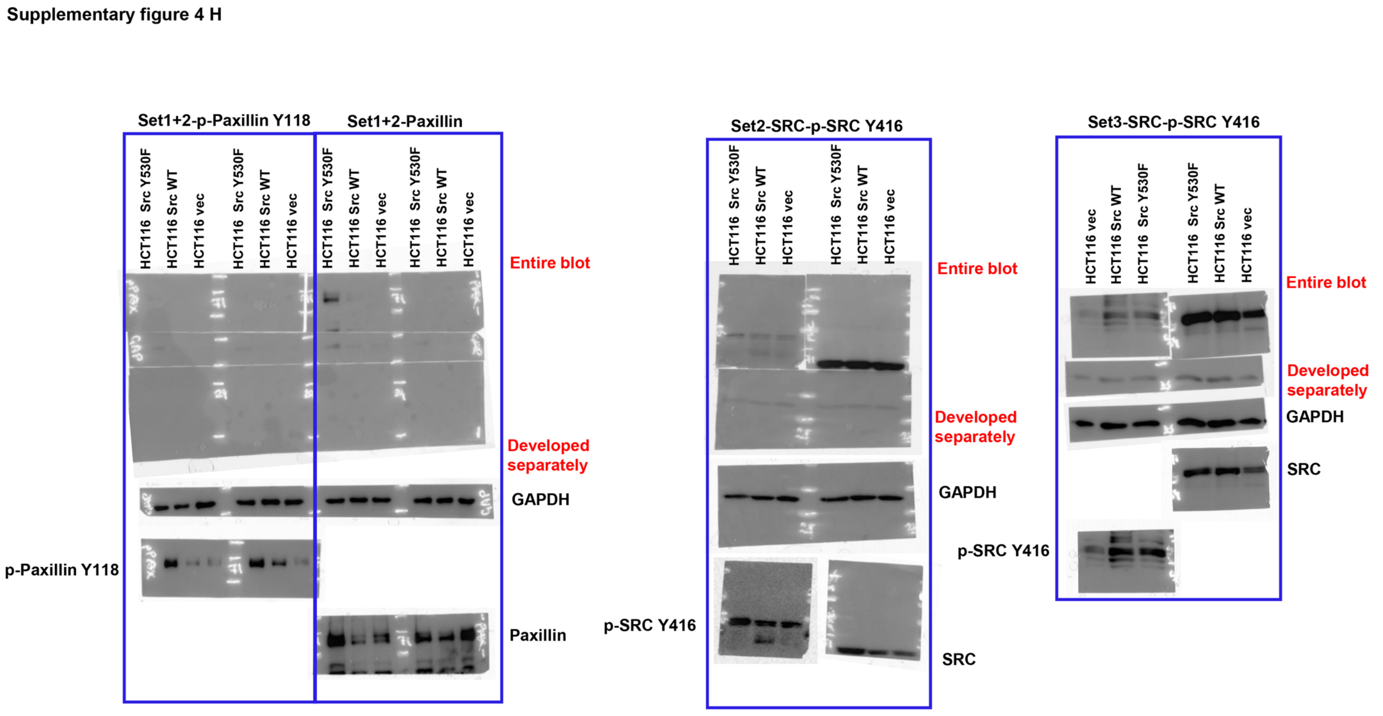

Journal of Cell Science • Supplementary information

Image in the manuscript

Supplementary figure 5 A

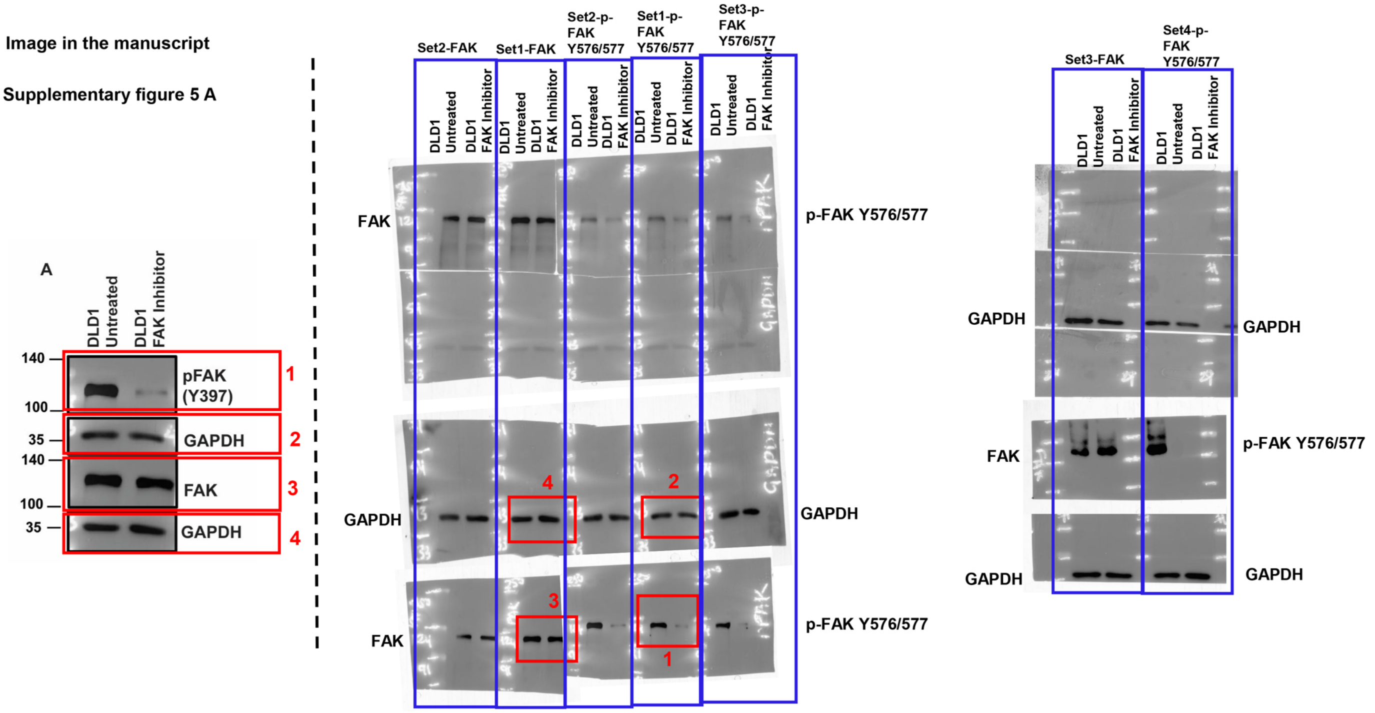

Image in the manuscript

Supplementary figure 6 A

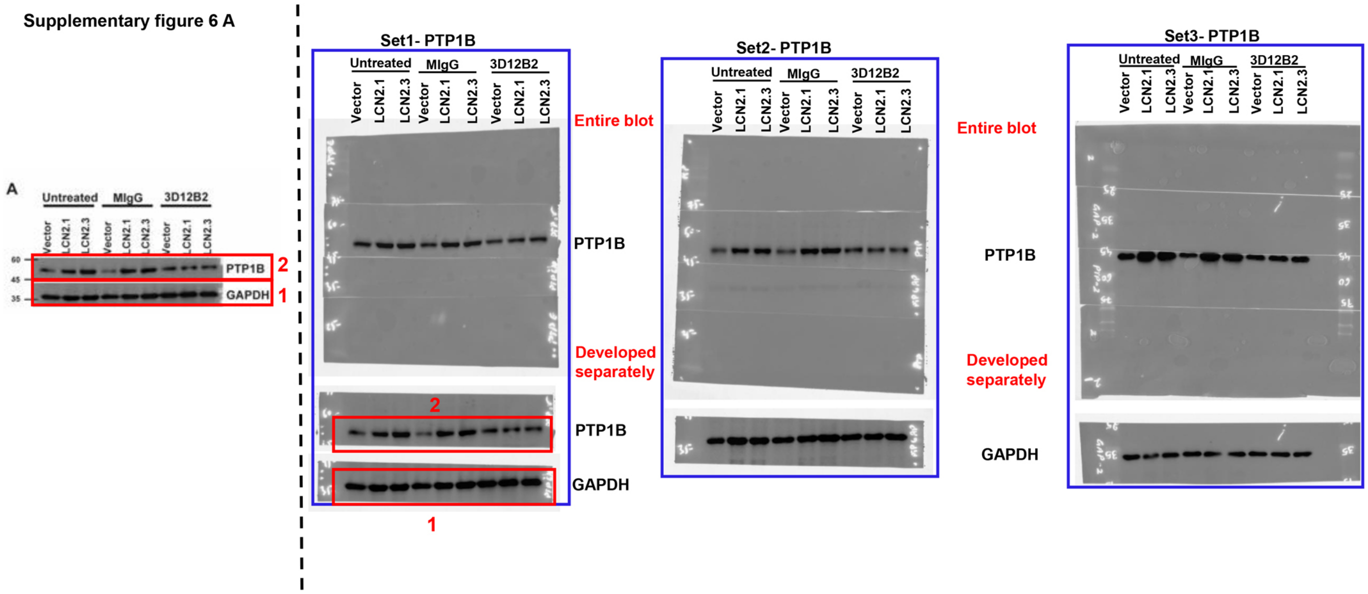

Image in the manuscript

Supplementary figure 6 C

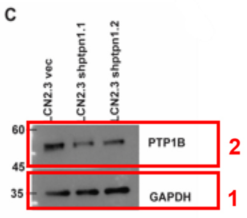

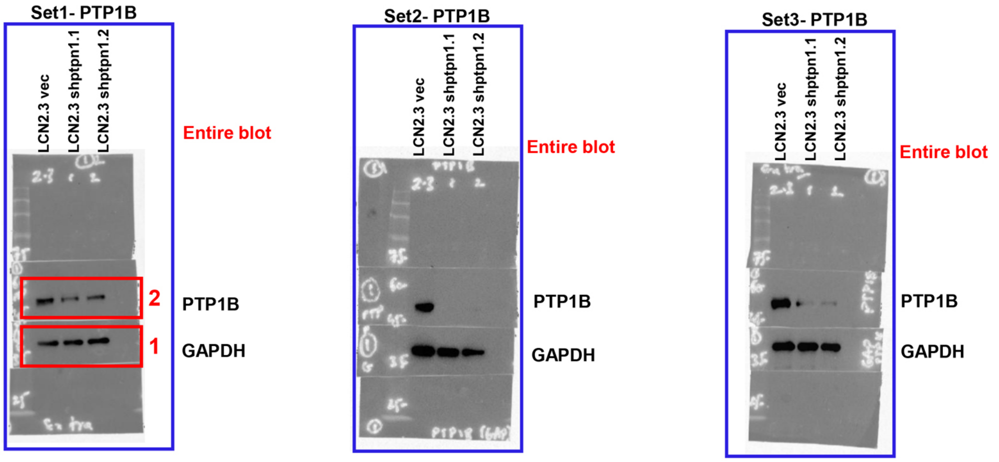

Image in the manuscript

Supplementary figure 7 A

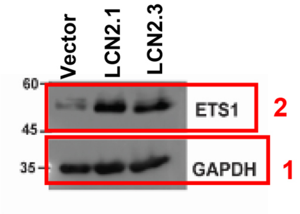

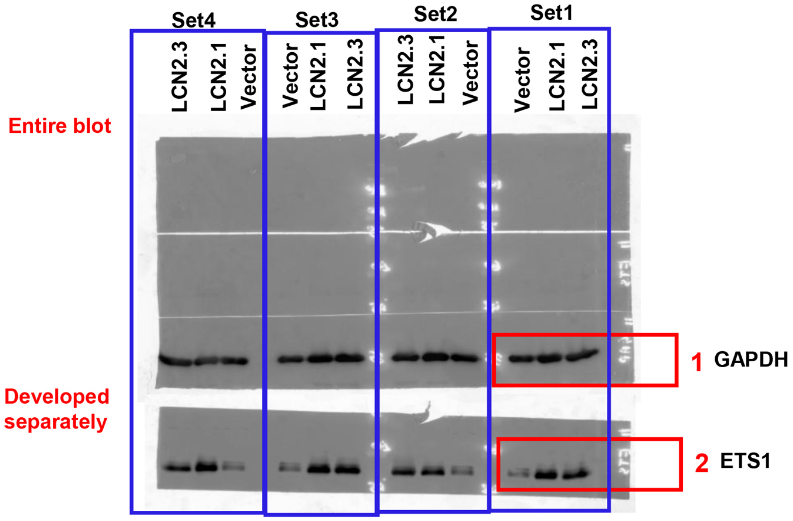

Image in the manuscript

Supplementary figure 7 C

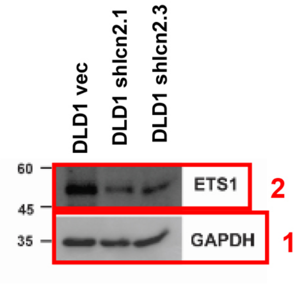

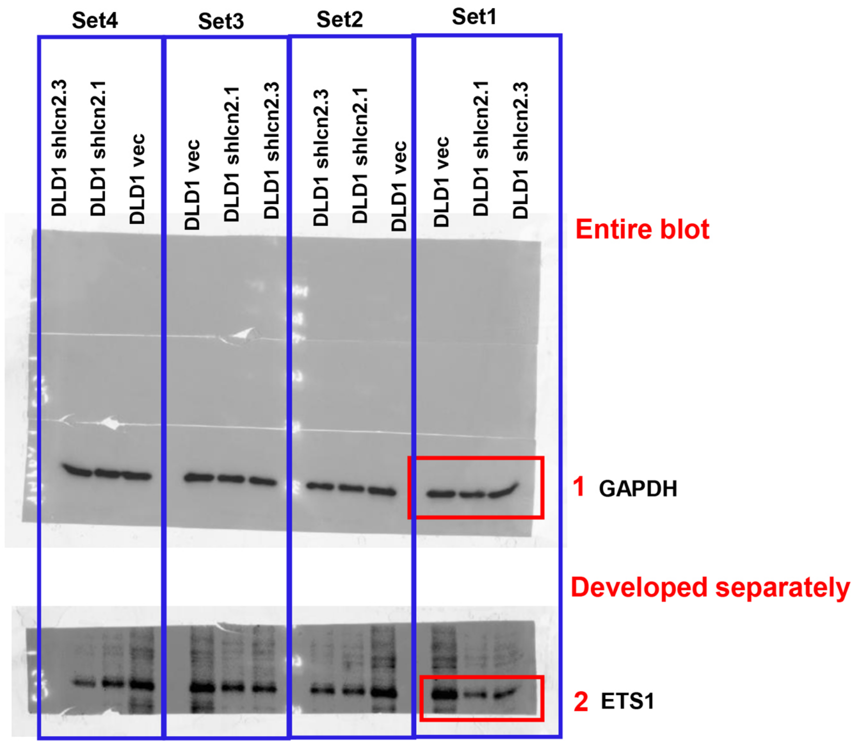

Image in the manuscript

Supplementary figure 7 E

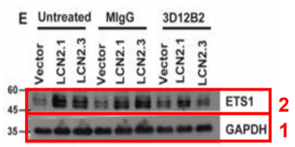

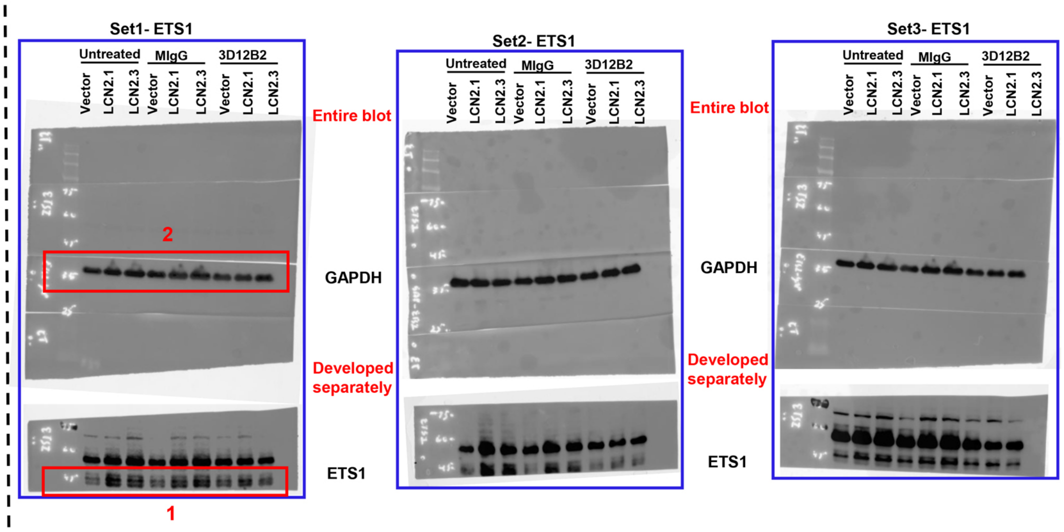

Journal of Cell Science • Supplementary information

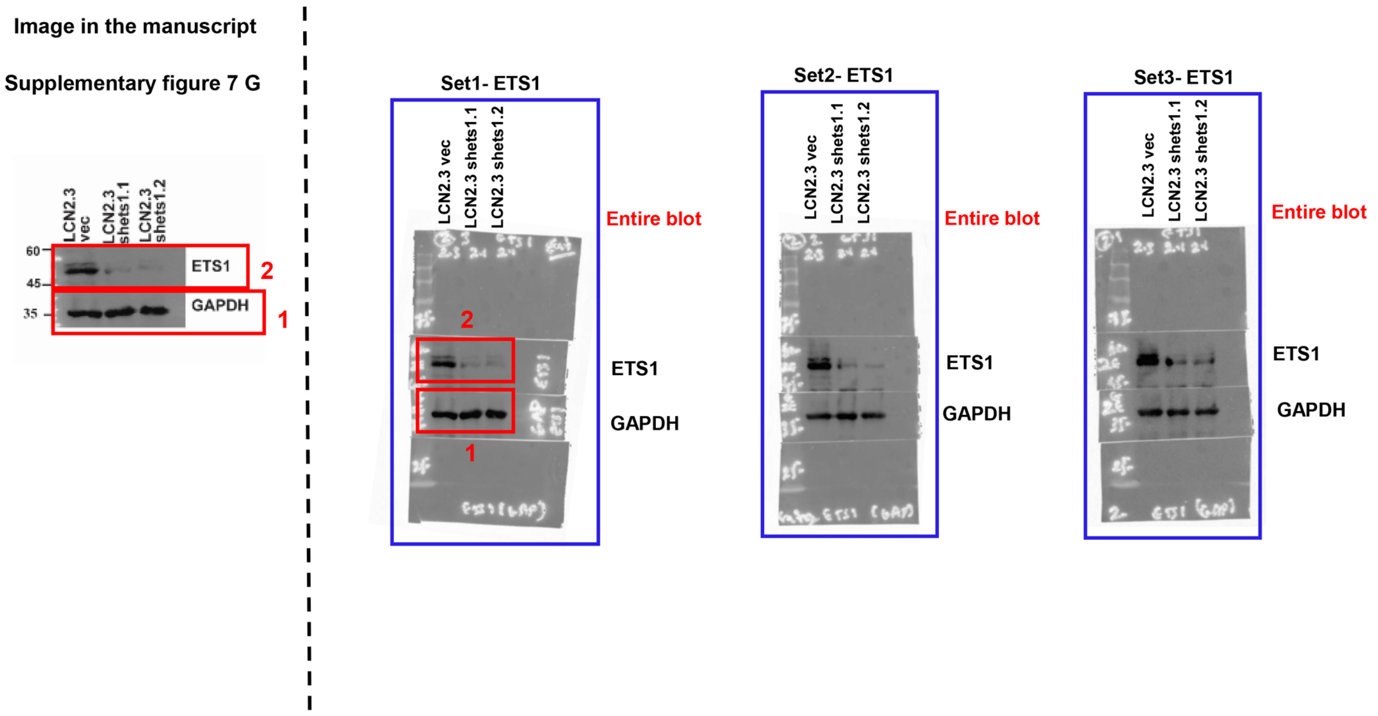

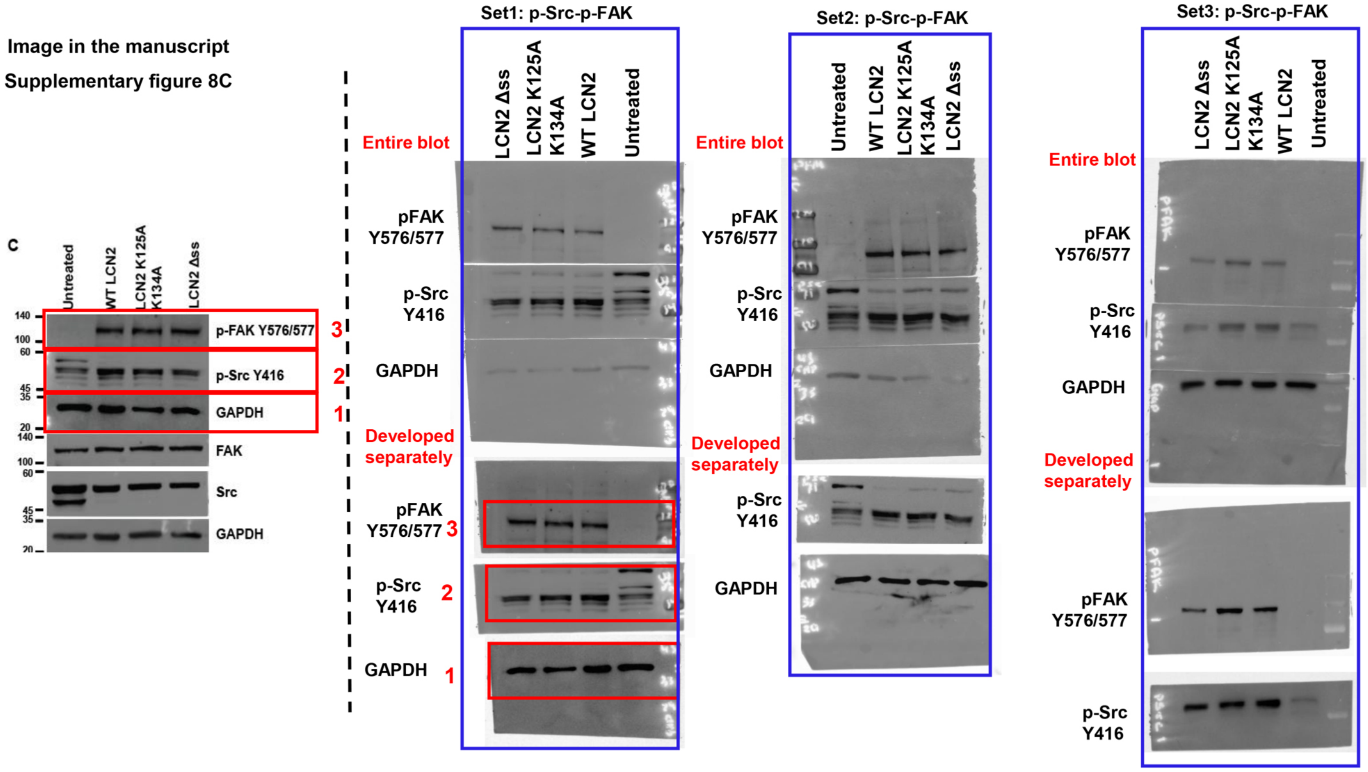

Journal of Cell Science • Supplementary information

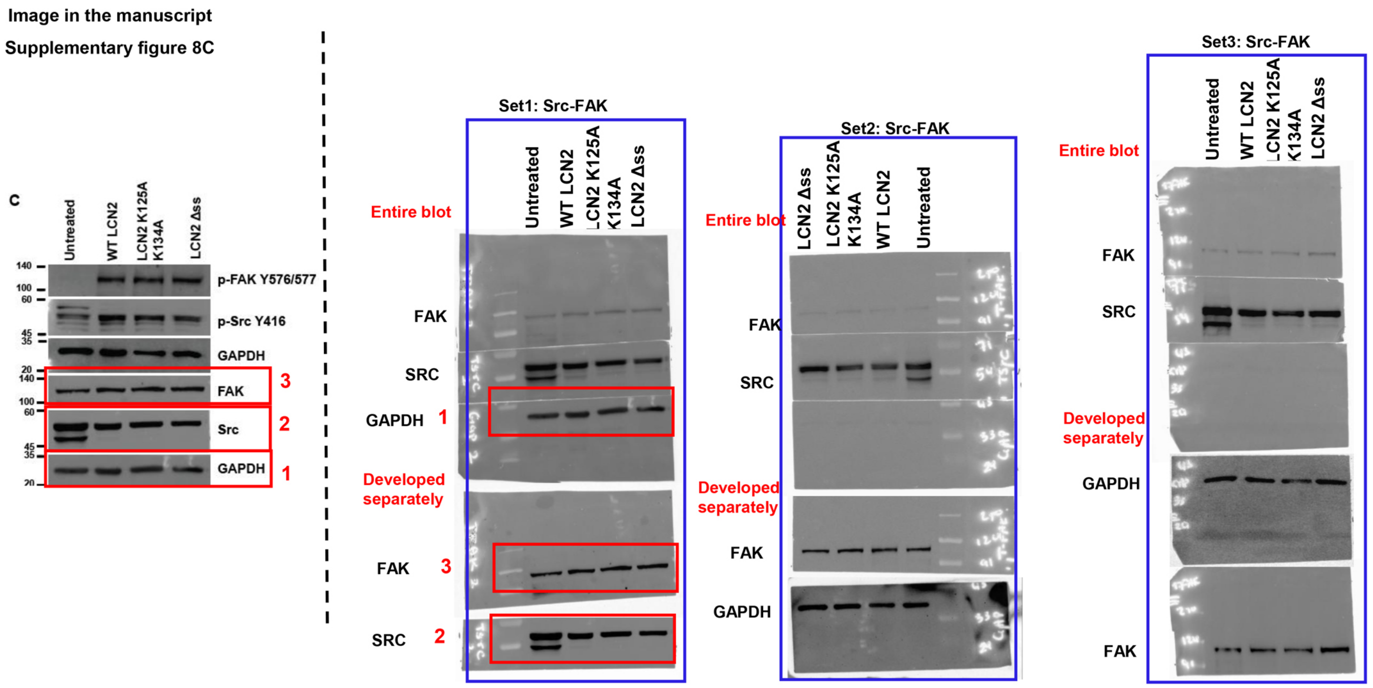

Fig. S9. Western blot data for review.

Table S1. Sequences of oligonucleotides used to generate knockdown and bacterial expression clones, chromatin immunoprecipitation assays, and site-directed mutagenesis.

Name of gene	Sequence (5' to 3')
shRNA PTPN1.1 forward	CCGGGGAAGAGACCCAGGAGGATAACTCGAGTTATCCTCCTGGGTCTCTTCCTTTTTG
shRNA PTPN1.1 reverse	CAAAAAGCTGCTCTGAAATCTCCTTTACTCGAGTAAAGGAGATTTCAGAGCAGCCCGG
shRNA PTPN1.2 forward	CCGGGGAAATGCAGGGAGTTCTTCCCTCGAGGGAAGAACTCCCTGCATTTCCTTTTTG
shRNA PTPN1.2 reverse	CAAAAAGGAAATGCAGGGAGTTCTTCCCTCGAGGGAAGAAC TCCCTGCATTTCCCCGG
C374S forward	TCCACCGCAAATCCTTCTAGGGATCC
C374S reverse	GGATCCCTAGAAGGATTTGCGGTGGA
PTPN1 promoter forward	GCGTAGTGAAACATGATACTCCTG
PTPN1 promoter reverse	GAGTATGGGAGGAGGGCAC
LCN2Δss forward	TCAGGATCCATGCAGGACTCCACCTCAGAC
LCN2Δss reverse	TCACTCGAGGCCGTCGATACACTG

Table S2. Antibodies and dilutions used for Western blot analysis.

Protein	Species	Type	Catalog number	Company	Dilution
β actin	Mouse	Monoclonal	A5316	Sigma	1:5000
LCN2	Goat	Monoclonal	AF1757	R&D Systems	1:2000
ETS1	Rabbit	Monoclonal	14069	Cell Signaling Technology	1:1000
GAPDH	Rabbit	Monoclonal	2118	Cell Signaling Technology	1:8000
α tubulin	Mouse	Monoclonal	ab7291	Abcam	1:5000
PTP1B	Rabbit	Monoclonal	MA5-29512	Invitrogen	1:1000
Paxillin	Rabbit	Monoclonal	12065	Cell Signaling Technology	1:1000
p-Paxillin (Y118)	Rabbit	Monoclonal	69363	Cell Signaling Technology	1:1000
FAK	Rabbit	Polyclonal	285	Cell Signaling Technology	1:1000
p-FAK	Rabbit	Polyclonal	283	Cell Signaling Technology	1:1000
Src	Rabbit	Polyclonal	2108	Cell Signaling Technology	1:1000
p-Src Y 416	Rabbit	Polyclonal	6943	Cell Signaling Technology	1:1000
p-Src Y 527	Rabbit	Polyclonal	2105	Cell Signaling Technology	1:1000
Anti-mouse	Goat	Secondary	A28177	Life Technologies	1:2500
Anti-Rabbit	Goat	Secondary	21234	Life Technologies	1:5000
Anti-Goat	Donkey	Secondary	sc2020	Santa Cruz	1:1000

Table S3. Working dilutions of antibodies and tagged phalloidin that were used for fluorescence assays.

Protein	Source	Company	Catalog number	Dilution
FITC phalloidin		Sigma	P-5282	1:200
TRITC phalloidin		Sigma	P-1951	1:200
Alexa fluor R-488	Rabbit	Invitrogen	A-11008	1:200
Alexa fluor R-568	Rabbit	Invitrogen	A-11011	1:200
