# Supplementary material for: LCN2 promotes focal adhesion formation and invasion by stimulating c-Src activation
Source: J Cell Sci. 2025 Jun 5;138(11):jcs263663. doi: 10.1242/jcs.263663 (PMC12188317; doi:10.1242/jcs.263663)
Supplement: Supplementary file 1 [file joces-138-263663_review_history.pdf]

Journal of Cell Science

# LCN2 promotes focal adhesion formation and invasion by stimulating c-Src activation

Bhagya Shree Choudhary, Nazia Chaudhary, Bushra K. Khan, Aditi Vijan, Dibita Mandal, Leena Pilankar, Shubham Gawand, Prerana Uttankar, Megha Sharma, Anusha Shivshankar, Rinki Doloi, Neha Joshi, Manjula Das and Sorab N. Dalal
DOI: 10.1242/jcs.263663

**Editor**: Kathleen Green

**Review timeline**

| | |
|---|---|
| Original submission: | 4 June 2024 |
| Editorial decision: | 19 July 2024 |
| First revision received: | 4 December 2024 |
| Editorial decision: | 22 January 2025 |
| Second revision received: | 10 April 2025 |
| Accepted: | 11 April 2025 |

---

**Original submission**

First decision letter

MS ID#: JOCES/2024/262326

MS TITLE: LCN2 promotes focal adhesion formation and invasion by stimulating Src activation.

AUTHORS: Bhagya Shree Choudhary, Nazia N Chaudhary, Aditi N Vijan, Dibita Mandal, Leena Pilankar, Shubham Gawand, Bushra K Khan, Anusha Shivashankar, Rinki Doloi, Neha Joshi, Manjula Das, and Sorab N Dalal

ARTICLE TYPE: Research Article

Dear Dr. Dalal,

We have now reached a decision on the above manuscript.

To see the reviewers' reports and a copy of this decision letter, please go to: https://submit-jcs.biologists.org and click on the 'Manuscripts with Decisions' queue in the Author Area. (Corresponding author only has access to reviews.)

As you will see, the reviewers raise a number of substantial criticisms that prevent me from accepting the paper at this stage. They suggest, however, that a revised version might prove acceptable, if you can address their concerns. If you think that you can deal satisfactorily with the criticisms on revision, I would be pleased to see a revised manuscript. We would then return it to the reviewers.

**Response to authors**

The authors have addressed my queries and concerns and the manuscript is now acceptable for publication.

**Confidential note to editors**

© 2025. Published by The Company of Biologists under the terms of the Creative Commons Attribution License (https://creativecommons.org/licenses/by/4.0/).

In reviewing the raw blots, there appear to be several instances where images have been left-right flipped to generate the final figure, while other samples for the same blots were not similarly inverted. Also, in several instances the loading controls appear to be of separate blots from the protein of interest. (See Blots for Fig 3 for examples)

I do not know JCS policy regarding how these data are screened and what is acceptable, but perhaps these should be given a closer look to ensure they conform with the journals standards.

Reviewer 2

*Advance summary and potential significance to field*

In the revised manuscript by Choudhary, the authors have attempted to address the comments of other reviewers. However, the authors have failed to address my major comment: that there is conceptual issue in how the authors are synthesising their findings into a model.

*Comments for the author*

LCN2 can act both intracellularly in the cytoplasm, as well as extracellularly after secretion. The central issue is where LCN2 is working is contradictory within the authors own data. The authors provide convincing findings that in these cells and this assay, LCN2 is acting extracellularly:
-The overexpression of phenotype of LNC2 is abolished by the extracellular addition of a blocking antibody (Fig. 1I-K).
-The knockdown phenotype of LCN2 is rescued by exogenous recombinant LCN2 (Fig 2A-C).
-The clear effects of LCN2 overexpression of Src and Paxillin phosphorylation are completely abolished by extracellular LCN2 blocking antibody addition (Fig. 4G).

Collectively, this suggests that the extracellular function of LCN2 is what is required in this assay, and potentially that the intracellular pool is dispensable (which would be an exciting, surprising function). Yet, the authors present experiments on the intracellular function of LCN2 regulating actin filaments (Fig. 2), and a model (Fig. 8) where LCN2 is somehow crossing the lipid bilayer and coming from the extracellular domain into the cytoplasm to function with Ets1. Your data only support a role for LHN2 extracellularly.

The authors present no reconciliation of how this discrepancy can be addressed, other than addition of new discussion suggesting (untested) effects through EGFR, but which are stated to occur through intracellular contributions of LCN2. The experiments and model are therefore not supported by the data.

Minor
-The naming of conditions is confusing. Comparing names such as 'LCN2.1', 'LCN2.3' to 'shlcn2.1.3' or shlcn2.3.3' makes it tough work to understand what condition is used. Please simplify.
-When providing sample numbers for experiments, also include how many cells were quantified (for FA analyses) per biological replicate.

I restate my previous point below:

While the experiments provided are largely convincing, they are broad strokes of cancer biology pathways with major leaps between molecular dissection. For instance, it is somewhat straightforward to understand how controlling expression of the known Src phosphatase PTPN1 could influence directly Src phosphorylation and therefore focal adhesion and invasion dynamics. But how LCN2 impacts this is rather broad strokes. The experiments provided nicely show exogenous extracellular addition of recombinant LCN2 can rescue an endogenous LCN2 depletion. Conversely, extracellular addition of an LCN2 blocking antibody perturbs LCN2 function. This might suggest an extracellular function of LCN2. However, Fig 7 next somehow places LCN2 intracellularly functioning with Ets1 in the cytoplasm to control whether Etx1 translocates to the nucleus to control PTPN1 transcription. There is no dissection of how extracellular LCN2 can affect Ets1-dependent transcription. This is a major gap in the mechanism.

© 2025. Published by The Company of Biologists under the terms of the Creative Commons Attribution License (https://creativecommons.org/licenses/by/4.0/). 

I want to state that I do not think the authors need to provide this additional data. I think that there is enough in this paper already. However, how extracellular LCN2 could be regulating nuclear Ets1 needs to be at least discussed and the model revised. Is LCN2 acting through an extracellular receptor to transduce a signal to nuclear Ets1 or is LCN2 somehow crossing the lipid bilayer to do the job. At least some recognition of gaps in such molecular dissection should be provided.

---

**First revision**

Author response to reviewers' comments

**Response to authors**

The authors have addressed my queries and concerns and the manuscript is now acceptable for publication.

**Confidential note to editors**

In reviewing the raw blots, there appear to be several instances where images have been left-right flipped to generate the final figure, while other samples for the same blots were not similarly inverted. Also, in several instances the loading controls appear to be of separate blots from the protein of interest. (See Blots for Fig 3 for examples).
As indicated, the lanes referred to by the reviewer were run on a different gel with a different loading order. To generate the final figure, the blots were flipped so that the labelling of the figure would be consistent. We have labelled the original blots with the loading order in the pdf to eliminate any confusion and hope that this satisfies the reviewer and the journal.

I do not know JCS policy regarding how these data are screened and what is acceptable, but perhaps these should be given a closer look to ensure they conform with the journals standards.

We have included the raw data for all replicates of the blots (both a TIFF image and the original chemidoc files) and hope this satisfies the reviewer.

Reviewer 2: SUMMARY OF THE ADVANCE MADE IN THIS PAPER AND ITS POTENTIAL SIGNIFICANCE TO THE FIELD

In the revised manuscript by Choudhary, the authors have attempted to address the comments of other reviewers. However, the authors have failed to address my major comment: that there is conceptual issue in how the authors are synthesising their findings into a model.

SUGGESTIONS TO AUTHORS

LCN2 can act both intracellularly in the cytoplasm, as well as extracellularly after secretion. The central issue is where LCN2 is working is contradictory within the authors' own data. The authors provide convincing findings that in these cells and this assay, LCN2 is acting extracellularly:
-The overexpression of the phenotype of LNC2 is abolished by the extracellular addition of a blocking antibody (Fig. 1I-K).

The reviewer is correct to state that the addition of the blocking antibody inhibits function. However, multiple experiments have shown that LCN2 cycles between the extracellular space and is re-imported into cells through interaction between LCN2 and multiple cell surface receptors of LCN2 (Chakraborty et al., 2012; Devireddy et al., 2005; Devireddy et al., 2001). This secretion and subsequent re-import of LCN2 into cells is required for the ability of LCN2 to regulate intracellular iron levels. Under the conditions of low iron in the extracellular milieu, LCN2 import results in increased iron availability inside the cell (Chi et al., 2020; Devireddy et al., 2005). In contrast, an increase in intracellular iron levels can stimulate Ferroptosis, which is inhibited by the activation of iron export and a reduction of iron import (Gao et al., 2015; Ma et al., 2016). Our previous publication has shown that treatment with the antibody results in both an increase in the levels of intracellular iron (Chaudhary et al., 2021) and an increase in actin glutathionylation, a consequence

© 2025. Published by The Company of Biologists under the terms of the Creative Commons Attribution License (https://creativecommons.org/licenses/by/4.0/). 

of increased intracellular iron levels, resulting in a decrease in actin filament formation and invasion (Choudhary et al., 2023). Our work strongly suggests that the antibody prevents the interaction of extracellular LCN2 with its receptors, thereby preventing LCN2 transport into the cell, resulting in an increase in intracellular iron.

-The knockdown phenotype of LCN2 is rescued by exogenous recombinant LCN2 (Fig 2A-C).

The reviewer is correct in suggesting that the exogenous addition of LCN2 leads to the rescue of the knockdown phenotypes. However, we have previously demonstrated that the recombinant proteins enter the cell, presumably through interaction with the LCN2 receptors (Choudhary et al., 2023). Therefore, these proteins could regulate intracellular events. Further, we have demonstrated that an iron-binding defective mutant of LCN2 cannot rescue the effects of LCN2 knockdown in terms of invasion (Choudhary et al., 2023), but does support focal adhesion formation (this report). The export of iron from the cell presumably requires the secretion of LCN2 since the iron-binding mutant of LCN2 fails to export iron out of the cell. Further, the secreted LCN2 needs to be re-imported into the cell via the LCN2 receptor to ensure that the levels of intracellular iron are maintained. Finally, a previous report has suggested that a secretion defective LCN2 mutant can promote the recycling of activated EGFR to the cell border (Yammine et al., 2019) and there are multiple studies that suggest that activation of EGFR can promote the expression of multiple transcription factors including ETS1 (Phuchareon et al., 2015; Tetsu et al., 2015; Tetsu et al., 2016). These results support the argument that LCN2 has to both promote iron export by binding to iron and exporting it out of the cell and promote ETS1 expression to facilitate focal adhesion formation.

-The clear effects of LCN2 overexpression of Src and Paxillin phosphorylation are completely abolished by extracellular LCN2 blocking antibody addition (Fig. 4G).

This is correct. However, our previously published results suggest that the antibody blocks the interaction of LCN2 with the receptor, resulting in a decrease in ETS1 levels. As ETS1 also stimulates LCN2 expression, the subsequent decrease in intracellular LCN2 levels hampers the positive feedback loop, resulting in low levels of LCN2 and, therefore, inhibition of LCN2-dependent functions (Chaudhary et al., 2021). As the increase in ETS1 levels is responsible for the increase in Src and paxillin phosphorylation, the effect of the LCN2 antibody on these processes could be due to the ability of the antibody to decrease the intracellular pool of LCN2.

Collectively, this suggests that the extracellular function of LCN2 is what is required in this assay, and potentially that the intracellular pool is dispensable (which would be an exciting, surprising function). Yet, the authors present experiments on the intracellular function of LCN2 regulating actin filaments (Fig. 2), and a model (Fig. 8) where LCN2 is somehow crossing the lipid bilayer and coming from the extracellular domain into the cytoplasm to function with Ets1. Your data only support a role for LCN2 extracellularly.

Multiple lines of evidence suggest that LCN2 can enter cells by interacting with the LCN2 receptors on the cell surface (Chakraborty et al., 2012; Devireddy et al., 2005), presumably through endocytic mechanisms. This has been clarified in the manuscript and in the model, and we hope that this satisfies the reviewer.

The authors present no reconciliation of how this discrepancy can be addressed other than the addition of a new discussion suggesting (untested) effects through EGFR, which are stated to occur through intracellular contributions of LCN2. The experiments and model are, therefore, not supported by the data.

While the reviewer is correct in stating that we have not provided any direct evidence for an intracellular role of LCN2, we believe that based on the previously published data and our experiments, we cannot exclude an intracellular role for LCN2 for the following reasons:

1. Extracellular LCN2 can enter the cell via receptors present on the cell surface (Chakraborty et al., 2012; Devireddy et al., 2005), and our previous results have demonstrated that the exogenously added recombinant LCN2 proteins do enter the cell (Choudhary et al., 2023).

© 2025. Published by The Company of Biologists under the terms of the Creative Commons Attribution License (https://creativecommons.org/licenses/by/4.0/).

2. Despite the ability of the recombinant LCN2 proteins to enter the cell, the iron-binding mutant cannot promote invasion due to the inability of this mutant to bind to iron (Bao et al., 2010) and export iron out of the cell (Choudhary et al., 2023). At the very least, intracellular LCN2 is required to export iron out of the cell, as reported by other workers and results from our own laboratory (Chaudhary et al., 2021; Devireddy et al., 2005).

3. One possible explanation for the antibody experiments is that it only blocks extracellular functions. However, it is also equally possible that it prevents the interaction of LCN2 with the cell surface receptor, preventing its internalization.

4. A secretion defective LCN2 mutant can promote the recycling of activated EGFR to the cell border (Yammine et al., 2019), and there are multiple studies that suggest that activation of EGFR can promote the expression of multiple transcription factors, including ETS1 (Phuchareon et al., 2015; Tetsu et al., 2015; Tetsu et al., 2016).

5. We have generated a recombinant secretion defective mutant (LCN2Δss) of LCN2, which removes the signal sequence required for export, as described in (Yammine et al., 2019). We have treated HCT116 cells with either the vehicle control, the WT LCN2, the iron binding defective mutant of LCN2 or the secretion defective mutant of LCN2. We have demonstrated that all recombinant proteins enter the cell, and all of them stimulate phosphorylation of c-Src at Y416 and FAK at Y576/577. However, while WT LCN2 could stimulate invasion, in comparison to the vehicle control and the iron binding defective mutant, the secretion defective LCN2 mutant stimulated some invasion probably because it can still sequester iron inside the cell but cannot export iron out of the cell. These data are in supplementary figure 8, and we feel that this data strongly suggests that cellular LCN2 has a role in promoting focal adhesion formation as suggested by the model.

Based on these data, we feel that it is appropriate to suggest that there are intracellular functions of LCN2. However, we have modified the discussion to suggest that it is possible that most of the effects of LCN2 observed here could be attributed to the ability of extracellular LCN2 to bind to the LCN2 receptor resulting in altered signaling.

Minor
-The naming of conditions is confusing. Comparing names such as 'LCN2.1' and 'LCN2.3' to 'shlcn2.1.3' or shlcn2.3.3' makes it tough work to understand what condition is used. Please simplify.

The LCN2.1 and LCN2.3 are two different LCN2 over-expressing clones that stably express LCN2. This has been clarified in the manuscript. We have changed the names of the shRNA-derived LCN2 knockdown clones to shLCN2.1. and shLCN2.3 as they were made with different shRNA constructs, and we hope that this satisfies the reviewer.

-When providing sample numbers for experiments, also include how many cells were quantified (for FA analyses) per biological replicate.

We thank the reviewer for this suggestion and have added these to the methods section.

I restate my previous point below:

While the experiments provided are largely convincing, they are broad strokes of cancer biology pathways with major leaps between molecular dissection. For instance, it is somewhat straightforward to understand how controlling expression of the known Src phosphatase PTPN1 could influence directly Src phosphorylation and therefore focal adhesion and invasion dynamics. But how LCN2 impacts this is rather broad strokes. The experiments provided nicely show exogenous extracellular addition of recombinant LCN2 can rescue an endogenous LCN2 depletion. Conversely, extracellular addition of an LCN2 blocking antibody perturbs LCN2 function. This might suggest an extracellular function of LCN2. However, Fig 7 next somehow places LCN2 intracellularly functioning with Ets1 in the cytoplasm to control whether Ets1 translocates to the nucleus to control PTPN1 transcription. There is no dissection of how extracellular LCN2 can affect Ets1-dependent transcription. This is a major gap in the mechanism.

© 2025. Published by The Company of Biologists under the terms of the Creative Commons Attribution License (https://creativecommons.org/licenses/by/4.0/).

I want to state that I do not think the authors need to provide this additional data. I think that there is enough in this paper already. However, how extracellular LCN2 could be regulating nuclear Ets1 needs to be at least discussed and the model revised. Is LCN2 acting through an extracellular receptor to transduce a signal to nuclear Ets1, or is LCN2 somehow crossing the lipid bilayer to do the job? At least some recognition of gaps in such molecular dissection should be provided.

To address the reviewer's comments, we have added the receptor for LCN2 to the model to address the question of how exogenous LCN2 can cross the lipid bilayer to enter the cell as previously described in the literature (Chakraborty et al., 2012; Devireddy et al., 2005). The additional data we have added in supplementary figure 8 suggests that exogenously added LCN2 does enter the cell and, as reported previously, could stimulate Ets1 expression by promoting the transport of EGFR to the cell border (Phuchareon et al., 2015; Tetsu et al., 2015; Tetsu et al., 2016; Yammine et al., 2019). We do not wish to suggest that LCN2 stimulates the nuclear transport of Ets-1 and have modified the model accordingly.

References

**Bao, G., Clifton, M., Hoette, T. M., Mori, K., Deng, S. X., Qiu, A., Viltard, M., Williams, D., Paragas, N., Leete, T. et al.** (2010). Iron traffics in circulation bound to a siderocalin (Ngal)-catechol complex. *Nat Chem Biol* **6**, 602-9.

**Chakraborty, S., Kaur, S., Guha, S. and Batra, S. K.** (2012). The multifaceted roles of neutrophil gelatinase associated lipocalin (NGAL) in inflammation and cancer. *Biochim Biophys Acta* **1826**, 129-69.

**Chaudhary, N., Choudhary, B. S., Shah, S. G., Khapare, N., Dwivedi, N., Gaikwad, A., Joshi, N., Raichanna, J., Basu, S. and Gurjar, M.** (2021). Lipocalin 2 expression promotes tumor progression and therapy resistance by inhibiting ferroptosis in colorectal cancer. *International Journal of Cancer* **149**, 1495-1511.

**Chi, Y., Remsik, J., Kiseliovas, V., Derderian, C., Sener, U., Alghader, M., Saadeh, F., Nikishina, K., Bale, T., Iacobuzio-Donahue, C. et al.** (2020). Cancer cells deploy lipocalin-2 to collect limiting iron in leptomeningeal metastasis. *Science* **369**, 276-282.

**Choudhary, B. S., Chaudhary, N., Shah, M., Dwivedi, N., P, K. S., Das, M. and Dalal, S. N.** (2023). Lipocalin 2 inhibits actin glutathionylation to promote invasion and migration. *FEBS Lett* **597**, 1086-1097.

**Devireddy, L. R., Gazin, C., Zhu, X. and Green, M. R.** (2005). A cell-surface receptor for lipocalin 24p3 selectively mediates apoptosis and iron uptake. *Cell* **123**, 1293-305.

**Devireddy, L. R., Teodoro, J. G., Richard, F. A. and Green, M. R.** (2001). Induction of apoptosis by a secreted lipocalin that is transcriptionally regulated by IL-3 deprivation. *Science* **293**, 829-34.

**Gao, M., Monian, P., Quadri, N., Ramasamy, R. and Jiang, X.** (2015). Glutaminolysis and Transferrin Regulate Ferroptosis. *Mol Cell* **59**, 298-308.

**Ma, S., Henson, E. S., Chen, Y. and Gibson, S. B.** (2016). Ferroptosis is induced following siramesine and lapatinib treatment of breast cancer cells. *Cell Death Dis* **7**, e2307.

**Phuchareon, J., McCormick, F., Eisele, D. W. and Tetsu, O.** (2015). EGFR inhibition evokes innate drug resistance in lung cancer cells by preventing Akt activity and thus inactivating Ets-1 function. *Proc Natl Acad Sci U S A* **112**, E3855-63.

**Tetsu, O., Eisele, D. W. and McCormick, F.** (2015). Resistance to EGFR-targeted therapy by Ets-1 inactivation. *Cell Cycle* **14**, 3211-2.

**Tetsu, O., Phuchareon, J., Eisele, D. W. and McCormick, F.** (2016). ETS1 inactivation causes innate drug resistance to EGFR inhibitors. *Mol Cell Oncol* **3**, e1078924.

**Yammine, L., Zablocki, A., Baron, W., Terzi, F. and Gallazzini, M.** (2019). Lipocalin-2 Regulates Epidermal Growth Factor Receptor Intracellular Trafficking. *Cell Rep* **29**, 2067-2077 e6.

---

Second decision letter

MS ID#: jcs.263663R1

MS TITLE: LCN2 promotes focal adhesion formation and invasion by stimulating Src activation.

© 2025. Published by The Company of Biologists under the terms of the Creative Commons Attribution License (https://creativecommons.org/licenses/by/4.0/). 

AUTHORS: Bhagya Shree Choudhary; Nazia Chaudhary; Bushra K Khan; Aditi Vijan; Dibita Mandal; Leena Pilankar; Shubham Gawand; Anusha Shivshankar; Rinki Doloi; Neha Joshi; Manjula Das; Sorab Nariman Dalal

ARTICLE TYPE: Research Article

Dear Sorab,

We have now reached a decision on the above manuscript.

To see the reviewers' reports and a copy of this decision letter, please go to:

As you will see, one referee is satisfied with the response to previous critiques. However, the other referee states that the data shown do not support the contention that LCN2 is acting both intra-and extracellularly, and that the assay used only supports the extracellular function. In addition they have other concerns that would need to be addressed prior to acceptance.

In addition, both referees had questions about the presentation of data. These included a question regarding whether it is possible that some of the blots may have been flipped and also whether controls for loading came from different blots.

Given their questions, after discussion with the editorial office it was decided that we would like to have you provide all the raw data, including the blots from independent experiments at this stage, in order to avoid delaying publication at a later stage of the process. Please let us know if you have any questions regarding this request.

Please note that this will be the final opportunity to make major revisions to the work, and that we would need an enthusiastic response from both referees in the next round to proceed with the paper.

If you think that you can deal satisfactorily with the criticisms on revision, I would be pleased to see a revised manuscript. We would then return it to the reviewers.

---

**Second revision**

Author response to reviewers' comments

Reviewer 1

Previous work from this group has shown that the secreted, iron binding protein LCN2 promotes colorectal tumor cell invasion through iron dependent regulation of F-actin assembly via inhibition of actin glutathionylation. Herein, using both stable overexpression and knockdown approaches, they extend this analysis to report that LCN2 expression in HCT116 colon cancer cells also promotes increased focal adhesion formation and invasion, independent of its role in actin modification. Evidence is presented that LNC2 promotes elevated focal adhesion formation, independent of iron binding, through Src dependent paxillin phosphorylation and that the elevated Src activity is the result of LNC2- stimulated ETS1 transcription activity promoting PTP1B phosphatase expression leading to auto-activation of Src through dephosphorylation of Src Y527. While it is perhaps not surprising that focal adhesions and the associated F-actin filaments are coordinately altered to control invasion, the data herein suggesting that LCN2 regulates these two events through two independent pathways is quite interesting and generally well supported by the data.

A number of queries should be addressed.

Major
1. The authors argue that Src is regulating paxillin phosphorylation and thereby focal adhesion assembly, independent of FAK activation (FAK Y397 blots Fig 3A, D) (and then contradict this

© 2025. Published by The Company of Biologists under the terms of the Creative Commons Attribution License (https://creativecommons.org/licenses/by/4.0/). 

statement in the Discussion (second to last paragraph)). This would be highly unusual and therefore the authors need to present additional evidence that FAK is not involved (eg using a FAK inhibitor, RNAi) and discuss other possible mechanisms.

We thank the reviewer for this comment. We would like to clarify that we do not believe that FAK activation is not important for the increased invasion and focal adhesion formation and apologize for the fact that this was not clear in the manuscript. Previous reports have suggested that phosphorylation of FAK at Y576 and Y577 by c-Src is required for increased FAK activation. This leads to the formation of an active FAK-c-Src complex leading to increased phosphorylation of paxillin and focal adhesion formation (Calalb et al., 1995; Lietha et al., 2007). Our data suggests that these phosphorylation events are elevated in the HCT116 derived LCN2 over-expressing cells and decreased in the DLD1 derived LCN2 knockdown cells. These data have been added to figure 5 in the revised manuscript. The activation of c-Src drives these phosphorylation events leading to increased FAK activation and paxillin phosphorylation leading to focal adhesion formation. In addition, we have treated cells with the FAK inhibitor and have shown that inhibition of FAK decreases invasion and focal adhesion formation. These data are in supplementary figure 5 in the revised manuscript.

Does a pxn Y118F mutant block the effect? Also, the FAK blot shows the greatest variability between replicates. Could this be masking a small, but significant change? In general, most of the blots appear to be saturated which would make accurate quantitation difficult.

We thank the reviewer for these comments. Given the data on Src inhibition, we would suggest that paxillin phosphorylation at Y118 is required for focal adhesion formation which is consistent with previously reported data (Gonzalez Wusener et al., 2021). As far as the quantitation of the FAK data, we have performed these experiments on a BioRad chemidoc which is linear over 5 orders of magnitude and therefore do not think we have achieved saturation despite the density of the bands in the images. We do not believe that the differences are significant.

Minor related point, Fig 3E and F both show quantitation of the same paxillin data. Should one of the graphs be for FAK?

The reviewer is absolutely right and we apologize for the error and have changed the figure in the graph.

2. The src inhibitor was apparently added for 24 hrs (Fig 4). Do the observed changes in pax phosphorylation and FA formation occur at earlier timepoints or are we observing chronic/gene expression changes? Does washout reverse the effect?

This is an excellent point. We do the invasion assay by treating the cells with the inhibitor and then preparing a single-cell suspension and adding the cells to the upper chamber in serum-free media to perform the invasion assays, which go overnight. This effectively is a washout. Complete media is in the bottom chamber. The absence of serum in the upper chamber probably prevents c-Src activation preventing further downstream activation of the events leading to FA formation.

3. All of the experiments are performed on stable HCT116 derivatives either under or overexpressing LNC2 (previously characterized, as stated). Can the basic effects on FAs etc be recapitulated by transient manipulation of LNC2 in parental HCT116 cells or other cancer line types?

We thank the reviewer for this comment. The experiments in the paper demonstrate that the addition of recombinant LCN2 protein to the media results in a restoration of LCN2 functions in DLD1 derived LCN2 knockdown cells and that inhibiting LCN2 with a monoclonal antibody result in a reversal of LCN2 function in HCT116 derived LCN2 overexpressing cells. These experiments are in figure 1 and figure 2. However, we have also performed a transient knockdown of LCN2 in the LCN2 high DLD1 cell line and we observe a decrease in paxillin streak formation similar to those observed in the stable knockdown cells. These experiments are in supplementary figure 1 and we hope they satisfy the reviewer.

© 2025. Published by The Company of Biologists under the terms of the Creative Commons Attribution License (https://creativecommons.org/licenses/by/4.0/).

4. The authors should at least discuss the respective cross talk between the two LNC2 mechanism of action given that there is considerable evidence in the literature for focal adhesion and F-actin stress fiber codependence.

We thank the reviewer for this comment. As the reviewer correctly points out there is a wealth of literature suggesting that while focal adhesions promote actin polymerization, stress fiber formation also promotes focal adhesion maturation (reviewed in (Case and Waterman, 2015)). In the models used in this paper, we have demonstrated that the iron binding defective mutant of LCN2 can promote focal adhesion formation but not actin polymerization (Choudhary et al., 2023). However, there is evidence that suggests that actin fiber formation drives the formation of mature focal adhesions containing zyxin (Choi et al., 2008; Hirata et al., 2008). We have not performed staining for zyxin and it is possible that in the absence of actin filament polymerization, we do not observe the formation of mature focal adhesions. These points have been added to the discussion and we hope that they satisfy the reviewer.

Minor
1. I cannot even see the outline of the cells in many of the panels in Fig 1. Can phase images be added confirm that the cells are still spread etc? Is all F-actin and FAs really lost? This doesn't appear to be the case in the Supplementary figure equivalents.

We have changed the fluorescence images to greyscale images. These demonstrate that the cells are spread and we hope that this satisfies the reviewer.

2. The methods section is generally lacking in important treatment details (eg duration and amount of competing antibodies used, purity, amount and duration of exogenous LNC2 protein added etc.

We apologize for the lack of detail and have added these to the materials and methods. Please note that the antibody dilutions are listed in Supplementary tables 2 and 3.

3. The nomenclature for the modified HCT116 cell lines used is not intuitive. Please at least provide more information in the legends.

We apologize for the confusion and have added the required information in the figure legend.

4. Two citations are listed in Figure 7. What are they referring to? A legend for this figure is needed.

The legend has been modified (this is now figure 8 in the revised manuscript) and we hope that this satisfies the reviewer. The citations from the figure have been removed as they have been cited in the discussion section of the manuscript.

Reviewer 2 Advance Summary and Potential Significance to Field:

The manuscript by Choudhary examines the function of the iron-binding protein LCN2 in focal adhesion formation and invasion. The authors identify a novel iron-binding-independent function of LCN2 in regulating focal adhesion dynamics and invasion. This pathway involves LCN2 (even in an iron-binding- deficient-manner) somehow promoting Ets1 regulation of the expression of the tyrosine phosphatase PTPN1. PTPN1 in turn regulates the switch between Src phosphorylation on Y416 (autophosphorylation to be active) and Y527 (inactivation). This dynamic switch in Src activation by phosphorylation control in turn regulates focal adhesion formation and invasion of colorectal cancer cells.

Reviewer 2 Comments for the Author:
Major comments
While the experiments provided are largely convincing, they are broad strokes of cancer biology pathways with major leaps between molecular dissection. For instance, it is somewhat straightforward to understand how controlling expression of the known Src phosphatase PTPN1 could influence directly Src phosphorylation and therefore focal adhesion and invasion dynamics. But how LCN2 impacts this is rather broad strokes. The experiments provided nicely show exogenous extracellular addition of recombinant LCN2 can rescue an endogenous LCN2 depletion.

© 2025. Published by The Company of Biologists under the terms of the Creative Commons Attribution License (https://creativecommons.org/licenses/by/4.0/). 

Conversely, extracellular addition of an LCN2 blocking antibody perturbs LCN2 function. This might suggest an extracellular function of LCN2. However, Fig 7 next somehow places LCN2 intracellularly functioning with Ets1 in the cytoplasm to control whether Ets1 translocates to the nucleus to control PTPN1 transcription. There is no dissection of how extracellular LCN2 can affect Ets1-dependent transcription. This is a major gap in the mechanism. I want to state that I do not think the authors need to provide this additional data. I think that there is enough in this paper already. However, how extracellular LCN2 could be regulating nuclear Ets1 needs to be at least discussed and the model revised. Is LCN2 acting through an extracellular receptor to transduce a signal to nuclear Ets1 or is LCN2 somehow crossing the lipid bilayer to do the job. At least some recognition of gaps in such molecular dissection should be provided.

The reviewer is right to point out that there is a gap in molecular dissection. A possible explanation is that previous data has demonstrated that in a model of chronic kidney disease LCN2 stimulates the recycling of activated EGFR to the cell border (Yammine et al., 2019) leading to increased EGFR activity. Importantly, this is independent of LCN2 secretion as a LCN2 mutant that is not secreted can stimulate the recycling of EGFR. As EGFR activation leads to the activation of several downstream transcription factors including ETS1 leading to the hypothesis that ETS1 inhibition might be a way to overcome resistance to EGFR inhibition (Phuchareon et al., 2015; Tetsu et al., 2015; Tetsu et al., 2016), the increase in ETS1 levels in LCN2 high cells may be due to the increased cycling of EGFR to the cell border. Similarly, loss of LCN2 in oral cancer cell lines leads to a decrease in EGFR recycling and a decrease in invasion (Huang et al., 2023). These results suggest that one possible mechanism by which intracellular LCN2 might function is by regulating the localization and recycling of activated EGFR. These points have been added to the discussion.

Quantitation
Key quantitation is missing from some experiments. For instance, Fig 2D, SF3A, B.

As requested by the reviewer, we have added the quantitation for figure 2D and SF3A. These experiments are now in Supplementary figure 3. SF3B (now SF3H) just demonstrates that the fusion proteins are loaded at equal levels.

-For those experiments where quantitation is provided, all quantitation should provide all data points, not a summary only as a bar graph. In addition, indicate sample numbers (how many replicates/samples per condition, not just experiments).

We have modified the figures accordingly and hope that this satisfies the reviewer.

Minor points
The acronym PTPCD1 is used for control samples. What is this?

This is the vector control for the over-expression analysis which we have used to distinguish it from the vector control for the knockdown construct. We apologize for the confusion and have added this information to the figure legend.

Reviewer 3 Advance Summary and Potential Significance to Field:
Lipocalin 2(LCN2) promotes cancer cell migration and invasion. In this manuscript, the authors report a role for the LCN2-ETS1-PTP1B-Src pathway in regulating cell focal adhesions. Overall, the results support the conclusion and will be of interest to the field.

Reviewer 3 Comments for the Author:

In my opinion, the manuscript will be suitable for publication in Journal of Cell Science if the following question is addressed: Can overexpression of constitutively active Src, ETS1, or PTP1B rescue the phenotype of focal adhesion, migration, and invasion caused by LCN2 knockdown?

This is an excellent suggestion and we have overexpressed constitutively active c-Src in the LCN2 low HCT116 cells. These data are now in Supplementary figure 4.

Bibliography

© 2025. Published by The Company of Biologists under the terms of the Creative Commons Attribution License (https://creativecommons.org/licenses/by/4.0/).

**Calalb, M. B., Polte, T. R. and Hanks, S. K.** (1995). Tyrosine phosphorylation of focal adhesion kinase at sites in the catalytic domain regulates kinase activity: a role for Src family kinases. *Mol Cell Biol* **15**, 954-63.
**Case, L. B. and Waterman, C. M.** (2015). Integration of actin dynamics and cell adhesion by a three-dimensional, mechanosensitive molecular clutch. *Nat Cell Biol* **17**, 955-63.
**Choi, C. K., Vicente-Manzanares, M., Zareno, J., Whitmore, L. A., Mogilner, A. and Horwitz, A. R.** (2008). Actin and alpha-actinin orchestrate the assembly and maturation of nascent adhesions in a myosin II motor-independent manner. *Nat Cell Biol* **10**, 1039-50.
**Choudhary, B. S., Chaudhary, N., Shah, M., Dwivedi, N., P, K. S., Das, M. and Dalal, S. N.** (2023). Lipocalin 2 inhibits actin glutathionylation to promote invasion and migration. *FEBS Lett* **597**, 1086-1097.
**Gonzalez Wusener, A. E., Gonzalez, A., Perez Collado, M. E., Maza, M. R., General, I. J. and Arregui, C. O.** (2021). Protein tyrosine phosphatase 1B targets focal adhesion kinase and paxillin in cell-matrix adhesions. *J Cell Sci* **134**.
**Hirata, H., Tatsumi, H. and Sokabe, M.** (2008). Mechanical forces facilitate actin polymerization at focal adhesions in a zyxin-dependent manner. *J Cell Sci* **121**, 2795-804.
**Huang, Z., Rui, X., Yi, C., Chen, Y., Chen, R., Liang, Y., Wang, Y., Yao, W., Xu, X. and Huang, Z.** (2023). Silencing LCN2 suppresses oral squamous cell carcinoma progression by reducing EGFR signal activation and recycling. *Journal of Experimental & Clinical Cancer Research* **42**, 1-22.
**Lietha, D., Cai, X., Ceccarelli, D. F., Li, Y., Schaller, M. D. and Eck, M. J.** (2007). Structural basis for the autoinhibition of focal adhesion kinase. *Cell* **129**, 1177-87.
**Phuchareon, J., McCormick, F., Eisele, D. W. and Tetsu, O.** (2015). EGFR inhibition evokes innate drug resistance in lung cancer cells by preventing Akt activity and thus inactivating Ets-1 function. *Proc Natl Acad Sci U S A* **112**, E3855-63.
**Tetsu, O., Eisele, D. W. and McCormick, F.** (2015). Resistance to EGFR-targeted therapy by Ets-1 inactivation. *Cell Cycle* **14**, 3211-2.
**Tetsu, O., Phuchareon, J., Eisele, D. W. and McCormick, F.** (2016). ETS1 inactivation causes innate drug resistance to EGFR inhibitors. *Mol Cell Oncol* **3**, e1078924.
**Yammine, L., Zablocki, A., Baron, W., Terzi, F. and Gallazzini, M.** (2019). Lipocalin-2 Regulates Epidermal Growth Factor Receptor Intracellular Trafficking. *Cell Rep* **29**, 2067-2077 e6.

---

Third decision letter

MS ID#: jcs.263663R2

MS Title: LCN2 promotes focal adhesion formation and invasion by stimulating Src activation.

Authors: Bhagya Shree Choudhary; Nazia Chaudhary; Bushra K Khan; Aditi Vijan; Dibita Mandal; Leena Pilankar; Shubham Gawand; Prerana Uttankar; Megha Sharma; Anusha Shivshankar; Rinki Doloi; Neha Joshi; Manjula Das; Sorab Nariman Dalal

Article Type: Research Article

Dear Dr Dalal,

I am happy to tell you that your manuscript has been provisionally accepted for publication in Journal of Cell Science, pending publication integrity checks.

© 2025. Published by The Company of Biologists under the terms of the Creative Commons Attribution License (https://creativecommons.org/licenses/by/4.0/).
